# MiR-122 regulates liver tolerance

**DOI:** 10.1016/j.jhepr.2026.102020

**Published:** 2026-08-29

**Authors:** Maytal Gefen, Shanny Layani, Emma Klahr, Zohar Shemuelian, Johannes Brandi, Thomas Jacobs, Christiane Steeg, Lorenz Adlung, Christoph Kilian, Merav Ordan, Oren Gordon, Nathalie Abudi, Rinat Abramovitch, Yuval Nevo, Sharona Elgavish, Hadar Benyamini, Jonathan Monin, Elina Zorde Khvalevsky, Nofar Rosenberg, Aurelia Markezana, Dayana Yaish, Adi S. Yehezkel, Alina Simerzin, Mila Rivkin, Osher Amran, Daniel S. Goldenberg, Amnon Peled, Jonathan Axelrod, Jacob Rachmilewitz, Samuel Huber, Nicola Gagliani, Christoph Schramm, Sören Weidemann, Johannes Herkel, Antonella Carambia, Rifaat Safadi, Abed Khalaileh, Ashraf Imam, Daniela Heide, Jenny Hetzer, Stefan Rose-John, Mathias Heikenwälder, Hilla Giladi, Eithan Galun

**Affiliations:** 1 Goldyne Savad Institute of Gene Therapy, Hadassah Hebrew University Hospital, Jerusalem, Israel; 2 Protozoa Immunology, Bernhard Nocht Institute for Tropical Medicine, Hamburg, Germany; 3 University Medical Centre Hamburg-Eppendorf, Hamburg, Germany; 4 The Wohl Institute for Translational Medicine, Human Biology Research Center, Hadassah University Medical Center, Jerusalem, Israel; 5 Computation Center, The Hebrew University of Jerusalem, Jerusalem, Israel; 6 Institute for Inflammation and Carcinogenesis, Hamburg Center for Translational Immunology, University Medical Center Hamburg-Eppendorf, Hamburg, Germany; 7 Liver institute Hadassah Hebrew University Hospital, Jerusalem, Israel; 8 Department of Surgery Hadassah Hebrew University Hospital, Jerusalem, Israel; 9 Division of Chronic Inflammation and Cancer, German Cancer Research Centre Heidelberg (DKFZ), Heidelberg, Germany; 10 The M3 Research Center, Medical Faculty, University Clinic Tübingen (UKT), Tübingen, Germany; 11 Department of Biochemistry, University of Kiel, Kiel, Germany

**Keywords:** Immune homeostasis, Autoimmune liver disease, Innate immunity, Chronic liver diseases, Fibrotic pathways, Pro-inflammatory cytokines, Immune checkpoints, Macrophage activation, Toll-like receptors (TLRs), NF-κB signaling, Immune activation, Gut–liver axis, Microbiome

## Abstract

**Background & Aims:**

The liver is an immune-privileged organ, yet it needs to overcome infections. We aimed to explore the role of the liver-specific microRNA miR-122 in liver immune tolerance.

**Methods:**

We developed a miR-122 knockout (KO) mouse model and assessed immune, inflammatory, and fibrogenic responses from early postnatal stages. Innate and adaptive immune alterations were studied in miR-122 KO mice. The transcriptomic profiles of patients with autoimmune hepatitis (AIH) were compared with those of these KO mice.

**Results:**

MiR-122 KO mice developed liver inflammation and fibrosis as early as 2 weeks of age. An inverse correlation between miR-122 expression and inflammation/fibrosis was observed in both KO mice (*p* <0.01in both at Weeks 2 [n = 6] and 3 [n = 6] of KO mice age) and human AIH samples (*p* <0.01, n = 9). KO mice shared transcriptomic and phenotypic features with human AIH. RNA sequencing identified more than 5,000 differentially expressed genes between wild-type and KO mice, including upregulated chemokines, immune checkpoints, and pro-fibrotic markers. IRF2, a TLR3 transcription factor, was identified as a novel direct target of miR-122.

**Conclusions:**

MiR-122 is a key regulator of liver immune tolerance, modulating inflammation and fibrosis via distinct pathways. Its absence enhances immune activation and accelerates fibrosis. These findings identify miR-122 as a promising therapeutic target for autoimmune liver diseases.

**Impact and implications:**

The liver is an immune-privileged organ, an adaptation likely evolved to prevent robust immune responses against material absorbed from the gastrointestinal tract. This tolerance creates a sanctuary for pathogens—including bacteria, hepatitis viruses, and parasites such as *Plasmodium*—within the liver parenchyma and similarly permits the development of primary liver malignancy and metastatic seeding. We found that miR-122 regulates hepatic immune tolerance, positioning miR-122 modulation as a potential therapeutic strategy for infectious, autoimmune, malignant, and metastatic liver diseases.

## Highlights

• MiR-122 regulates liver immune privilege.
• MiR-122 influences liver inflammation.
• MiR-122 deficiency causes fibrosis.

## Graphical abstract

**Figure:**
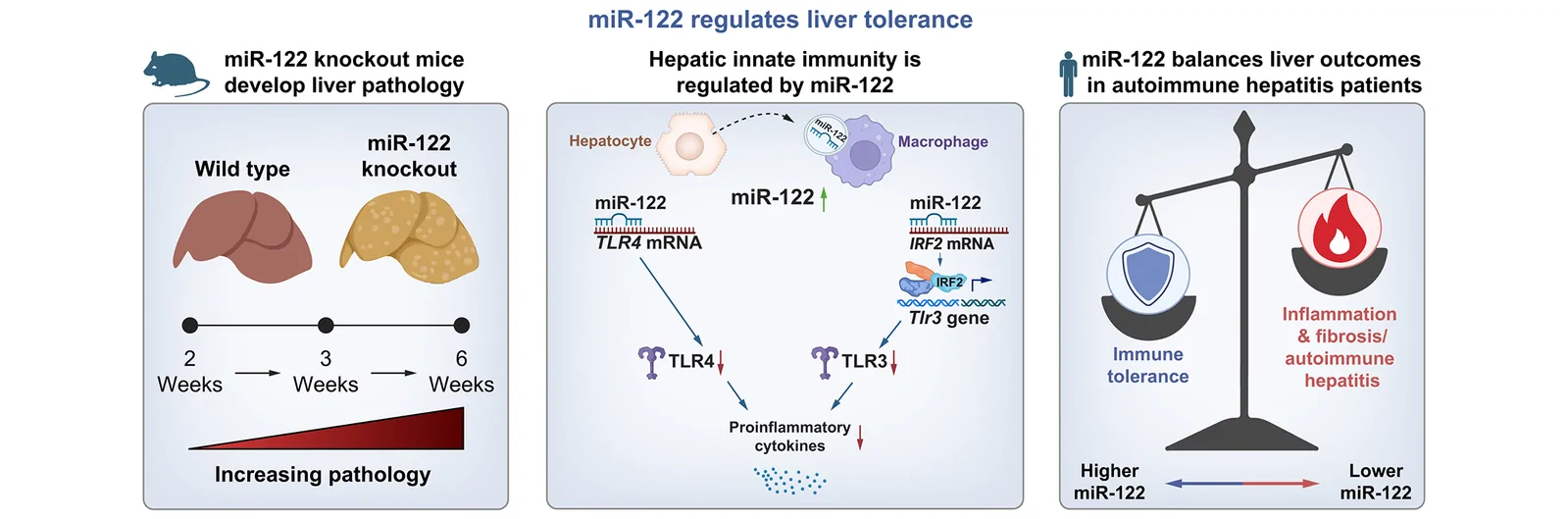

## Introduction

The liver microenvironment must be balanced and steadily equilibrated between immune responses and immune tolerance.^1^ On the one hand, the liver must maintain immune tolerance toward gut-derived material absorbed through the gastrointestinal tract. On the other hand, it must react against invading pathogens to protect its tissue integrity and function. The unique tolerogenic liver immune microenvironment enables liver transplantation without human leukocyte antigen (HLA) matching.^2^ However, it also makes the liver a preferred site for metastases.[2], [3] The mechanisms enabling this immune tolerance are only partially understood.[4], [5] We hypothesized that an efficient and comprehensive regulatory mechanism should involve concurrent regulation of different arms of the immune system and that, accordingly, a microRNA that is expressed at high levels in the liver and is capable of simultaneously suppressing genes and pathways may play a central role in the immune tolerance process.^6^ One potential microRNA that could fulfill this assignment is the liver-specific miR-122, which is expressed at over 100,000 copies per hepatocyte and constitutes 70% of all microRNAs contained in the liver.^7^ MiR-122, produced by hepatocytes, is secreted^8^ and can bind messages of target genes in adjacent and remote cells reached via the circulation. We were interested in understanding the mechanism underlying the liver microenvironment’s balance between the liver’s mission of combating infection and tolerating microbiome flushed from the gastrointestinal tract. Inflammatory mediators are central to anti-infection modalities. One example of a central mediator of inflammation and immunity in the liver is TNFα.^9^ We have previously reported that TNFα is central to liver immunity and inflammation[10], [11] and, interestingly, at the same time, TNFα mediates miR-122 expression.^8^ Previous studies[12], [13] have shown, and we confirm in this study, that hepatocellular carcinoma (HCC) develops in the miR-122 knockout (KO) mouse model. Knowing that HCC develops on the background of chronic inflammation, we wondered when all these changes develop and how they could contribute to HCC development. This circuitry led us to investigate postnatal (weeks 2–6 of mouse life) events in the early liver microenvironment that could also affect the development of anemia^14^ or HCC in miR-122 KO mice, as previously reported.^15^ To understand this immune/inflammatory hepatic microenvironment, we generated a new constitutive miR-122 KO mouse using a CRISPR/Cas9 system, omitting a very short genomic region of 59 nt, as opposed to previous studies in which a significantly larger genomic region was excised, which could cause non-relevant off-target effects. We investigated alterations in innate and adaptive immune cells in the liver during early postnatal life and monitored fibrosis development from 2 weeks of age. Herein, we report on mechanisms encompassing the role of miR-122 in regulating liver immune tolerance. Interestingly, in miR-122 KO mice, fibrosis and inflammation are not sequential: inflammation initiates first, followed by fibrosis.^16^ This regulation involves the direct and indirect effects of miR-122 on known liver and systemic immune regulators and inflammatory pathways, many of which were reported in specific cases as miR-122 direct and indirect targets. So far, a longitudinal assessment of the whole organ from early life onwards has not been conducted. MiR-122 was shown to execute tumor suppressive effects,[17], [18] and we confirm these findings in our data. Furthermore, to the list of immune genes directly targeted by miR-122, we add IRF2, a positive regulator of Toll-like receptor 3 (TLR3), thereby supporting our overall hypothesis that miR-122 is an immune attenuator. The significance of our findings is underscored by the possibility that manipulating miR-122 levels could have clinical significance. It would be beneficial to upregulate miR-122 to suppress tumors in liver metastasis or liver autoimmune diseases.

## Materials and methods

### Mice and ethical approvals

MiR-122 KO mice (C57BL/6miR-122em2Hhtg) were generated via CRISPR/Cas9-mediated deletion of the miR-122 genomic region (chr18:65,381,932-65,381,997). Full genotyping and colony maintenance protocols are available in the Supplementary Materials and Methods and in Table S1.

### *In vivo* experimental procedures

For MRI monitoring, mice were anesthetized and scanned using a 7T cryogen-free MR scanner (MR Solutions, Guildford, UK) to assess liver pathology. MiR-122 inhibition was achieved via hydrodynamic tail vein injection of antagomiR-122 (7 μg/mouse), followed by lipopolysaccharide (LPS) administration (1 μg/kg, Sigma-Aldrich, Rehovot, Israel). Liver and blood samples were harvested after treatment for analysis. Macrophage depletion and T-cell depletion protocols used clodronate liposomes and anti-CD4/CD8 antibodies, respectively, as detailed in the Supplementary Materials and Methods.

### Microbiome depletion

Breeding pairs were treated with an antibiotic cocktail (vancomycin, imipenem, and neomycin) from gestation through weaning. Fecal microbiome quantification was performed using the ZymoBIOMICS DNA Miniprep Kit (Irvine, California, USA).

### Molecular and cellular analyses

RNA extraction, quantitative PCR, RNA sequencing (RNAseq), and flow cytometry were performed using established protocols (Supplementary Materials and Methods and Table S1–S3). RNA integrity was verified (RIN ≥9). For histopathology, liver sections were processed for H&E, Sirius Red staining, or TUNEL assay.

### Statistics

Data were analyzed using GraphPad Prism (v6.02) with Student’s *t* tests or Mann–Whitney tests, as appropriate. A *p* ≤0.05 was considered statistically significant (San Diego, California, USA).

See the Supplementary Materials and Methods for further details.

## Results

### MiR-122 in human and mouse chronic AIH

Chronic autoimmune hepatitis (AIH) develops when the liver’s tolerance is breached.^19^ We first analyzed the correlation between liver inflammation and fibrosis in patients with AIH (Fig. S1A and B), as well as miR-122 expression levels in the liver and found significant negative correlations with both.

Moreover, in a mouse model of AIH,^20^ we found a significant decrease in mature miR-122 levels in serum and liver, as well as reduced levels of miR-122 precursor and pri-miR-122 in the inflamed liver tissue (Fig. S2). To further investigate the effect of miR-122 on the immune system, we injected mice with antagomir-122, which represses miR-122 activity, followed by LPS injection to induce an acute immune response (Fig. S3A and B). RNAseq was performed on the livers of these mice, revealing many differentially expressed genes (Fig. S3C). Using KEGG and INGENUITY discovery tools, we found a significantly increased effect on the innate immune system, as indicated by the gene set enrichment analysis (GSEA) score (Fig. S3D). A significant increase in TLR3 expression (log_2_ fold change 1.144, *p* <0.0006) was found. Because the liver is a known sanctuary for viral hepatitis, including chronic HCV infection, the possibility that miR-122 regulates TLR3, a sensor of double-stranded RNA, is important for understanding its role in immune regulation.^21^ Moreover, an enrichment of genes associated with interferon-α and interferon-γ (IFNγ) responses was found, supporting the idea of innate immune activation in the absence of miR-122 (Fig. S3E). We further proved that IRF2 is a regulator of TLR3 (Fig. S3F–H) and a direct target of miR-122 (Fig. S3I–K). A schematic illustration of IRF2 at the genomic site of TLR3 is shown in Fig. S3L.

### Liver microenvironment of miR-122 KO mice

To further investigate the role of miR-122 in liver tolerance, we generated miR-122 KO mice by deleting a 59-nt genomic region coding for miR-122 (Fig. S4A and B), resulting in a complete shutdown of miR-122 expression (Fig. S4C). In wild-type (WT) mice, the gut microbiome develops already at a very early stage of life.^22^ This led us to expect changes in the liver microenvironment of miR-122 KO mice already early in life as well. We investigated the phenotype of these mice from 2 weeks postnatally onward (Fig. 1). As shown in Fig. 1A, liver inflammation and fibrosis begin at 2 weeks and persist through Week 3. By 6 weeks of age, significant liver inflammation and fibrosis can be found in miR-122 KO mice. At 6 weeks of age, both CD4 and CD8 cells increased in the livers of miR-122 KO mice. The inflammatory process was associated with enhanced macrophage/monocyte liver infiltration and increased hepatic neutrophil and lymphocyte count (Fig. 1C), which was also associated with impaired liver function, indicating parenchymal injury (Fig. 1B). This pro-inflammatory, pro-fibrotic effect in the livers of miR-122 KO mice was associated with elevated hepatocyte apoptosis (Fig. 1C).

**Fig. 1:**
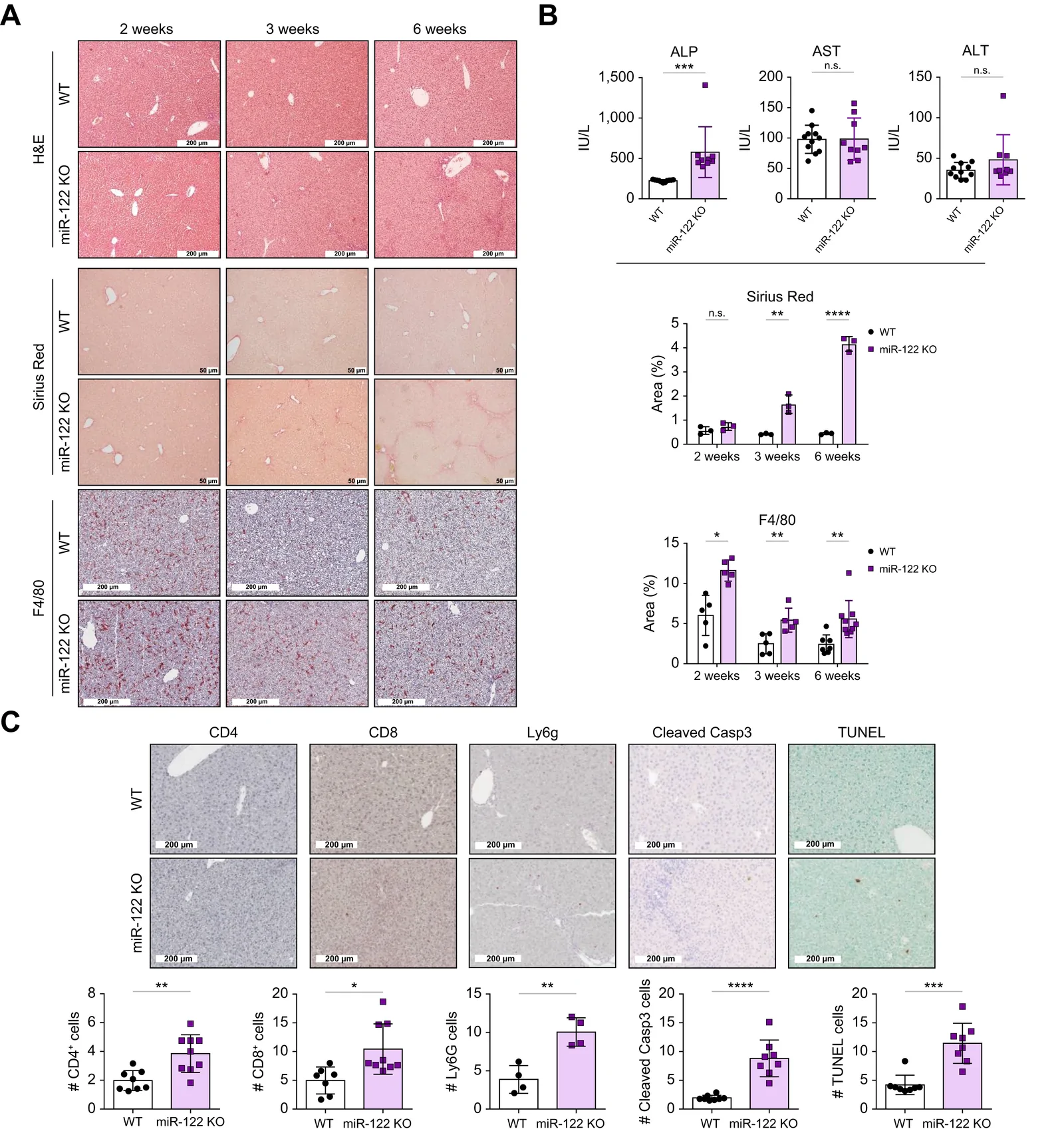
miR-122 KO mice exhibit enhanced inflammation and fibrosis from early age. (A) Liver histology of miR-122 KO mice and WT controls. H&E stain, fibrosis (Sirius Red), and macrophages (F4/80) at 2, 3, and 6 weeks of age. (B) Liver damage markers (ALP, SGOT (AST), and SGPT (ALT)) measured in the serum of miR-122 KO mice. (C) Liver histology at 6 weeks of age, with additional markers for inflammation and immune response, including CD4 and CD8, as well as neutrophils (Ly6g) and apoptosis (TUNEL and cleaved caspase 3). Quantification was performed as number of positive cells per field. ∗*p* <0.05, ∗∗*p* <0.01, ∗∗∗*p* <0.001, ∗∗∗∗*p* <0.0001. ALP, alkaline phosphatase; KO, knockout; SGOT(AST), serum glutamic-oxaloacetic transaminase; SGPT(ALT), serum glutamic pyruvic transaminase; WT, wild type.

To further investigate the nature of these pro-inflammatory, pro-fibrotic processes, we performed liver RNAseq over time, which revealed significant overall changes in miR-122 KO livers (Fig. S4D). More than 4,000 genes were significantly differentially expressed (adjusted *p* <0.05) at the early age of 2 weeks, and approximately 5,000 genes at the 3- and 6-week time points, emphasizing the age-dependent dynamics of the posttranscriptional regulation of genes by miR-122. Of these genes, a significant number were found to regulate fibrosis and the immune response and to be potential direct targets of miR-122 (Table S4). At 35 weeks of age, HCC began to develop in miR-122 KO mice, and by 78 weeks, most mice developed sizable HCC (29/42, size >2mm), consistent with previous studies[15], [23] (Fig. S4E). To confirm the efficacy of miR-122 knockout in mice, we assessed the expression of the known miR-122 target gene hemojuvelin (*Hjv*)^14^ and its indirect effect on hepcidin (*Hamp*). Both *Hjv* and *Hamp* levels were increased, as determined by reverse-transcription PCR (RT-PCR) and single-cell RNA sequencing (scRNAseq) (Fig. S4F and G), confirming the miR-122 KO functionality.

### Role of miR-122 in liver cellular and innate immune responses

In the livers of miR-122 KO mice, we observed significant changes in gene expression (Fig. S4D). Analyzing these changes directed our attention to changes in inflammation/immune cells, both histologically (Fig. 1B,C) and in their gene expression (Fig. 2A). Some of the changes were associated with genes known to be direct targets of miR-122. Changes were apparent in T cells, macrophages/monocytes, B cells, and neutrophils (Fig. 2A). In B cells, an increase in Blnk and CD320 was observed, as indicated by RNAseq; the latter is a known miR-122 target in humans and mice.^24^ To further investigate T-cell-related changes, we performed a multiparameter FACS analysis. By assessing immune checkpoint molecules on T cells, we observed a slight reduction in CD4 (Fig. 2B) and a pronounced and significant reduction on CD8 cells, including PD-1, CTLA-4, Galectin-9, TIM-3, and LAG-3 (Fig. 2B). These changes are associated with an enhanced immune activation response of CD8 cells in the liver,^25^ as shown in Fig. 1, Fig. 2. In addition to the apparent changes in the cellular immune microenvironment, we observed an overall upregulation of chemokines and chemokine receptors, as examined by RNAseq, which may also contribute to the increase in immune and inflammatory cell migration into the liver^26^ and to liver fibrosis, including CXCL12 and CXCR4^27^ (Fig. 2C,D). To further understand the changes in the functionality of CD8 cells, we performed scRNAseq of CD8 cells from 2- and 6-week-old miR-122 KO and WT mice. We identified increased CD8 T-cell receptor (TCR) signaling and IL-12 signaling (Fig. S5A). This observation recapitulates the association between miR-122 and IL-12a (Fig. S5B and C) in human livers, indicating immune activation of the liver in miR-122 KO mice. Moreover, increased expression of IL-12A (log_2_ fold change 3.041, *p* = 6.6E-20) and its receptor IL-12RB1 was found in B cells, and the expression of IL-12RB2 was found in T, NK, and NK-T cells, as shown in scRNAseq clustering (Fig. S5D), underlying the communication between these immune cells. MiR-122 KO mice show an increase in ALP without an increase in hepatocellular enzymes, in line with a strong upregulation of the Alpl transcript encoding the ALP enzyme, and with the known fact that Alpl is a direct miR-122 target (in both mice and humans). In addition, miR-122 KO mice already exhibit a ductular reaction at an early age, with enhanced CK19 expression (data not shown). ALP is associated with the development of ductular reaction in both mice and humans.^28^

**Fig. 2:**
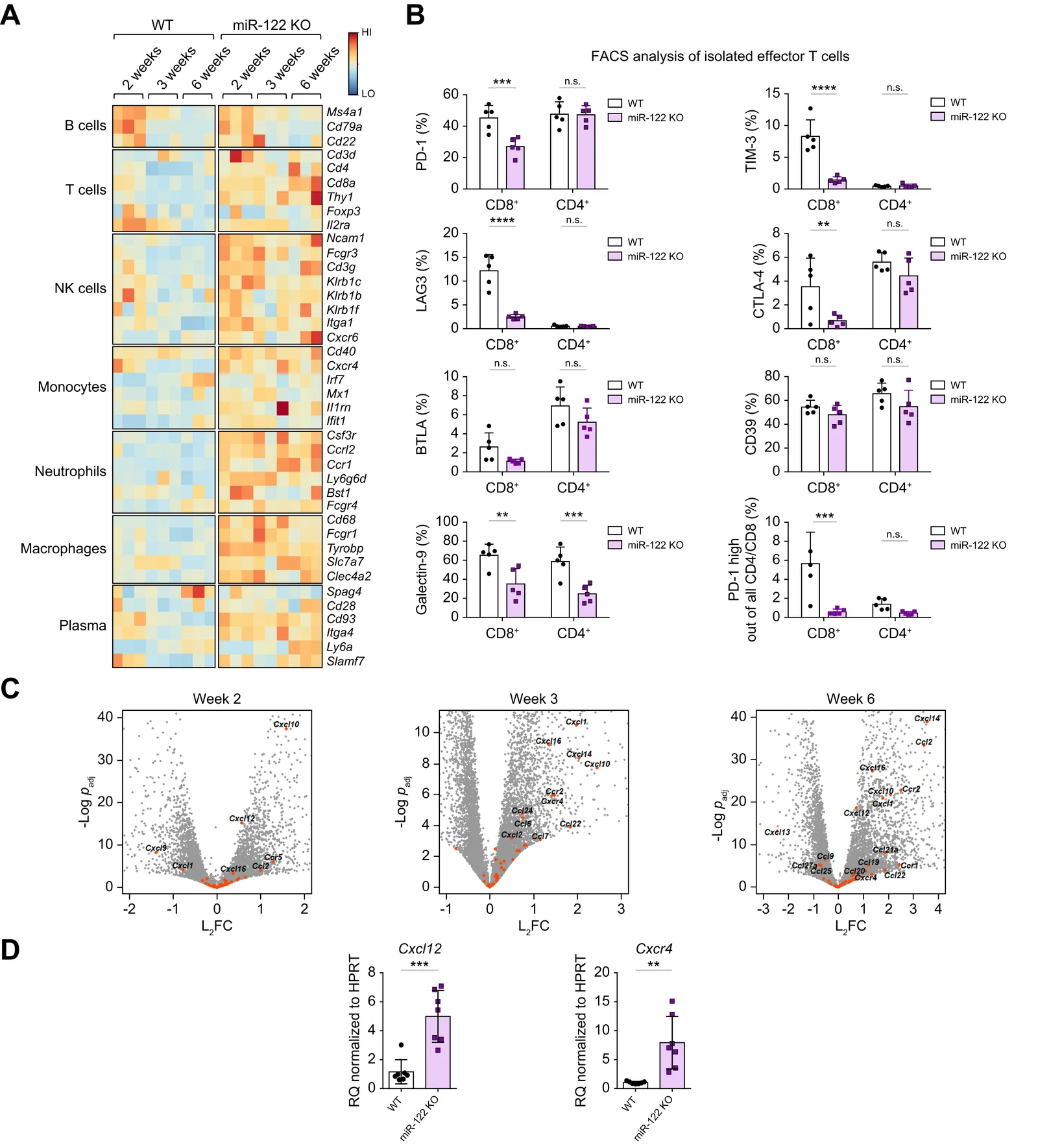
Enhanced adaptive immune response in miR-122 KO mice. (A) Heatmap of unsupervised genes associated with immune cells in the livers of miR-122 KO mice compared with WT controls at 2, 3, and 6 weeks of age. (B) FACS immunophenotyping analysis of CD25^+^ CD62L^-^ effector T-cell population in immune cells isolated from the livers of miR-122 KO mice compared with WT controls at 6 weeks of age. (C) Volcano plot of differentially expressed genes in miR-122 KO mice compared with WT controls, at 2, 3, and 6 weeks of age. Chemokine-associated genes are indicated in orange. (D) Real-time PCR confirmation of differentially expressed chemokine-associated genes in miR-122 KO mice compared with WT controls, at 6 weeks of age. ∗∗*p* <0.01, ∗∗∗*p* <0.001, ∗∗∗∗*p* <0.0001. KO, knockout; WT, wild type.

In addition to immune/inflammatory cellular activation, it is important to determine whether the innate immune system participates in the immune activation of the livers of miR-122 KO mice. A gene enrichment analysis (Fig. 3A,B) showed increased TNFα signaling, including enhanced NF-κB transcript levels. Cellular and nuclear expression of p65 (transducing NF-κB signaling) is increased in hepatocytes and cholangiocytes of miR-122 KO mice (Fig. 3C). The enhanced nuclear RelA signal is associated with an overall increase in TNFα, IL-1β, and IL-6 expression (Fig. 3D), paralleling the expression of IFNγ (Fig. 3D). TNFα and IL-1β activate NF-κB signaling, and downstream of IL-6, pSTAT3 activation can be observed, both enhanced in liver parenchymal and non-parenchymal cells (Fig. 3C,E). pSTAT3 levels are increased at both the 3- and 6-week time points, supporting our hypothesis that the lack of miR-122 leads to increased IL-6 and its activated pathways, as well as the enhancement of other potential inflammatory signals. Furthermore, IL6 shows an increase at 2 and 3 weeks in the liver of miR-122 KO mice (Fig. 3D). IL-6 plays a central role in liver physiology, particularly in both regeneration and immune regulation.^29^ MiR-122, produced by hepatocytes, is secreted into the liver microenvironment,^8^ targeting numerous immune mediators on adjacent macrophages, including TLR2 and TLR4.^30^ In addition, as shown in this study, it also indirectly targets TLR3. The signaling downstream of these TLRs is attenuated in miR-122-proficient mice, and upon miR-122 elimination, this arm of the innate immune response is enhanced. This observation also explains the enhanced inflammation and fibrosis phenotype we observed in Fig. 1A,C. In addition, IFNγ enhancement is associated with liver inflammation in immune-mediated liver disease, such as primary sclerosing cholangitis.^31^ This potentially suggests that upon elimination of miR-122, a significant immune imbalance develops in the liver.

**Fig. 3:**
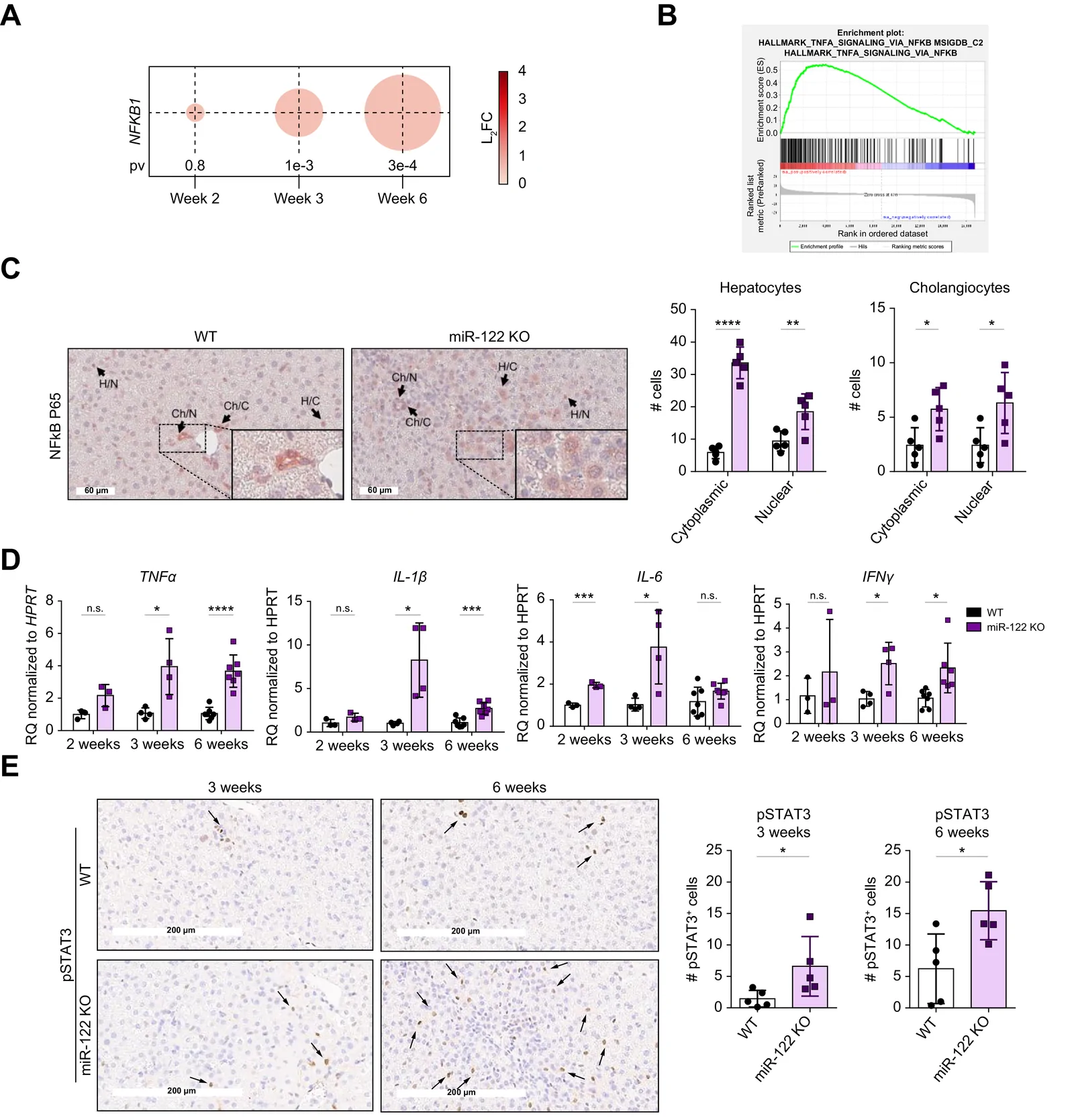
miR-122 KO mice encounter enhanced immune phenotype. (A) RNAseq gene expression analysis of the livers of miR-122 KO mice compared with WT controls at 2, 3, and 6 weeks of age, showing the significance (pv) and fold change (L2FC) of the NF-κB1 gene encoding the NF-κB protein. (B) GSEA of the TNFα signaling pathway via NF-κB in miR-122 KO mice. (C) NF-κB P65 immunohistochemical staining in miR-122 KO mice and WT controls at 6 weeks of age. Quantification of stained hepatocytes (H) and cholangiocytes (Ch), with nuclear (N) and cytoplasmatic (C) localization. (D) Relative gene expression measured by real-time PCR of downstream mediators of inflammation TNFα, IL-1β, IL-6 and IFNγ, measured in the livers of miR-122 KO mice and WT controls at 2, 3, and 6 weeks of age. (E) pSTAT3 immunohistochemical staining in miR-122 KO mice and WT controls at 3 and 6 weeks of age. ∗*p* <0.05, ∗∗*p* <0.01, ∗∗∗*p* <0.001, ∗∗∗∗*p* <0.0001. GSEA, gene set enrichment analysis; KO, knockout; RNAseq, RNA sequencing; WT, wild type.

### Regulation of TLR3 by miR-122

TLRs are important immune sensors of invading pathogens, and TNFα plays an important role in one of their effector pathways.^32^ The microbiota is an important constituent player in the gut–liver axis,^33^ and a non-functional gastrointestinal barrier leads to an elevated transmigration of bacteria into the circulation. Interestingly, it was recently reported that miR-122 directly regulates TLR4,^34^ the immune sensor for bacteria. Upon investigating a potential factor to increase TLR3, we focused on IRF2 (Fig. S3F–H). IRF2 is a known TLR3 transcriptional factor,^35^ with (i) a high binding score to the TLR3 promoter, (ii) a high Amadeus *p* value score of 7.8E-28 (Fig. S3F), and (iii) a high miR target binding score to IRF2 (by genie.weizmann.ac.il -15.33). To prove the direct effect of miR-122 on IRF2, we constructed a cellular luciferase assay system, in which, upon miR-122 transfection, human IRF2 was significantly reduced. Mutating the miR-122 seed sequence present in the 3′UTR of IRF2 abolished this miR-122 effect (Fig. S3I). Furthermore, injection of antagomir-122 into mice led to increased IRF2 expression (Fig. S3J and K). Thus, immune sensors for both viral and bacterial infections are regulated by miR-122, as shown for TLR2 and TLR4 (in other studies) and for TLR3 (in this study) (see the schematic description of IRF2 and TLR3 expression in Fig. S3L). In the transcriptomic investigation, TLR3 expression was significantly upregulated, with a 2.19-fold change (adjusted *p* = 0.013). IRF2 expression was also significantly upregulated, with a 1.81-fold change (adjusted *p* = 0.009).

### Macrophage activation in miR-122 KO mice

The observation of an enhanced macrophage liver population associated with enhanced fibrosis (Fig. 1A) in miR-122 KO mice, together with innate immune activation and TLR expression, led us to hypothesize that the liver macrophage population in miR-122 KO mice could be a cellular culprit for many of the observed phenotypes and changes in immune signaling. To determine the role of macrophages in miR-122 KO mice, we performed an *in vivo* macrophage depletion experiment (Fig. 4A). We repeatedly administered liposomal clodronate to young mice, which efficiently eliminated macrophages as well as significantly reduced liver fibrosis (Fig. 4B), indicating the importance of macrophages in fibrosis development. However, as shown in Fig. 4B, eliminating macrophages from 2-week-old mice and analyzing them at 6 weeks revealed that fibrosis remains very apparent, suggesting independent pathways linking fibrosis and macrophage activation in the liver. This was associated with a significant reduction in TLR4, as well as in IL-1β, IL-6, and IFNγ, but not in TLR2 and TLR3 (Fig. 4B). To determine the specificity of the effects of miR-122 on macrophages, we performed an *in vitro* macrophage activation experiment. A macrophage cell line was transfected with the miR-122 mimic or a negative control, in the presence or absence of the TLR4 inhibitor CLI-095, and then treated with low-dose LPS (5 ng/ml) and high-dose LPS (100 ng/ml), thereby mimicking TLR4 activation by bacterial infection. As shown in Fig. 4C, miR-122 specifically suppressed TLR4 as well as IL-6, IL-1β, CXCL1, and CXCL2, all of which are known to be involved in liver fibrosis (the suppression of AldoA indicates miR-122 activity, as Aldo A is a known target of miR-122). Furthermore, miR-122 suppressed the secretion of CXCL1 and IL-6 (Fig. 4D). The model we propose for this experiment is shown in Fig. 4E. To determine whether the liver macrophage/immune change in phenotype is associated with M1 or M2, we analyzed the scRNAseq data. Our findings reveal an increase in the M1 phenotype in the livers of miR-122 KO mice and a decrease in the M2 phenotype, as shown in Fig. 4F–H. These changes also include endothelial cells in the same direction.

**Fig. 4:**
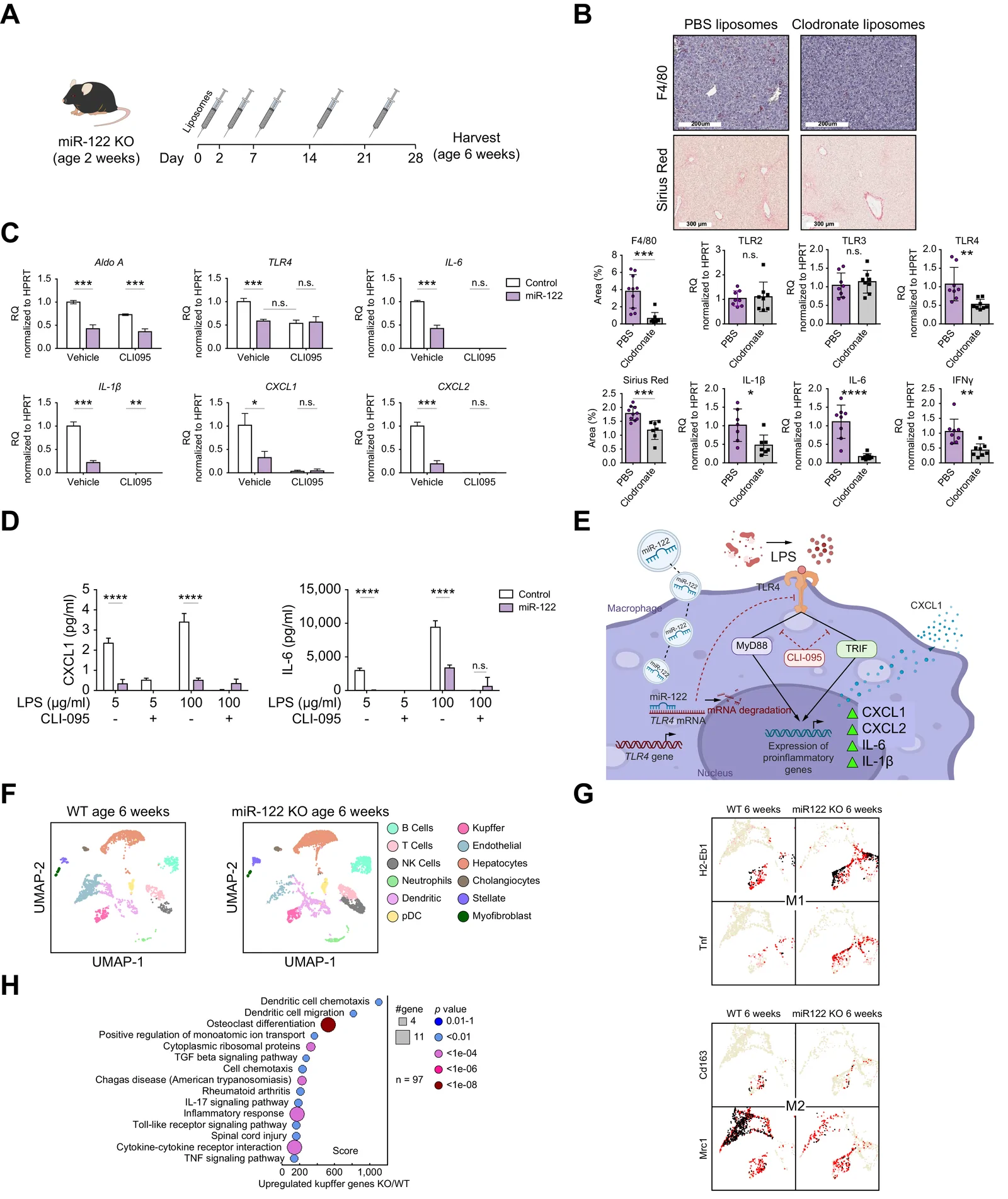
Immune phenotype in miR-122 KO mice is mediated by macrophages via TLR4. (A) Experimental design: miR-122 KO mice were injected i.p. with liposomal clodronate or liposomal PBS as a control beginning at 2 weeks of age on Days 0, 2, 7, 14, and 21 and harvested on Day 28 at 6 weeks of age. (B) Liver histology of miR-122 KO mice injected with liposomal clodronate or PBS control. Macrophages (F4/80) and fibrosis (Sirius Red). (C) Real-time PCR relative gene expression measured in the livers of miR-122 KO mice injected with liposomal clodronate or PBS control of downstream mediators of inflammation IL-1β, IL-6, and IFNγ, as well as TLR4, TLR2, and TLR3. Experimental design: RAW264.7 murine macrophage cells were transfected with the miR-122 mimic or the scr control. Forty-eight hours after transfection, cells were pretreated with the TLR4 inhibitor CLI-095 as a positive control or with a vehicle (10% DMSO). Cells were activated with LPS at two doses (5 or 100 ng/ml). Twenty-four hours after activation, medium and cells were collected for analysis. Real-time PCR relative gene expression measured in cells transfected with the miR-122 mimic compared with the scr control, treated with LPS (100 ng/ml). AldoA (positive control and a known target of miR-122), as well as TLR4 and the downstream mediators of inflammation IL-6, IL-1β, CXCL1, and CXCL2. (D) Secretion of the proinflammatory markers CXCL1 and IL-6 into the medium was measured by ELISA. (E) Scheme of the proposed function of miR-122 on TLR4 signaling in macrophages. (F) ScRNAseq data generated from cells isolated from the livers of miR-122 KO mice and WT controls at 6 weeks of age, sorted into clusters. (G) ScRNAseq analysis of the livers of miR-122 KO mice and WT controls at 6 weeks of age, indicating the clusters of cells expressing M1 and M2 markers. (H) Pathway analysis for KO Kupffer *vs*. WT Kupffer from scRNAseq samples. DEGs are selected with adjusted *p* value <0.02 and applied to over-representation analysis using the following libraries: WikiPathways_2024_Mouse, KEGG_2019_Mouse, GO_Biological_Process_2025. ∗*p* <0.05, ∗∗*p* <0.01, ∗∗∗*p* <0.001, ∗∗∗∗*p* <0.0001. DEG, differentially expressed gene; KO, knockout; LPS, lipopolysaccharide; RNAseq, RNA sequencing; scRNAseq, single-cell RNA sequencing; TLR, Toll-like receptor; WT, wild type.

### Role of CD4 and CD8 cells in the miR-122 KO mice

To determine whether the inflammation and fibrosis observed in the miR-122 KO mice depend on CD4 and CD8 cells, we treated the mice with a combination of anti-CD4 and anti-CD8 antibodies to deplete both cell types (Fig. S6A). This treatment significantly reduced both T-cell subsets, as shown by CD3 staining (Fig. S6B). As shown in Fig. S6C, CD8 cells were significantly depleted, as confirmed by PCR analysis, corroborating efficient treatment. The depletion of both CD4 and CD8 cells did not affect fibrosis or inflammatory signaling pathways in miR-122 KO mice. The results from Sirius Red staining indicate no significant difference after depleting both CD4 and CD8 cells. In addition, the combination treatment showed no effect (Fig. S6B). The analysis of αSMA message further confirms that depleting CD4 and CD8 cells had no effect on fibrosis (Fig. S6C).

### Liver autoimmunity

The hepatic infiltration of immune cells (Fig. 1, Fig. 2, Fig. 3, Fig. 4) and enhanced TLR expression led us to speculate that chronic autoimmune hepatitis could develop in these miR-122 KO mice. Furthermore, miR-122 KO mice exhibit ductular reaction.^36^ To identify the pivotal pathways relevant to the immune privilege of the liver microenvironment, we compared the RNAseq data of patients with AIH with those of miR-122 KO mice, owing to the similarities we observed (Fig. 5A). The RNAseq data of 13 patients with AIH were scored using the modified Hepatitis Activity Index (mHAI) system,^37^ which enabled us to categorize the patients into two groups: the low-mHAI (<6) and high-mHAI (>9) score patients (Fig. 5B,C). The high-mHAI patients had an enhanced NF-κB signaling pathway as well as an enhanced TLR pathway (Fig. 5D). Liver miR-122 levels negatively correlated with the mHAI score (Fig. 5E). Following this human and mouse analysis, we learned that TLR signaling is predominant in human AIH (Fig. 5D). In the RNA expression data of the 2-, 3-, and 6-week-old miR-122 KO mice, we observed, at most time points, a significant increase in TLR2, TLR3, and TLR4 (Fig. 5F). MiR-122, although expressed in hepatocytes, is secreted and also affects other cell types, such as Kupffer cells and macrophages, possibly explaining the overall increase in the expression of these three TLRs in miR-122 KO mice. TLR4 is also associated with liver fibrosis^38^ and is a target of miR-122.^34^ To determine the functionality of enhanced TLR4 expression, we treated the miR-122 KO mice with a mixture of oral antibiotics, assuming that gut microbiome signaling occurs through hepatic TLR4, as previously reported^39^ (Fig. S7A and B). This antibiotic treatment reduced liver macrophages (although not significantly), liver fibrosis (Fig. S7C), and hepatic expression of TNFα, IL-1β, IFNγ, IL-12b (IL-12a and IL-6 were reduced, but not significantly), and TLR4 (Fig. S7D). This suggests that a mechanism of enhanced liver fibrosis is associated with the gut microbiome–TLR4 liver axis. The inflammatory state of these mice was also evidenced by increased serum amyloid A1 (SAA1) and SAA2 RNA levels at 2, 3, and 6 weeks (Fig. S8A and B). At the protein level, the SAA measured in the serum of miR-122 KO mice did not reach statistical difference (Fig. S8C), but the SAA in the blood of miR-122 KO mice was increased. This increase was mainly observed in hepatocytes, as indicated by scRNAseq data (Fig. S8D).

**Fig. 5:**
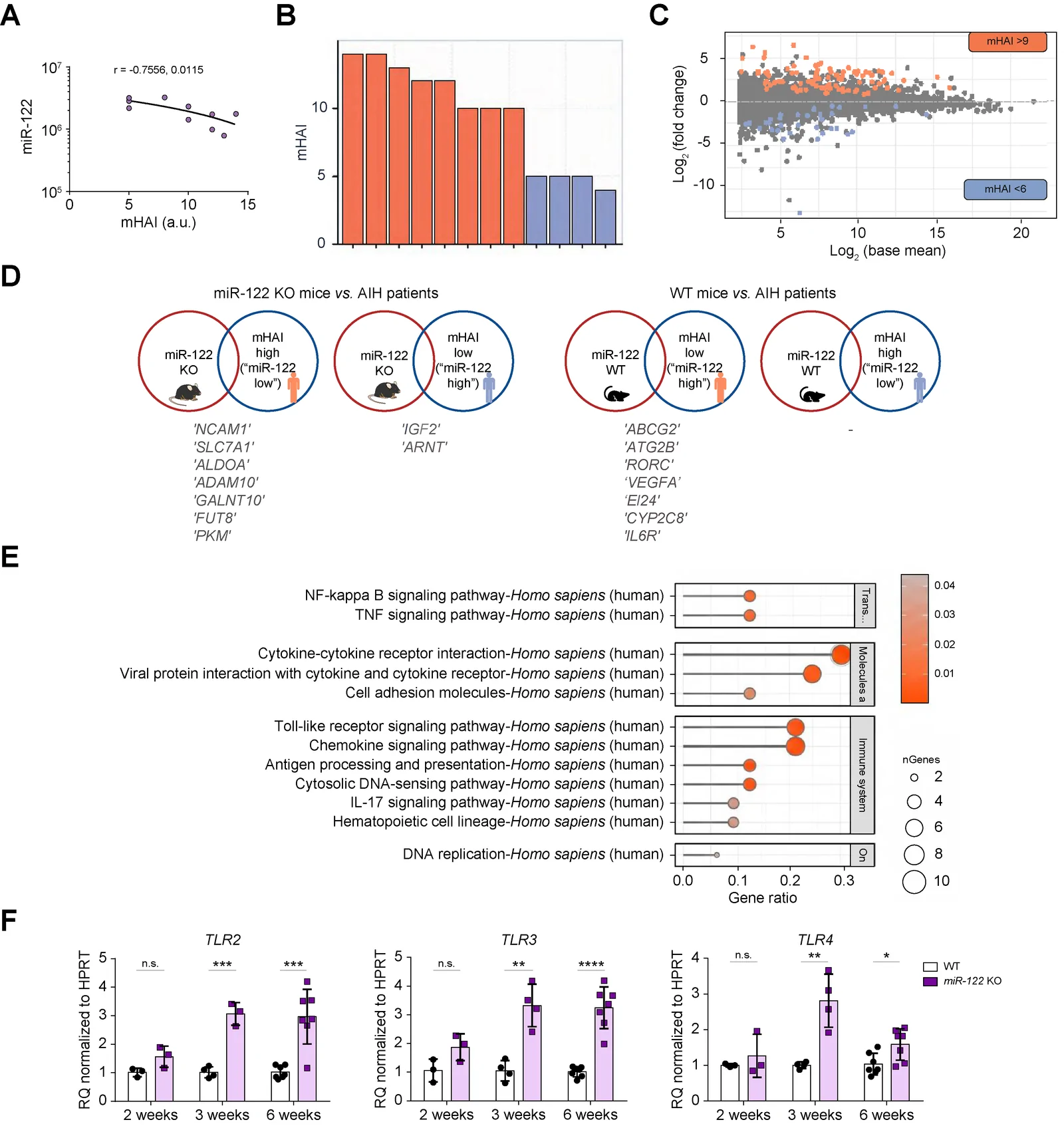
Low expression of miR-122 in patients with AIH induces TLR signaling upregulation. (A) Spearman correlation between miR-122 expression in liver samples and mHAI score in patients with AIH. (B) Patients with AIH grouped into high-mHAI (orange) and low-mHAI (blue) individuals. (C) RNAseq gene expression analysis of liver samples from patients with AIH: MA plot of differential gene expression between high- and low-mHAI patients with AIH. (D) Gene set enrichment analysis indicating pathways upregulated in the livers of high-mHAI patients with AIH. (E) Overlap between overexpressed genes in bulk mRNA-sequencing data from liver samples of high- and low-mHAI patient groups and bulk mRNA-sequencing data from the livers of miR-122 KO mice and WT controls at 6 weeks of age. (F) Real-time PCR confirmation of expression of TLR2, TLR3, and TLR4, measured in the livers of miR-122 KO mice and WT controls at 2, 3, and 6 weeks of age. ∗*p* <0.05, ∗∗*p* <0.01, ∗∗∗*p* <0.001, ∗∗∗∗*p* <0.0001. AIH, autoimmune hepatitis; KO, knockout; mHAI, modified Hepatitis Activity Index; RNAseq, RNA sequencing; TLR, Toll-like receptor; WT, wild type.

### Liver fibrosis is miR-122 dependent

Liver fibrosis is associated with chronic liver diseases; however, both are mechanistically not fully deciphered and have no effective therapeutic treatment.^40^ We investigated the effect of miR-122 on liver fibrosis because of the observation that in the miR-122 KO model, fibrosis co-occurs with inflammation rather than occurring in a secondary manner. The phenotypic change of enhanced liver fibrosis in these miR-122 KO mice was apparent shortly after birth (Fig. 1A), once the gut microbiome was established. This led us to further investigate the role of miR-122 in liver fibrosis using RNAseq and real-time PCR analyses (Fig. 6A,B). We observed increased αSMA expression in the livers of these miR-122 KO mice already at an early age (Fig. 6B). In addition, upon examining the RNAseq data of the miR-122 KO mice, we observed an increase in the expression of collagen genes, which is most apparent at Week 6 of age (Fig. 6A). TGFβR2, the dominant TGFβR, is a target of miR-122^41^ and is also increased, as depicted in a volcano plot and confirmed by RT-PCR (Fig. 6A,B). This increase in collagen genes is further supported by an analysis of ligand–receptor interactions derived from scRNAseq data^42^ (Fig. 6C,D). The robustness of the increase in collagen expression in interaction with the receptor is most apparent in these miR-122 KO mice. To further assess the presence of pro-fibrotic effectors, we investigated two chemokine receptors, CCR1 and CCR5,[43], [44] which are associated with liver fibrosis and are both increased in miR-122 KO mice (Fig. 6B). In these KO mice, the target of these collagens, integrin α2β1, is enhanced (Fig. 6D). These observations fit with the overall effect of miR-122 in suppressing liver fibrosis, which probably occurs through a direct effect on several pro-fibrotic mediators, in which miR-122 suppresses their expression and thus modifies the inflammatory pro-fibrotic stimuli.

**Fig. 6:**
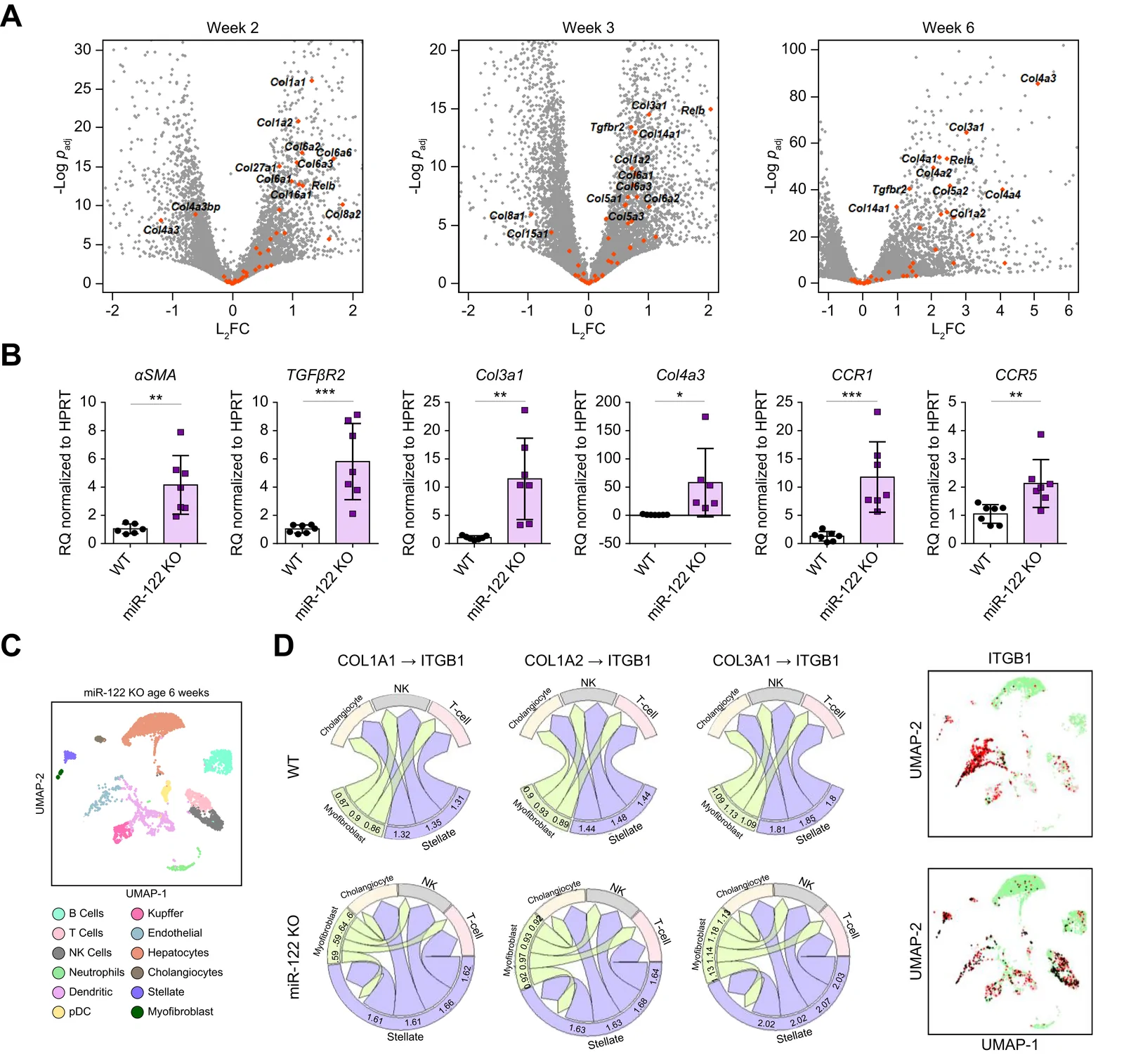
Increased fibrosis in miR-122 KO mice. (A) Volcano plot of differentially expressed genes in miR-122 KO mice compared with WT controls, at 2, 3, and 6 weeks of age. Genes associated with fibrosis are indicated in orange. (B) Real-time PCR confirmation of differentially expressed fibrosis-related genes at 6 weeks of age. (C) ScRNAseq data sorted into clusters with legend. (D) CellPhoneDB collagen (COL1A1, COL1A2, and COL3A1) and integrin α2β1 (ITGB1) interactions associated with fibrosis from scRNAseq data: interaction analysis of myofibroblasts (green) and stellate cells (purple) with cholangyocytes (yellow), NK cells (gray), and T cells (soft pink). Interaction expression scores are indicated on the plot and represented by the width of the ribbon. ∗*p* <0.05, ∗∗*p* <0.01, ∗∗∗*p* <0.001. KO, knockout; scRNAseq, single-cell RNA sequencing; WT, wild type.

Osteopontin (SPP1) is increased in many chronic liver diseases^45^ and is associated with liver fibrosis. Furthermore, SPP1 is associated with clinical conditions identified in a mouse model of inflammation and fibrosis. SPP1, associated with ductular reaction and fibrosis, was significantly increased in the miR-122 KO mice (Fig. S9A). This increase was apparent in 2-, 3-, and 6-week-old miR-122 KO mice, as shown by RNAseq (Fig. S9B) and confirmed by PCR in the 6-week-old mice (Fig. S9C). This increase in SPP1 transcripts levels is primarily observed in the hepatocyte population (Fig. S9D).

## Discussion

The regulation of liver tolerance, inflammation, and fibrosis is central to many important clinical pathologies, both hepatic and systemic. These include liver cancer, metastasis, and infections caused by viruses, bacteria, and parasitic pathogens with hepatotropism, such as hepatitis viruses, which thrive in the liver. Autoimmune liver diseases and liver transplantation are managed with immune suppression drugs, underscoring the pivotal role of liver immune regulation. However, our understanding of the regulation of the liver immune microenvironment is limited. This gap hampers the development of therapeutics for various diseases. Previous studies and our preliminary clinical observations indicate that autoimmune liver diseases are associated with low liver miR-122 levels. This led us to propose that low miR-122 levels in the liver result in a breach in liver immune tolerance. MiR-122 is a liver-specific microRNA secreted by hepatocytes in exosomes, enabling its delivery to other liver cell types, including macrophages, endothelial cells, stellate cells, and immune cells, as well as to other organs in the body that are distant from the liver, through the circulation, as we reported for the kidney, adipose tissue, and muscle.^8^ This microRNA is unique because it is produced in very high levels in each hepatocyte (>100,000 copies/hepatocyte). To understand the role of miR-122, we used several techniques, including administration of miR-122 mimics and antagomir-122, and the generation of miR-122 KO mice. In our study, the excised genomic region was small (59 nt), unlike in previous miR-122 KO mice, which had a significantly larger genomic region deleted, possibly causing off-target effects. Moreover, unlike earlier clinical observations of miR-122 KO mice,[15], [23] which assessed the development of liver inflammation, steatosis, and HCC,[12], [13] we were interested in determining the alterations occurring in the liver at an early age, as we assumed that changes in this early phase would have major significance. To recapitulate the previous findings reported in miR-122 KO mice, our observations align with these reports. We confirmed that our miR-122 KO mice, which suffer from fibrosis and inflammation at an early age, almost all develop primary liver cancers at a later stage. We believe that this phenomenon is significant, potentially demonstrating that reduced miR-122 levels in human liver inflammatory states contribute to early liver fibrosis and inflammation, which culminate after several years in primary liver cancer. The reported observations that miR-122 is a direct tumor suppressor, increases p53, and is a negative regulator of fibrosis and inflammation could position miR-122 at the forefront of future therapeutic targets for chronic inflammatory liver diseases and liver cancers.

The liver is continually exposed to the gut microbiome, which includes bacteria, fungi, and viruses. Our research shows that reducing the gut bacterial load in miR-122 KO mice suppresses liver inflammation and attenuates macrophage activation (Fig. S7). This suggests that miR-122 regulates the pro-inflammatory part of the gut–liver axis. This regulation by miR-122 of the pro-inflammatory axis of the gut–liver effect may occur through negative control of TLR2, TLR3, and TLR4. This is potentially significant in attenuating the activation of liver inflammation by the gut microbiome, which flushes through the portal vein into the liver.

By using early-age mice and multiple approaches, we identified the multilayered immune effects of miR-122. The reduction of this microRNA enhances innate immune responses in the liver. The miR-122 mimic reversed the immune phenotype, thereby enabling tolerance. MiR-122 KO mice exhibited an increased innate immune response in the liver. Eradication of miR-122 increased the homing effects of chemokines and chemokine receptors on immune cells to migrate to the liver. These observations were associated with the simultaneous initiation of several pro-fibrotic pathways, leading to very early fibrosis in the miR-122 KO mice, beginning at weeks 2–3 of life. Liver macrophages are central to these inflammatory phenotypes, as demonstrated by our *in vitro* and *in vivo* studies. Recent reports have highlighted some interesting potential mediators of liver fibrosis in miR-122 KO mice: Bmp8 and Ihh (Indian hedgehog). They are both significantly upregulated in the miR-122 KO liver and have been bioinformatically defined as direct targets of miR-122 (Table S4). Bmp8 has been shown to promote a pro-inflammatory phenotype in hepatic stellate cells by signaling through both the SMAD2/3 and SMAD1/5/9 branches of the TGFβ/BMP pathway and may also contribute to the fibrotic phenotype of the miR-122 KO liver. Ihh encodes a ligand of the Hedgehog signaling, whose activation in cholangiocytes and liver stromal cells induces inflammatory and fibrogenic wound-healing responses. Recently, it was shown that macrophage β–catenin–Ihh axis induces stellate cell activation and fibrosis. Periostin promotes Ihh expression, which, in turn, promotes hepatic stellate cell activation, also contributing to the fibrotic phenotype of miR-122 KO mice. Altogether, it seems that miR-122 is a central regulator of liver fibrosis. Currently, with no approved drug to prevent or reduce liver fibrosis, miR-122 regulation could be proposed as a target to modulate liver fibrosis (Fig. 7).

**Fig. 7:**
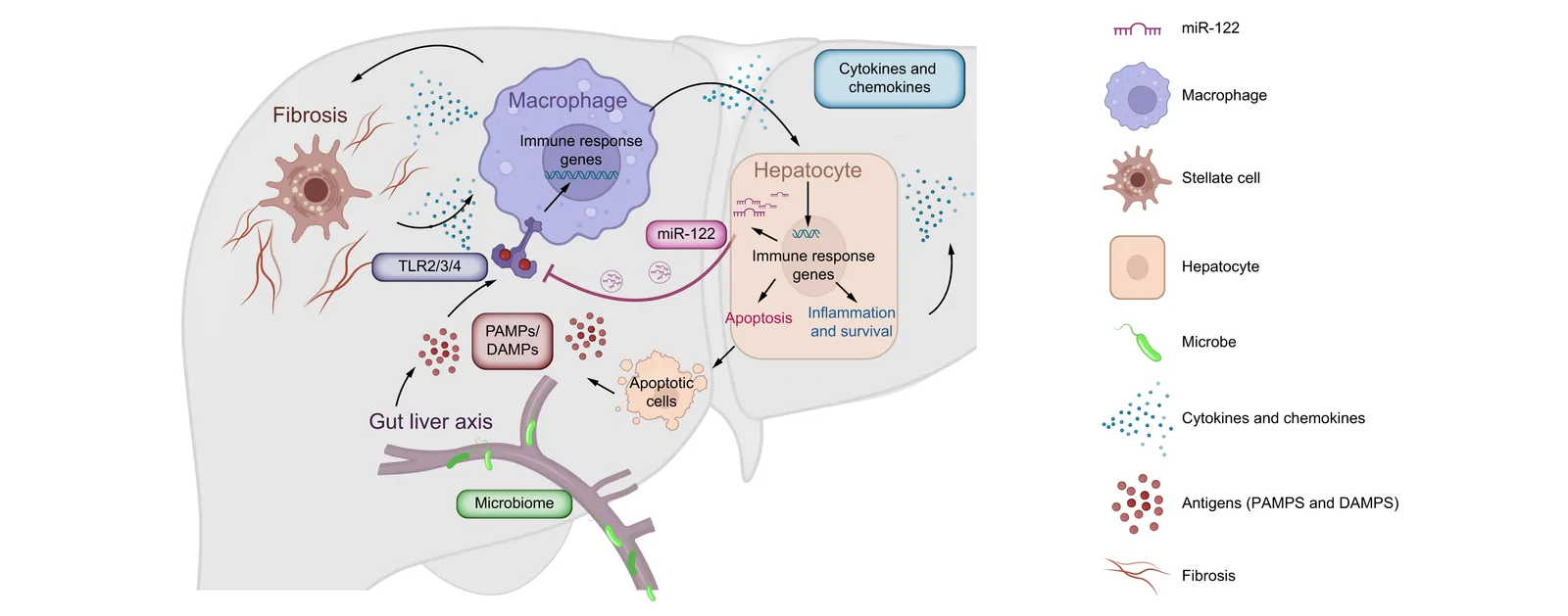
Graphical representation of the role of miR-122 in the cellular crosstalk of liver immune regulation.

Our current understanding, based on these results, is that reduced levels of miR-122 in the liver during chronic liver inflammatory states, as many previous studies have shown, lead to liver inflammation and fibrosis through independent pathways. This could explain the co-occurrence of both pathologies in many chronic liver clinical conditions.

## Abbreviations

αSMA, alpha-smooth muscle actin; AIH, autoimmune hepatitis; CCR1, C–C chemokine receptor type 1; CCR5, C–C chemokine receptor type 5; CD4, cluster of differentiation 4; CD8, cluster of differentiation 8; CTLA-4, cytotoxic T-lymphocyte-associated protein 4; CXCL1, C-X-C motif chemokine ligand 1; CXCL12, C-X-C motif chemokine ligand 12; CXCL2: C-X-C motif chemokine ligand 2; CXCR4: C-X-C motif chemokine receptor 4; GSEA, gene set enrichment analysis; HCC, hepatocellular carcinoma; HLA, human leukocyte antigen; IFNγ, interferon gamma; IRF2, interferon regulatory factor 2; KEGG, Kyoto Encyclopedia of Genes and Genomes; KO knockout; LAG-3, lymphocyte activation gene-3; LPS, lipopolysaccharide; mHAI, modified Hepatitis Activity Index; miR-122, microRNA-122898/21; PD-1, programmed death-1; pSTAT3, phosphorylated signal transducer and activator of transcription 3; RNAseq, RNA sequencing; RT-PCR, reverse transcription PCR; SAA, serum amyloid A; scRNAseq, single-cell RNA sequencing; SPP1, secreted phosphoprotein 1 (also known as osteopontin); TCR, T-cell receptor; TGFR, transforming growth factor receptor; TGFβR2, transforming growth factor-beta receptor 2; TIM-3, T-cell immunoglobulin and mucin domain-containing protein 3; TLR, Toll-like receptor; TNFα, tumor necrosis factor-alpha; WT, wild type.

## Authors’ contributions

Conceptualization: MG, ZS, SRJ, MH, HG, EG. Methodology: MG, SL, EK, ZS, JB, TJ, LA, CK, MO, OG, NA, RA, YN, SE, HB, JM, EZK, NR, AM, DY, ASY, AS, MR, OA, DSG, AP, JA, JR, SH, NG, CSc, JHer, AC,RS, AH, AI, DH, JHet, SRJ, MH, HG, EG. Investigation: all authors. Visualization: MG, SL, EK, ZS, NA, RA. Funding acquisition: MH, EG. Project administration: None. Supervision: MH, HG, EG. Writing—original draft: MG, EG. Writing—review & editing: MH, DSG, HG, EG.

## Data availability

All data relating to the paper are deposited in the GEO public database (accession number GSE278824).

## Financial support

This work was supported by the National Institutes of Health grant CA197081-02 (to EG); MOST–DKFZ grant 4037 (to EG); the ISF collaboration with Canada grant (2473/2017) (to EG); the personal ISF grant (486/2017) (to EG); the ISF IPMP grant (3751/21) (to EG); the Deutsche Forschungsgemeinschaft, Bonn, Germany, grant (SFB841) (to EG); the ERC Advanced Grant No. 786575—RxmiRcanceR, including the PanRORa POC 101188783 (to EG); the HFSP RGP0011/2022 grant (to EG); the Kron, Raskin, and Einstein grant (A-2021-646) (to EG); the Robert Benson Foundations grant (to EG); and the Kamea Scientific Foundation of the Israeli Government (to DSG). JH and EG were also supported by the DFG grant 544225277

## Conflicts of interest

The authors declare that they have no competing interests.

Please refer to the accompanying ICMJE disclosure forms for further details.
