# Supplementary material for: MiR-122 regulates liver tolerance
Source: JHEP Rep. 2026 Aug 29;8(12):102020. doi: 10.1016/j.jhepr.2026.102020 (PMC13770681; doi:10.1016/j.jhepr.2026.102020)

MiR-122 regulates liver tolerance

Maytal Gefen, Shanny Layani, Emma Klahr, Zohar Shemuelian, Johannes Brandi, Thomas Jacobs, Christiane Steeg, Lorenz Adlung, Christoph Kilian, Merav Ordan, Oren Gordon, Nathalie Abudi, Rinat Abramovitch, Yuval Nevo, Sharona Elgavish, Hadar Benyamini, Jonathan Monin, Elina Zorde Khvalevsky, Nofar Rosenberg, Aurelia Markezana, Dayana Yaish, Adi S. Yehezkel, Alina Simerzin, Mila Rivkin, Osher Amran, Daniel S. Goldenberg, Amnon Peled, Jonathan Axelrod, Jacob Rachmilewitz, Samuel Huber, Nicola Gagliani, Christoph Schramm, Sören Weidemann, Johannes Herkel, Antonella Carambia, Rifaat Safadi, Abed Halila, Asraf Imam, Daniela Heide, Jenny Hetzer, Stefan Rose-John, Mathias Heikenwälder, Hilla Giladi, Eithan Galun

Table of contents

Supplementary materials and methods……………………………………………..…..2

Supplementary tables…………………………………………………………...…..17

Supplementary figure legends…………………………………………..………....22

Supplementary figures……………………………………………………………….27

Supplementary materials and methods

Mice

miR-122 knockout (KO) mice (C57BL/6miR-122em2Hhtg) were generated in collaboration with Prof. Dr. Nicola Gagliani and Prof. Dr. Samuel Huber at the University Medical Center Hamburg-Eppendorf, Hamburg, Germany. The genomic region of miR-122 (chr18:65,381,932-65,381,997) was targeted with 2 sgRNA guide sequences (sgRNA1:AACAGACGTCAAGTCACGCGTGG, sgRNA2:AGTGTGATAATGGCGTTTGATGG) generating a 60bp deletion. All mice were screened by genotyping the miR-122 genomic region by PCR analysis (for primer sequences, see Table S1). Two separate colonies generated by separate founders were maintained for experimentation. Male mice were used in all the experiments. Mice were maintained under specific pathogen-free (SPF) conditions in an animal facility with a temperature of approximately 23°C in a 12-hour light-dark cycle and received sterile commercial rodent chow and water ad libitum unless otherwise indicated. Maintenance of mice and all experimental procedures were performed by the Institutional Animal Care and Use Committee approved animal treatment protocols (license number OPRR-A01– 5011). All animal experiments were performed after a stringent ethical consideration and approval by the Hebrew University Of Jerusalem, Israel. The experiments were regulated by veterinarian specialists.

Alb-iGP_Smarta mice[51] were bred and maintained under specific pathogen-free conditions at the animal facility of the University Medical Centre Hamburg-Eppendorf (Hamburg, Germany). All animals received humane care, and clinical and behavioral humane endpoints were applied to minimize any harm. All animal experiments were conducted in accordance with the Basel Declaration, the European Directive 2010/63/EU, and German federal law.

Experiments were approved by the relevant review board and authorities of the State of Hamburg, Germany.

In Vivo experiments (procedures in live mice)

Magnetic Resonance Imaging (MRI) monitoring

MRI experiments were performed at the Wohl Institute for Translational Medicine at Hadassah Hebrew University Medical Center. MRI images were acquired on a 7T 24 cm bore, cryogen-free MR scanner based on the proprietary dry magnet technology (MR Solutions, Guildford, UK) using 38-mm inner diameter, 70 mm length mouse whole body volume coil. For MRI acquisition, mice were anesthetized with isoflurane vaporized with O_2. Isoflurane is used at 3.0% for induction and at 1.0 - 2.0% for maintenance. The mice were positioned on a heated bed, which allowed for continuous anesthesia and breathing rate monitoring throughout the entire scan period. Coronal T1-weighted (T1W) spin-echo images were acquired for anatomical segmentation purposes (repetition time = 1100 milliseconds, echo time = 11 milliseconds, FOV= 60mm, in-plane resolution = 230 μm, slice thickness = 1 mm). Coronal and axial T2-weighted (T2W) spin-echo images were also acquired for liver pathologies assessment (repetition time = 2500 ms, echo time = 45 ms, in-plane resolution = 230 μm, slice thickness = 1 mm).

Inhibition of miR-122 in a model of acute inflammation

For inhibition of miR-122, 7 to 8 weeks old, male, C57BL/6 mice, purchased from Harlan Laboratories (Jerusalem, Israel), were hydrodynamic tail vain injected (HTVI) with antagomiR-122 or antagomiR-control (7 µg/mouse in 1.5 mL saline, Sigma-Aldrich, St Louis, MO).

antagomiR-122	5'-mAsmCsmAmAmAmCmAmCmCmAmUmUmGmUmCmAmCmAmCmUsmCsmCsmAs-Chol-3'

antagomiR-control	5'-mCsmAsmCmCmAmCmAmUmAmCmCmGmCmAsmCs mGsmGs-Chol-3'

Twenty-four hours post HTVI, the mice were injected subcutaneously (SC) with LPS (1 µg/kg, L2630, Sigma-Aldrich St Louis, MO) and 5 hours later, the mice were sacrificed and their livers, were flash frozen in liquid nitrogen or in optimum cutting temperature (OCT) embedded frozen blocks, for further RNA and histologic analysis.

Macrophage depletion

For the depletion of macrophages, mice were injected with 200µl Clodronate Liposomes (Liposoma, The Netherlands), or control liposomes (PBS) as control (Liposoma, The Netherlands), by intraperitoneal (IP) injection. Starting at the age of 2 weeks, mice were injected at days 0, 2, 7, 14, 21 and then livers were harvested on day 28 of the experiment, at the age of 6 weeks.

Bacteremia model

Methicillin-resistant Staphylococcus aureus (MRSA) bacteremia model: The USA300 S. aureus strain SAP231 (derived from the community-acquired MRSA strain NRS384, which was isolated from an outbreak in the Mississippi prison system) was used and was generated as described previously[52]. SAP231 was streaked on tryptic soy agar (TSA) plates (tryptic soy broth [TSB] plus 1.5% Bacto agar [BD Biosciences]) and grown overnight at 37°C in a bacterial incubator. Two or three colonies were picked and cultured in TSB at 37°C in a shaking incubator (LAB-LINE) at 240 rpm overnight (18 h), followed by a 1:50 subculture at 37°C for 2 h to obtain mid-logarithmic-phase bacteria. The bacteria were pelleted, washed two times, and resuspended in sterile phosphate-buffered saline (PBS) at the indicated concentrations for each model below. The absorbance at 600 nm (A600) was measured to estimate the number of CFU, which was verified after overnight culture on TSA plates. All animal experiments were approved by the Hebrew University of Jerusalem Animal Care and

Use Committee. The S. aureus bacteremia model was modified from a previously described protocol[53]. Briefly, WT or miR1-22 KO 6-week-old male C57BL/6 mice inoculated IP with 5 x 10^7 CFUs of SAP231 in a 100-mL volume of PBS using a 29-gauge insulin syringe. Clinical score was determined by measuring weight and body temperature. To measure bacterial burden, enumeration of CFU was performed with overnight plate cultures of homogenized tissue (TissueLyser LT; QIAGEN). Livers were harvested 18 hours following infection. The CFU were counted after overnight culture with serial dilutions on TSA plates.

Malaria infection

For mouse malaria parasitemia model, miR-122 KO mice breeding pairs were sent to The Bernhard Nocht Institute for Tropical Medicine (BNITM), Hamburg, Germany. There they were crossed and bred with C57BL/6J mice at the animal facility of the BNITM, Hamburg, Germany in individual ventilated cages (IVC) with food and water ad libidum, to establish a miR-122 KO colony. All animal experiments were performed in accordance with German law and registered with the “Behörde für Gesundheit und Verbraucherschutz (BGV)”. Stabilates with erythrocytes from Plasmodium berghei C57BL/6 mice were stored in 0.9% NaCl, 4.6% sorbitol and 35% glycerol in liquid nitrogen. In order to increase viability of the parasites, they were passaged once. To this end, parasites were thawed, diluted in 0.9% NaCL and i.p. injected into C57BL/6J mice. Fresh blood of passage mice, taken 5 days post infection, was used to transfer 1x 105 infected red blood cells/200 µl PBS i.p. into mice used for experiments.

Cerebral symptoms were monitored daily and scored according to the following scheme: 0 = no symptoms; 1 = decreased activity, deceleration; 2 = ataxia; 3 = weight loss≥ 20% or convulsions, strong ataxia; 4 = coma; 5 = death. If a score of ≥3 was reached, mice were euthanized in order to avoid unnecessary suffering.

Microbiome depletion

For microbiome depletion at the Hebrew University animal farm, breeding pairs were treated with vancomycin (500 mg/L), imipenem (500 mg/L) and neomycin (1 g/L) in drinking water as antibiotic cocktail, and the antibiotics-containing water was changed twice a week. Pups, born to parents treated with antibiotics, were treated continuously, also after weaning with the same antibiotics, up to the age of 6 weeks when the mice were harvested. Microbiome bacterial load was determined by measuring fecal DNA (ZymoBiomics DNA miniprep kit, D4300 Xymo, USA)

T cell depletion

For the depletion of T cells, mice were injected with 200ug anti CD4 (clone GK1.5, cat#BE0003-1, Bioxcell) and 200µg anti CD8 (clone 2.43, cat#BE0061, Bioxcell) or 400µg IgG isotype control (clone LTF-2, cat#BE0090, Bioxcell), diluted as recommended by the manufacturer in InVivoPure pH 7.0 dilution buffer (cat#IP0070, Bioxcell) or in InVivoPure pH 6.5 dilution buffer (cat# IP0065, Bioxcell). Mice were injected IP at the age of 2 weeks. Following the first injection, mice were injected with 100µg anti CD4 and 100µg anti CD8, or 200µg IgG as control, by IP injection at days 7, 14, 21 and then livers were harvested on day 28 of the experiment, at the age of 6 weeks.

Mice tissue handling

For all *in vivo* mice experiments, livers were extracted and weighed, and liver tumors with a diameter >2 mm were counted and recorded. Liver specimens were either fixed in 4% buffered formalin or snap-frozen in liquid nitrogen for further analysis. Serum was obtained from whole blood and either analyzed immediately or stored at -80°C for further analysis.

Mice blood chemistry

Mice liver enzymes were measured by COBAS 600 using the appropriate kits for detection of ALP (Alkaline Phosphatase acc. to IFCC Gen.2, Roche), SGPT (Alanine Aminotransferase acc. to IFCC without pyridoxal phosphate activation, Roche) and SGOT (Aspartate

Aminotransferase acc. to IFCC without pyridoxal phosphate activation, Roche), LDH (Lactate Dehydrogenase optimized, Roche). Hematology was measured by XS-1000i ™ Automated Hematology Analyzer (Sysmex). Determination of different white blood cell types was performed manually by a qualified veterinarian.

<u>Mice histopathological analysis</u>

Liver samples were fixed in 4% paraformaldehyde and paraffin-embedded (FFPE). Briefly, tissue was cut into 5µm sections from FFPE and stained with Hematoxylin/Eosin or Sirus red (Direct Red 80, Sigma-Aldrich, Rehovot, Israel) or IHC primary antibodies (Table S2). Epitope retrieval was obtained by using citrate buffer or Pronase (1mg/ml, P8038, Sigma-Aldrich, St Louis, MO). For IHC staining, we used a conjugated horseradish peroxidase secondary Ab (anti-mouse and -rabbit [Envision; Dako, Denmark] and antirat [Histifine; Nichirei, Osaka, Japan]) for 1 hour. All slides were counterstained with Mayer's hematoxylin and mounted. Cell death/apoptosis analysis was performed by TUNEL assay according to the manufacturer's protocol (ab206386, Abcam, Cambridge, United Kingdom). Negative and positive controls were included. For quantifications of staining, slides were scanned using an SCN400 slide scanner (Leica, Germany) and analyzed using the ImageJ software. Images of stained sections were quantified as the percentage of positively stained area per high power field or as the number of positively stained cells per field in 20µm or 40µm magnification, in 8 to 20 pictures per sample, using ImageJ, RRID: SCR_003070.

<u>Flow cytometry</u>

Isolation of hepatic immune cells: cells were isolated from liver and spleen of WT and miR-122 KO animals 6 weeks of age. Briefly, the animals were directly perfused with PBS with 2% FCS through the vena cava. A part of the liver was removed for histology, the remaining liver was cut into small pieces and digested in Liver digest Medium (Cat# 17703034, ThermoFisher Scientific/ Gibco, Waltham, Massachusetts) for 30 minutes at 37°C. Following

this incubation step, the liver pieces were forced through a 100µm cell strainer and washed with PBS with 2% FCS. The resulting suspension was centrifuged at 40 x g, 4°C for 5 minutes and the supernatant was collected and spun again at 400 x g, 4°C, 5 min. The cell pellet was resuspended in 40% Percoll (Cytiva, Sweden) and centrifuged at 650 x g for 20 min. at room temperature. The supernatant was discarded, and the pellet washed with PBS with 2% FCS. Afterwards, the pellet was resuspended in Ammonium-Chloride-Potassium (ACK) lysis buffer and incubated for 5 minutes. Lysis was stopped by the addition of PBS with 2% FCS. Cells washed in PBS with 2% FCS and then counted.

Flow cytometry analysis: A detailed description of the staining protocol was previously reported[54]. In brief, cells were adjusted for 1.5 x 10^6 cells and incubated with Fc-block (TruStain FcX™ PLUS Block (Cat#156604, BioLegend, San Diego, California). The surface staining was performed in Brilliant stain buffer (Cat# 563794, BD Bioscience, Franklin Lakes, New Jersey) with antibodies as detailed in Table S3. For intracellular staining, cells were fixated, permeabilized and stained using the eBioscience FoxP3/ Transcription Factor Staining Buffer Set (Cat#00-5523-00, ThermoFisher). For intracellular staining, antibodies detailed in Table S3 were used. For compensation, Ultra Comp eBeads (Cat 01-2222-41, ThermoFisher), were used and stained simultaneously with their respective antibody. After staining, cells were washed with PBS with 2% FCS. Cells were then fixated with 4% PFA and washed again with PBS with 2% FCS and kept on 4°C until they were acquired on a 5L Aurora (Cytek Biosciences, Bethesda, Maryland).

Cell culture

HEK293 Human embryonic kidney cells, and RAW264.7 mouse macrophage cells, were cultured in DMEM supplemented with 10% fetal calf serum (FCS), 1% penicillin/streptomycin (Thermo Scientific, Waltham, MA, USA). All cells were cultured at 37°C in a humidified atmosphere containing 5% CO_2.

Transfection procedures

Transfection of plasmids was performed with Lipofectamine 2000 transfection reagent (Cat#11668027; Invitrogen, Carlsbad, CA, USA) according to the manufacturer's protocol and using serum-free medium (Opti-MEM; Cat#31985070; Thermo Scientific). For luciferase assays, Hek293 cells in 24 well plates were transfected (20nM). Transfection of mimic miR-122 and scrambled control was performed with HiPerFect transfection reagent (Cat # 301705, QIAGEN) according to the manufacturer's protocol. RAW264.7 cells in 24 wells plates were transfected (50nM).

Sequence mimic miR-122:

sense	/5Phos/rUrGrGrArGrUrGrUrGrArCrArArUrGrGrUrGrUmUmUrG
antisense	mAmArCmArCmCrAmUrUmGrUmCrAmCrAmCrUmCrCA

Sequence mimic-miR, random scra:

sense	/5Phos/rGrCrGrArCrUrArUrArCrGrCrGrCrArArUrArUmGmGrU
antisense	mCmArUmArUmUrGmCrGmCrGmUrAmUrAmGrUmCrGC

Construction of miR-122 reporter plasmids and Luciferase activity assay

A fragment from 3'UTR of IRF2 gene containing a putative miR-122 target, was synthesized by PCR amplification and was then cloned in the pmirGLO vector between the SacI–SalI sites. The mutated miR-122 binding site in the 3'-UTR of the human IRF2, was created using a mutated fragment by two PCR steps. For the first step, wt IRF2-3'UTR was used as template and mutated forward primer (carrying a mutated site 2 and a SphI restriction site) were used for amplification. The second PCR was performed using the isolated fragment from PCR 1 as the reverse primer. The isolated product of the second PCR was digested with SacI and SalI and ligated to the pmiRGLO vector. The positive control AldoA reporter plasmid was generated similarly. The 3'-UTR of human AldoA was PCR-amplified and was

then cloned in the pmirGLO vector between the SacI–XbaI sites (for primer sequences, see Table S1).

The Following transfections (24hr or 48hr), the cells were lysed with passive lysis buffer (Cat#E1941; Promega) according to manufacturer's instructions. Firefly and Renilla luciferase activity was assessed using the Dual Luciferase Reporter Assay system (Cat#E1910; Promega) on a luminometer Mithras 2000 (Centro XZ, LB960, Berthold Technologies, Bad Wildbad, Germany). The luciferase activity was normalized to Renilla luciferase activity. Readings were taken in triplicates.

TLR4 inhibition

Inhibition of TLR4 *in vitro* in Raw264.7 cells was performed as previously reported[55,56]. CLI-095 inhibitor (0.5µg/ml 10%DMSO, InvivoGen, France) was added to the culture medium one hour prior to macrophage activation with lipopolysaccharides (LPS).

Macrophage activation with LPS

Activation of RAW264.7 murine macrophages was performed as previously reported with LPS-EB Ultrapure (100ng/ml or 5ng/ml, tlrl-3pelps, InvivoGen, France). LPS-EB Ultrapure was added to the culture medium. 24 hours post activation the medium and the cells were collected for further analysis.

RNA Extraction

Total RNA was isolated from mice liver tissues samples, or from cells grown in culture, using Trizol reagent (Life Technologies, Paisley, UK). In the first day of extraction, 1ml Trizol was added to 50 mg of murine tissue and homogenized, or added to cells grown in monolayer. The lysates were incubated for 5 min at RT. 200µl Chloroform were added to the Trizol-containing lysates, mixed by vortexing for 20sec and incubated for 3min at RT. After that, the samples were centrifuged for 15min at 14000 x g at 4°C, the upper aqueous phase was transferred into fresh tube and the RNA was precipitated in 100% Ethanol at -20°C

overnight (ON). Next day, the samples were centrifuged for 40min at 14000 x g at 4°C. The pellets were washed (X2) with 85% cold ethanol and re-dissolved in 50µl DDW-DEPC for 10 min at 65°C . RNA yield and quantity were determined using a Nanodrop spectrophotometer ND-1000 (Thermo scientific, Wilmington, DE). For RNAseq samples only, RNA was extracted using MaXtract High Density (QIAGEN, Redwood City, CA) according to the manufacturer's instructions. Integrity of the RNA was analyzed by a ND-1000 V3.7.1, according to the manufacturer's instructions. Only samples with an RNA integrity number (RIN) of 9 or greater were sequenced.

Quantitative real time RT-PCR

cDNA was synthesized from total RNA using the Quanta Biosciences qScript ™ cDNA Synthesis Kit (Cat#95047-100; Quanta Biosciences, Beverly, MA, USA) for mRNA analysis, and using the qScript™ microRNA cDNA Synthesis Kit (Cat#95107-100) for microRNAs analysis. Quantitative PCR (qPCR) of microRNAs and mRNA was performed using the SYBR Green PCR Kit (Cat#84018 and Cat#84071, Quanta Biosciences, Beverly, MA, USA). The fold expression and statistical significance were calculated using the $2^{-\Delta\Delta Ct}$ method. All experiments were performed in triplicates. Primers used for Real-Time-PCR are shown in Table S1. Target mRNAs were normalized to HPRT. Target microRNAs were normalized to U6 or miR-39.

RNA-Seq

Quality control measures were applied to raw FASTQ files, including trimming of low-quality bases and residual adapters as well as filtering out short reads. These measures were performed with Trim_Galore v0.6.1 wrapper tool https://github.com/FelixKrueger/TrimGalore. File assessment before and after quality control was conducted with FastQC v0.11.8 http://www.bioinformatics.babraham.ac.uk/projects/fastqc. Subsequently, reads were aligned

to the transcriptome GRCm38 [mm10] utilizing Salmon v1.10.0, a tool for fast and bias-aware quantification of transcript expression[57]. Differential gene expression analysis was performed with DESeq2 v1.40.2[58]. Based on differential genes lists, heatmaps and volcano diagrams are plotted with standard R functions and packages. Gene set enrichment analysis for pathways and GO terms was performed with Broad Institute GSEA utility V4.2.2: GSEA software, and Molecular Signature Database (MSigDB)[59]. For this analysis, genes were ranked by their signed t-statistics as adopted from the differential gene expression step. Gene sets include mouse GO terms, wiki-pathway and Hallmark sets.

Single Cell analysis

For isolation of single cells, livers were perfused with PBS and harvested in cold PBS. A 50mg portion was collected in disassociation buffer (0.15% Collagenase P (Roche) in HBSS) and incubated at 37˚c until complete disassociation. Cells were treated with ACK for the removal of red blood cells. Cells were washed and strained and kept in full growth medium (DMEM supplemented with 10% fetal calf serum (FCS) (Thermo Scientific, Waltham, MA, USA)). Dead cells were removed using Dead Cell Removal Kit (Miltenyi Biotec, Auburn, USA) according to the manufacturer protocol. Cells were counted manually, and dead cell percentages were calculated using trypan blue. Only samples with less than 10% dead cells were used for sequencing. The cells were subjected to the Chromium Next GEM Single Cell 3′ GEM, Library & Gel Bead Kit v3.1 (10× Genomics, CA, USA) and libraries were prepared for sequencing following manufacturer company instructions. Sequencing was done using NovaSeq 6000 system machine (Illumina) with the following sequencing conditions: 28 bp (Read1) and 90 bp (Read2) using the NovaSeq 6000 SP Reagent Kit v1.5 (100 cycles) (Illumina). Raw FASTQ files were quantified with cellranger count utility, V7.1.0, by 10x Genomics, and mapped to mm10 reference. Filtered barcode-feature matrices were next analyzed with Seurat package, V5.0.3, R package designed for QC, analysis, and exploration

of single-cell RNA-seq data[60]. Quality control measures included the exclusion of cells with an exceedingly low number of expressed genes, a high proportion of mitochondrial gene content, or extreme read counts (either too low or too high). After quality control, cell clustering was conducted using Seurat pipelines. Multiple clustering profiles were generated and evaluated. The prominent clustering scheme integrated treated and untreated samples at weeks 2 and 6 and utilizing the SCT transform method. Additionally, a separate clustering profile focused exclusively on samples from week 2. Cell types were affiliated to clusters using a set of predefined gene markers. Stain plots are based on normalized expression data and a three-color scheme which maps to low, average, and above average expressions. Differentially expressed genes between clusters or between samples or between cell subsets were resolved with Seurat FindMarkers utility. These sets of genes were then submitted for GSEA analysis as described earlier in the RNA-Seq section. Gene network interactions were explored with CellphoneDB, V4.0.0, repository of curated receptors, ligands and their interactions and a tool to interrogate single-cell transcriptomics data[61]. Tables of p-values and interaction strength were generated with CellphoneDB statistical analysis method and further analyzed with custom R scripts.

ELISA

Proteins were measured in serum or measured in the medium of treated cells by ELISA according to the manufacturer's instructions. CXCL1 (ab216951, Abcam, Cambridge, United Kingdom), IL6 (ab222503, Abcam, Cambridge, United Kingdom), EPO (442707, Biolegend, San Diego, California), SAA (DY2948-05, R&D systems Minneapolis).

Human data Patient selection and inclusion

The human autoimmune hepatitis (AIH) clinical data included in this study was approved by the local ethics committee (Approval number PV4081) and was conducted in accordance

with the Declaration of Helsinki. All patients gave their written informed consent for data sharing and tissue analysis.

Inclusion Criteria:

Initial Diagnose of AIH

Age > 18

consent

Exclusion Criteria:

treatment with Glucocorticoide or Anti-TNF-AK at the time of

sampling (Hydroxychloroquin allowed -> 1 Patient)

AIH/PBC Overlap and other causes of acute hepatitis

Normal liver tissue FFPE material was provided from material stored in pathology that was not used for diagnostic purposes and is allowed to use without consent according to the UKE, Hamburg, Germany regulations.

Clinical assessment of AIH patients

Clinical scoring of Inflammation activity and Fibroisgrade were assessed as we previously reported[62].

Gene expression analysis of AIH patient liver biopsies

Identification of differentially expressed genes from bulk mRNA-sequencing data was performed from normalized counts processed with DESeq2 package[58] by Wilcoxon rank-sum test with post-hoc correction for multiple testing. Gene set enrichments were analyzed with gprofiler2[63].

microRNA sequencing of human liver samples

RNA extracted from liver samples of AIH patients was sequenced by Qaigen according to the following discerption. Sample preparation: A DNase digestion was carried out and the RNA eluted with 14 µl RNase free water. RNA concertation and quality were measured by Qubit

RNA BR (20-1000 ng) and RNA ScreenTape (25-500 ng/µl). Library preparation and sequencing: The library preparation was done using the QIAseq miRNA Library Kit (QIAGEN). A total of 100 ng RNA was converted into miRNA NGS libraries. After adapter ligation, UMIs were introduced in the reverse transcription step. The cDNA was amplified using PCR (16 cycles) and during the PCR, indices were added. After PCR, the samples were purified. Library preparation was quality controlled using capillary electrophoresis (Tape D1000). Based on quality of the inserts and the concentration measurements, the libraries were pooled in equimolar ratios. The library pool(s) were quantified using qPCR. The library pool(s) were then sequenced on a NextSeq2000 (Illumina Inc.) sequencing instrument according to the manufacturer instructions (1x75, 1x8). Raw data was de-multiplexed and FASTQ files for each sample were generated using the bcl2fastq2 software (Illumina inc. USA). Read mapping, and quantification of gene expression: All primary analysis was carried out using CLC Genomics Server 21.0.4. The workflow "QIAseq miRNA Quantification" of CLC Genomics Server with standard parameters was used to map the reads to miRBase version 22. In short, the reads were processed by (1) trimming of the common sequence, UMI and adapters, and (2) filtering of reads with length < 15 nt or length > 55 nt. They were then deduplicated using their UMI. Reads are grouped into UMI groups as follows: (1) when they start at the same position based on the end of the read to which the UMI is ligated (i.e., Read2 for paired data), (2) when they are from the same strand, and (3) they have identical UMIs. Groups that contain only one read (singletons) are merged into non-singleton groups if the singleton's UMI can be converted to a UMI of a non-singleton group by introducing an SNP (the biggest group is chosen). All reads that don't map to miRBase, neither with perfect matches nor as isomiRs (maximum 2 mismatches and/or alternative start/end position of 2 nt), were mapped to the human genome GRCh38 with ENSEMBL GRCh38 version 97 annotation. This was carried out using the "RNA-Seq

Analysis" workflow of CLC Genomics Server with standard parameters. The 'Empirical analysis of DGE' algorithm of the CLC Genomics Workbench 21.0.4 was used for differential expression analysis with default settings. It is an implementation of the 'Exact Test' for two-group comparisons developed by Robinson and Smyth, 2008 and incorporated in the EdgeR Bioconductor package Robinson et al., 2010. For all unsupervised analysis, only miRNAs were considered with at least 10 counts summed over all samples. A variance stabilizing transformation was performed on the raw count matrix using the function vst of the R package DESeq2 version 1.28.1. 500 genes with the highest variance were used for the principal component analysis. The variance was calculated agnostically to the pre-defined groups (blind=TRUE). 35 genes with the highest variance across samples were selected for hierarchical clustering.

Statistical analysis

Data were evaluated for significance by two-tailed Student t-test or Mann–Whitney test, as indicated and unless otherwise noted. $P \leq 0.05$ was considered significant for all analyses. Calculations were performed using GraphPad Prism 6.02 software (GraphPad Software, Inc).

Supplementary tables

Table S1. Primers

gene		sequence	manufacturer
miR-122	F	GTTTGGAACCACTGAGGAGTCTGTGAC	IDT-syntezza
genomic region	R	CCATAGGAGAGGTTGGATTTTACTGGTC	IDT-syntezza
Pri-miR-122	F	TTCGGGAACTATGTGGAGTCACTTTG	IDT-syntezza
	R	CGCAAGGCTGCCCTCAAACCCTCAG	IDT-syntezza
IRF2 3'UTR	F	CACAGAGCTCAGAACATTGATTGCTGTGTC	IDT-syntezza
	R	CACAGTCGACGGATGGGATAGGAAGAGAGG	IDT-syntezza
Mutated IRF2 3'UTR	F	ATCTTGCTCTATCTCACAGCATGCTAAGCACCCCCTTCTCTG	IDT-syntezza
AldoA 3'UTR	F	TAACCAGAGCTCCTCTTCGTCTCTAACCACG	IDT-syntezza
	R	ACCAATCTAGAGCTGTTTATTTGGCAG	IDT-syntezza
αSMA	F	GTCCCAGACATCAGGGAGTAA	IDT-syntezza
	R	TCGGATACTTCAGCGTCAGGA	IDT-syntezza
TGFβR2	F	CCGCTGCATATCGTCCTGTG	IDT-syntezza
	R	AGTGGATGGATGGTCCTATTACA	IDT-syntezza
Col3a1	F	CCTGGCTCAAATGGCTCAC	IDT-syntezza
	R	CAGGACTGCCGTTATTCCCG	IDT-syntezza
Col4a3	F	CAAAGGCATCAGGGGAATAACT	IDT-syntezza
	R	ATCCGTTGCATCCTGGTAAAC	IDT-syntezza
CCR1	F	CTCATGCAGCATAGGAGGCTT	IDT-syntezza
	R	ACATGGCATCACCAAAAATCCA	IDT-syntezza
CCR5	F	TTTTCAAGGGTCAGTTCCGAC	IDT-syntezza
	R	GGAAGACCATCATGTTACCCAC	IDT-syntezza
CXCl12	F	TGCATCAGTGACGGTAAACCA	IDT-syntezza
	R	TTCTTCAGCCGTGCAACAATC	IDT-syntezza
CXCR4	F	CCATGGAACCGATCAGTGTGA	IDT-syntezza
	R	TTGCCGACTATGCCAGTCAAG	IDT-syntezza
TNFα	F	GAAAAGCAAGCAGCCAACCA	IDT-syntezza
	R	CGGATCATGCTTTCTGTGCTC	IDT-syntezza
IL1β	F	TTCAGGCAGGCAGTATCACTC	IDT-syntezza
	R	GAAGGTCCACGGGAAAGACAC	IDT-syntezza
IL6	F	ATACCACTCCCAACAGACCTGTCT	IDT-syntezza
	R	CAGAATTGCCATTGCACAACTC	IDT-syntezza
IFNγ	F	CCACGGCACAGTCATTGAAAG	IDT-syntezza
	R	AGAGATAATCTGGCTCTGCAGG	IDT-syntezza
IL12a	F	CTGTGCCTTGGTAGCATCTATG	IDT-syntezza
	R	GCAGAGTCTCGCCATTATGATTC	IDT-syntezza
IL12b	F	TGGTTTGCCATCGTTTTGCTG	IDT-syntezza
	R	ACAGGTGAGGTTCACTGTTTCT	IDT-syntezza
TLR2	F	ACAACTTACCGAAACCTCAGAC	IDT-syntezza
	R	CACACACCCCAGAAGCATCACATG	IDT-syntezza
TLR3	F	GCGTTGCGAAGTGAAGAACTTG	IDT-syntezza
	R	GTTGTTCAAGAGGAGGGCGAAT	IDT-syntezza
TLR4	F	TTCAGAACTTCAGTGGCTGG	IDT-syntezza
	R	TGTTAGTCCAGAGAAACTTCCTG	IDT-syntezza
AldoA	F	GGGTGATCCTCTTCCACGAGA	IDT-syntezza
	R	AGGGGCACCACACCCTTATC	IDT-syntezza
CXCL1	F	CTGGGATTCACCTCAAGAACATC	IDT-syntezza
	R	CAGGGTCAAGGCAAGCCTC	IDT-syntezza
CXCL2	F	CCAACCACCAGGCTACAGG	IDT-syntezza
	R	GCGTCACACTCAAGCTCTG	IDT-syntezza
IRF2	F	AATTCCAATACGATACCAGGGCT	IDT-syntezza
	R	GAGCGGAGCATCCTTTTCCA	IDT-syntezza
Hamp	F	CCTATCTCCAGCAACAGATG	IDT-syntezza
	R	AACAGATACCACAGGAGGGT	IDT-syntezza
HJV	F	CAGCTCCCCGGTTTCGT	IDT-syntezza
	R	TGGTAGACTTTCTGGTCAATGCA	IDT-syntezza
SAA1	F	ACACCAGGATGAAGCTACTCACCA	IDT-syntezza
	R	CCCTTGGAAAGCCTCGTGAACAAA	IDT-syntezza
SAA2	F	CAGGAGACACCAGCAGGATGAAG	IDT-syntezza

	R	CCCGAGCATGGAAGTATTTGTCTC	IDT-syntezza
SPP1	F	GTGATTTGCTTTTGCCTGTTTG	IDT-syntezza
	R	GAGATTCTGCTTCTGAGATGGG	IDT-syntezza
CD8	F	GTTCAGCAAGGAAAACGAAGGC	IDT-syntezza
	R	GCACAGGTGAGGGAGTTCG	IDT-syntezza
HPRT	F	GCGATGATGAACCAGGTTATGA	IDT-syntezza
	R	ATCTCGAGCAAGTCTTTCAGTCCT	IDT-syntezza
miR-122-5p	F	HSA-MIR-122-5P PCR ASSAY 20-YP00205664	Qiagen
C.el. miR-39	F	CEL-MIR-39-3P MIRCURY PCR ASSAY 20-YP00203952	Qiagen
U6	F	U6 SNRNA (V2) MIRNA PCR ASSAY	Qiagen

Table S2. IHC primary antibodies

Antibody	Catalog	Company
F4/80	MCA497G	BioRad
CD3	MCA1477	BioRad
CD4	Ab183685	ABCAM
CD8	14-0808-82	Invitrogen
Ly6g	Ab238132	ABCAM
cleaved caspase 3	9661	Cell signaling
pSTAT3	D3A7	Cell Signaling
NFkB- P65	D14E12 xP(R)	Cell Signaling
IRF2	ab124744	ABCAM
SPP1	AKm2A1	Santa Cruz

Table S3. Antibodies for FACS analysis

Target	Clone	Fluorochrome	Vendor	Cat	RRID	Stock conc. mg/mL	Final conc. mg/ml	Dilution Factor	Staining condition
Viability	N/A	Zombie NIR	BioLegend	423106	N/A	N/A	N/A	1600	Extracellular
CD8α	53-6.7	Biotin Strep-AF350	BioLeg./ThermoFS	100703/S11249	AB_312742	0.5	0.0025	200	Extracellular
LAG-3 (CD223)	C9B7W	BUV737	BD Biosciences	741820	AB_2871155	0.2	0.008	25	Extracellular
Ly6G	1A8	PerCP	BioLegend	127653	AB_2616998	0.2	0.008	25	Extracellular
TIGIT	1G9	PE-Dazzle594	BioLegend	142110	AB_2566572	0.2	0.008	25	Extracellular
F4/80	QA17A29	PE-Fire640	BioLegend	157319	AB_2904297	0.2	0.008	25	Extracellular
CD160	7H1	PE	BioLegend	143003	AB_10960740	0.2	0.004	50	Extracellular
ICOS-L (CD275)	REA990	VioBright 515	Miltenyi	130-116-450	AB_2727547	0.15	0.003	50	Extracellular
CXCR3	CXCR-3173	BV650	BioLegend	126531	AB_2563160	0.2	0.004	50	Extracellular
XCR1	ZET	BV510	BioLegend	148218	AB_2565231	0.2	0.004	50	Extracellular
TCR gd	GL3	BUV563	BD Biosciences	748993	AB_2873390	0.2	0.002	100	Extracellular
CD127	A7R34	PE-Cy5	ThermoFisher Scientific	15-1271-83	AB_468794	0.2	0.002	100	Extracellular

CD27	LG.3A10	BUV496	BD Biosciences	741094	AB_2870693	0.2	0.002	100	Extracellular
BTLA (CD272)	6A6	PE-Fire700	BioLegend	N/A	N/A	0.18	0.0018	100	Extracellular
PD-L1 (CD274)	MIH5	PerCP-eF710	ThermoFisher Scientific	46-5982-80	AB_2573818	0.2	0.002	100	Extracellular
PD-1 (CD279)	29F.1A12	BV605	BioLegend	135220	AB_2562616	0.2	0.002	100	Extracellular
TIM-3 (CD366)	RMT3-23	BV711	BioLegend	119727	AB_2716208	0.2	0.002	100	Extracellular
CD39	Y23-1185	BUV395	BD Biosciences	567264	N/A	0.2	0.002	100	Extracellular
CD62L	MEL-14	BV570	BioLegend	104433	AB_10900262	0.025	0.00025	100	Extracellular
CD69	H1.2F3	BUV615	BD Biosciences	751593	AB_2875587	0.2	0.002	100	Extracellular
CD45	30-F11	SparkViolet 538	BioLegend	103179	AB_2890675	0.5	0.005	100	Extracellular
PVR (CD155)	TX56	BUV661	BD Biosciences	749991	AB_2874213	0.2	0.002	100	Extracellular
CD11c	N418	SparkBlue550	BioLegend	117365	AB_2860633	0.5	0.0025	200	Extracellular
CD19	6D5	SparkNIR685	BioLegend	115567	AB_2819828	0.5	0.0025	200	Extracellular
CD44	IM7	AF700	BioLegend	103026	AB_493713	0.5	0.0025	200	Extracellular
Galectin-9	108A2	PE-Cy7	Biolegend	137913	AB_2750156	0.2	0.001	200	Extracellular
KLRG-1	2F1	APC-eF780	ThermoFisher Scientific	47-5893-82	AB_2573988	0.2	0.001	200	Extracellular
Ly6C	HK1.4	BV785	BioLegend	128041	AB_2565852	0.2	0.001	200	Extracellular
NK1.1	S17016D	APC/Fire810	BioLegend	156519	AB_2894654	0.2	0.001	200	Extracellular
Siglec-H	eBio440	SuperBright 436	ThermoFisher Scientific	62-0333-82	AB_2784802	0.2	0.001	200	Extracellular
CD11b	M1/70	SparkYG593	BioLegend	101281	AB_2892261	0.5	0.00125	400	Extracellular
CD25	PC61	BV480	BD Biosciences	566202	AB_2739522	0.2	0.0005	400	Extracellular
ICOS (CD278)	C398.4A	BV750	BioLegend	313557	AB_2876616	0.15	0.000375	400	Extracellular
CD4	GK1.5	SparkBlue574	BioLegend	100489	AB_2904268	0.5	0.00125	400	Extracellular
MHC II	M5/114.15.2	PE-Fire810	BioLegend	107667	AB_2894690	0.2	0.0005	400	Extracellular
FoxP3	REA788	Vio R667	Miltenyi	130-111-682	AB_2651766	0.2	0.004	50	Intracellular
CD152 (CTLA-4)	UC10-4B9	BV421	BioLegend	106312	AB_2563063	0.2	0.004	50	Intracellular
Granzyme B	GB11	Pacific Blue	BioLegend	515408	AB_2562196	0.1	0.001	100	Intracellular
Perforin	S16009A	APC	BioLegend	154304	AB_2721463	0.2	0.002	100	Intracellular
Ki67	SolA15	BUV805	ThermoFisher Scientific	368-5698-82	N/A	0.2	0.002	100	Intracellular
CD3	17A2	FITC	BioLegend	100203	AB_312660	0.5	0.00125	400	Intracellular

Table S4. miR-122-5p targets that control fibrosis & autoimmunity

miR-122 target (m -mouse; h - human)	2 weeks padj	**2 weeks ko/wt**	3 weeks padj	**3 weeks ko/wt**	6 weeks padj	**6 weeks ko/wt**	Symbol	Fibrosis	Autoimmune	Name	References (PMID)
m	6.16E-05	**1.8**	9.13E-05	**1.7**	2.97E-35	**5.5**	Adamts 2	v		ADAM Metallo-peptidase With Thrombospondin Type 1 Motif 2	17929299
m & h	3.72E-42	**3.9**	9.55E-37	**3.5**	7.86E-26	**2.9**	Aldoa	v		Aldolase, Fructose-Bisphosphate A	38443347
m & h	3.07E-20	**4.1**	2.40E-24	**4.7**	1.10E-33	**6.3**	Alpl	v		Alkaline Phosphatase, Biomineralization Associated / Tissue Non-Specific Alkaline Phosphatase (Tnap)	31969558
m	5.06E-05	**1.5**	3.38E-10	**1.8**	6.48E-30	**2.9**	Bak1		v	BCL2 Antagonist/Killer 1	19282307
m & h	2.48E-107	**3.9**	1.98E-79	**3.3**	2.57E-63	**3.3**	Bckdk		v	Branched Chain Keto Acid Dehydrogenase Kinase	28597552
m	5.48E-10	**8.6**	6.25E-08	**5.8**	1.91E-12	**30.0**	Bmp8b	v		Bone Morpho-genetic Protein 8b	32694734
m	1.13E-07	**1.9**	2.84E-11	**2.2**	1.20E-16	**3.0**	Capn5		v	Calpain 5	37207905
m	0.242	**1.5**	9.23E-05	**3.5**	1.81E-08	**6.1**	Ccr2	v		C-C Motif Chemo-kine Receptor 2	19441102
m	0.0017	**2.1**	2.06E-06	**3.0**	1.40E-10	**4.3**	Ciita		v	Class II Major Histocompatibility Complex Trans-activator	15808836
m	4.50E-24	**12.1**	6.91E-22	**11.8**	3.41E-26	**19.1**	Cldn7	v		Claudin 7	24737165
m & h	4.85E-18	**2.1**	1.33E-20	**2.2**	4.26E-41	**3.3**	Clic1	v		Chloride Intra-cellular Channel 1.	37924785
m	1.65E-08	**2.9**	3.38E-17	**4.7**	7.94E-46	**14.3**	Ddr1	v		Discoidin domain receptor family, member_1	36809965
m	0.000199	**2.2**	0.000694	**2.1**	2.81E-16	**7.1**	Fbln1	v		Fibulin 1	37674431
m	8.40E-27	**2.2**	8.42E-25	**2.1**	1.91E-47	**3.2**	Fuca2		v	Alpha-L-Fucosidase 2	39681237
m & h	3.46E-22	**3.8**	8.40E-18	**3.4**	5.95E-23	**4.9**	Ihh	v	v	Indian hedgehog	37035456, 37740690
m & h	4.61E-06	**1.5**	4.72E-10	**1.8**	4.39E-27	**2.7**	Iqgap1	v		IQ Motif Containing GTPase Activating Protein 1	35036058
m & h	2.83E-06	**1.8**	1.09E-14	**2.6**	9.80E-47	**5.8**	Klf6	v		Kruppel Like Factor 6; Core	28864835

										Promoter Element Binding Protein (Copeb)	
m	1.37E-58	**13.7**	3.34E-87	**25.9**	5.53E-103	**37.6**	Lcn2	v		Lipocalin 2	32968110
m	0.000244	**2.1**	4.73E-08	**2.9**	6.72E-18	**5.0**	Neat1	v	v	Nuclear Para-speckle Assembly Transcript 1 (lncRNA)	28864835, 37716986
m	2.93E-06	**3.3**	6.84E-09	**4.4**	1.32E-11	**6.0**	Pdgfb	v		Platelet Derived Growth Factor Subunit B	16842882
m	9.19E-07	**2.1**	0.0132	**1.5**	6.02E-11	**2.9**	Thbs2	v		Thrombospondin 2	38379585
m	4.73E-06	**2.1**	2.98E-05	**2.0**	2.66E-19	**4.2**	Trim47	v		Tripartite Motif Containing 47	29291351

Supplementary figure legends

Fig. S1. Clinical data and correlations of miR-122 expression in AIH patients A-B. miR-122 levels measured by RNAseq in RNA extracted from liver samples of patients with chronic autoimmune hepatitis (AIH), correlates negatively with clinical scoring of (A) Inflammation activity and (B) Fibrosis grade[64].

Fig. S2. Expression of miR-122 and precursor expression and secretion in mouse AIH model. Pri-miR-122 and miR-122 levels measured by RT-PCR in RNA extracted from liver and serum samples of late stage AIH mice 20-28 weeks old. The Primary transcript of miR-122 (primiR-122) as well as the mature miR-122 are significantly decreased as well as the levels of secreted miR-122 measured in the serum. * $p<0.05$, ** $p<0.01$

Fig. S3. miR-122 regulates TLR3 via IRF2. A. Experimental design- mice were injected by hydrodynamic tail vein injection (HTVI) with antagomiR-122 or antago-Scr (control). 24 hours post HTVI mice were injected IP with LPS. 5 hours later livers were harvested for analysis. **B**. miR-122 levels measured by RT-PCR in RNA extracted from harvested livers. **C.** Heatmap of differentially expressed genes (DEGs) in livers of mice were miR-122 was inhibited compared to control. **D**. Gene expression analysis of RNA from livers of mice where miR-122 was inhibited by antagomir-122 and antago-Scr control: Gene set enrichment analysis (GSEA). **E**. GSEA hallmarks of IFNα and IFNγ in mice were miR-122 is inhibited compared to control. **F**. Allegro motif discovery analysis. The mouse binding site of the STAT1::STAT2, IRF2, IRF1 transcription factors (TF) is very similar to the motif shown. **G**. Segal Lab algorithm (cut off < = - 10.00) score of these TFs in human. **H**. Binding potential score of miR-122 to targets in listed genes, including the validated miR-122 target AldoA. **I.** MiR-122 reporter assay: Regulation by miR-122 compared to miR-scr as measured by the luciferase signal of a reporter plasmid bearing the 3'UTR the validated miR-122 target

AldoA, human IRF2 (hIRF2) and hIRF2 where the binding site for miR-122 is mutated (hIRF2mut). **J.** IHC staining of IRF2 in liver tissue section from mice where miR-122 was inhibited compared to control (n=3). **K.** Relative gene expression of IRF2 gene in mice where miR-122 was inhibited compared to control. **L**. Schematic depicting the regulation of IRF2 transcription factor on the expression of TLR3 gene. Host cell factor C2 (HCFC2) facilitates IRF2 association with its DNA target. * $p<0.05$

Fig. S4. Generation of miR-122 KO mouse and phenotype follow-up. A. Depiction of the genetic modification performed on miR-122 KO mice. The sequences of miR-122-5p and miR-122-3p are indicated (blue) within the genomic region of miR-122 in the mouse, and specifically the seed sequence. Two sgRNA target sites (yellow)were utilized in order to create a ~60bp deletion and the cleavage site is indicated (red arrow). **B**. Genotyping was performed on DNA extracted from tissue samples of inbred mice using primers that span the genomic region of miR-122. The PCR amplicon of WT mice was of 380bp, while the amplicon from miR-122 KO mice was 320bp. **C.** Realtime PCR relative expression of miR-122-5p in RNA extracted from livers of miR-122 KO mice and WT controls at the age of 6 weeks. **D**. Directional gene heatmap – Fold change (FC) in livers of miR-122 KO mice compared to WT control from week 2 through week 3 to week 6. Fold change (FC) of miR-122 KO/ WT increases from A->C: Negative at A, around zero at B and positive at C. **E.** Routine MRI monitoring of miR-122 KO mice revealed the development of HCC tumors as early as the age of 35 weeks (representative MRI image and image of harvested liver- tumors indicated by yellow arrows and yellow outline). Mice bearing tumors were euthanized and a survival curve shows the tumor frequency in miR-122 KO mice and WT control mice up to the age of 78 weeks. **F.** Single cell analysis of cells isolated from livers of miR-122 KO mice and WT controls and the age of 2 weeks indicating the cells expressing HAMP in the Hepatocyte associated cluster. **G.** Real time PCR relative expression of Hamp and Hjv in

RNA extracted from livers of miR-122 KO mice and WT controls at the age of 6 weeks. * $p<0.05$, ** $p<0.01$, *** $p<0.001$, **** $p<0.0001$

Fig. S5. IL-12 expression is upregulated in effector CD8 T cells of miR-122 KO mice. A. Gene set enrichment analysis (GSEA) of differentially expressed genes within the single cell RNAseq CD25^{+} CD62L^{-} effector T cell cluster of miR-122 KO mice compared to WT control at the ages of 2 weeks and 6 weeks ranked by normalized enrichment score (NES) **B**. Spearman correlation between mRNA expression of IL-12A or IL-12B in liver biopsies of AIH patients and the corresponding histological Modified Hepatitis Activity Index (mHAI) score. **C**. Spearman correlation between mRNA expression of IL-12A or IL-12B in liver biopsies of AIH patients and the respective expression levels of miR-122. **D**. Analysis of single cell RNAseq data: expression of IL12a in the B cell cluster of miR-122 KO mice compared to WT control at the age of 6 weeks. IL12 expression is found in the B cell cluster (turquoise) and IL12Rb1 and IL12Rb2 in the T cell cluster (soft pink). ** $p<0.01$, *** $p<0.001$, **** $p<0.0001$

Fig. S6. T cell depletion in miR-122 KO mice. A. Experimental design- mice were injected IP with αCD4 and αCD8 or IgG control starting from the age of 2 weeks and harvested at the age of 6 weeks. **B.** liver histology of miR-122 KO T cell depleted mice and controls, T cells (CD3), Macrophages (F4/80) and fibrosis (Sirius red). **C.** Real time PCR relative gene expression measured in livers of miR-122 KO mice where T cells were depleted and controls. ** $p<0.01$, *** $p<0.001$

Fig. S7. Microbiome depletion alleviates the inflammatory phenotype in miR-122 KO mice. A. Experimental design- mice were treated with antibiotics cocktail (ABX) or DDW as control starting from before birth and harvested at the age of 6 weeks. **B.** Confirmation of microbiome depletion by measurement of Fecal DNA in feces samples of miR-122 KO mice

treated with ABX or DDW. **C.** Liver histology of miR-122 KO treated with ABX cocktail or DDW as control. Macrophages (F4/80) and Fibrosis (Sirius red). **D.** Real time PCR relative gene expression measured in livers of miR-122 KO treated with ABX or DDW as control of TLR4 and downstream mediators of inflammation TNFα, IL-1β, IL-6, IL-12a, IL12b and IFNγ. * p<0.05, ** p<0.01, *** p<0.001

Fig. S8. SAA significantly increases in miR-122 KO mice A. Gene expression analysis of RNAseq data from livers of miR-122 KO mice compared to WT controls at the ages of 2, 3 and 6 weeks indicating the significance (pv) and fold change (L2FC) of the genes SAA1 and SAA2. **B.** Real time PCR confirmation of differentially expressed SAA genes. **C.** SAA levels measured by ELISA in serum of miR-122 KO mice and WT controls at the age of 6 weeks. **D.** Single cell RNAseq analysis of livers of miR-122 KO mice and WT controls at the ages 2 weeks and 6 weeks indicating the clusters of cells expressing SAA1 and SAA2. * p<0.05, ** p<0.01, *** p<0.001

Fig. S9. Spp1 significantly increases in miR-122 KO mice. A. Spp1 Immunohistochemical staining in miR-122 KO mice and WT control at the age of 6 weeks. **B.** Gene expression analysis of RNAseq data from livers of miR-122 KO mice compared to WT controls at the ages of 2, 3 and 6 weeks indicating the significance (pv) and fold change (L2FC) of Spp1 gene. **C.** Real time PCR confirmation of Spp1 gene. **D.** Single cell RNAseq analysis of livers of miR-122 KO mice and WT controls at the age of 6 weeks indicating the clusters of cells expressing Spp1. * p<0.05, ** p<0.01.

Fig. S10. Clinical data of miR-122 KO mice bacterial infection compared to WT control. A. Clinical scoring of miR-122 KO mice compared to WT control infected with $5x10^7$ CFU MRSA or saline as control. Weight and body temp was documented prior to harvesting. **B**. Blood chemistry- liver damage markers (alkaline phosphatase (ALP), Lactate Dehydrogenase

(LDH), serum glutamic-oxaloacetic transaminase (SGOT) and Serum glutamic pyruvic transaminase (SGPT)) measured in the serum **C.** Hematology- white blood cells (WBC), Lymphocytes, Neutrophils and Monocytes absolute number of cells (Abs.) . * $p<0.05$, ** $p<0.01$, *** $p<0.001$, **** $p<0.0001$

Supplementary Figure 1. Clinical data and correlations of miR-122 expression in AIH patients

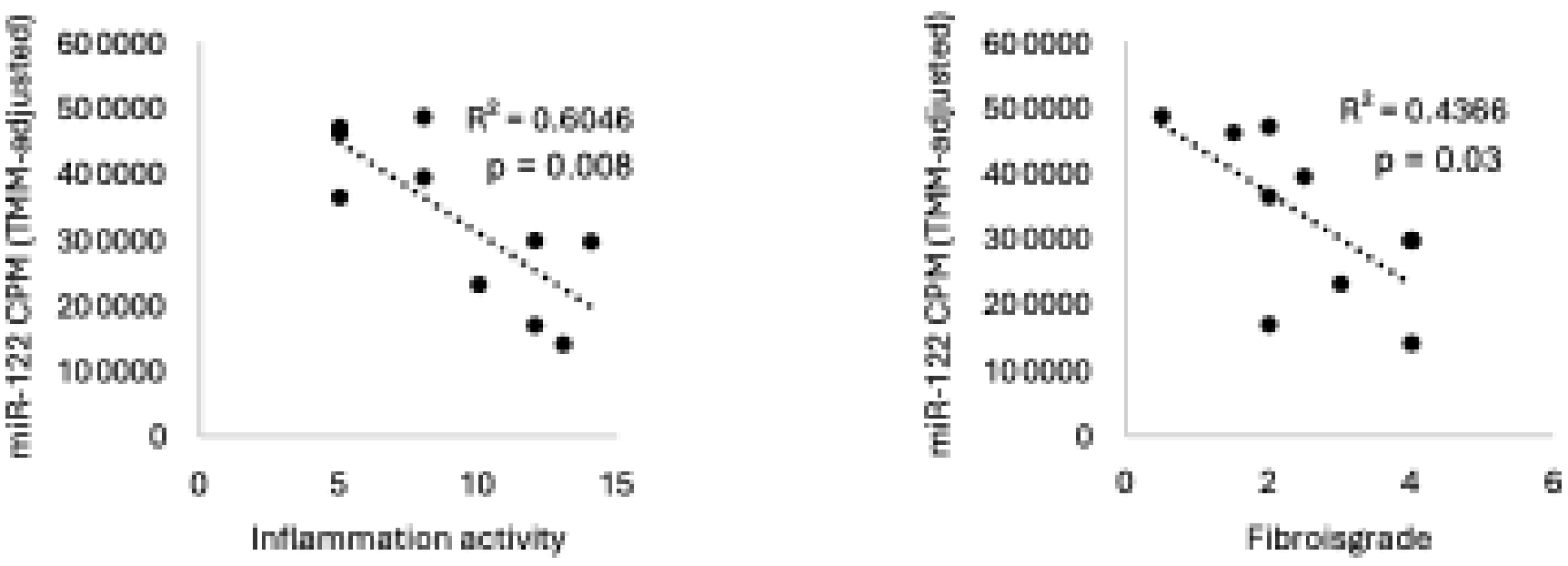

Supplementary Figure 2. Expression of miR-122 and precursor expression and secretion in mouse autoimmune liver disease model

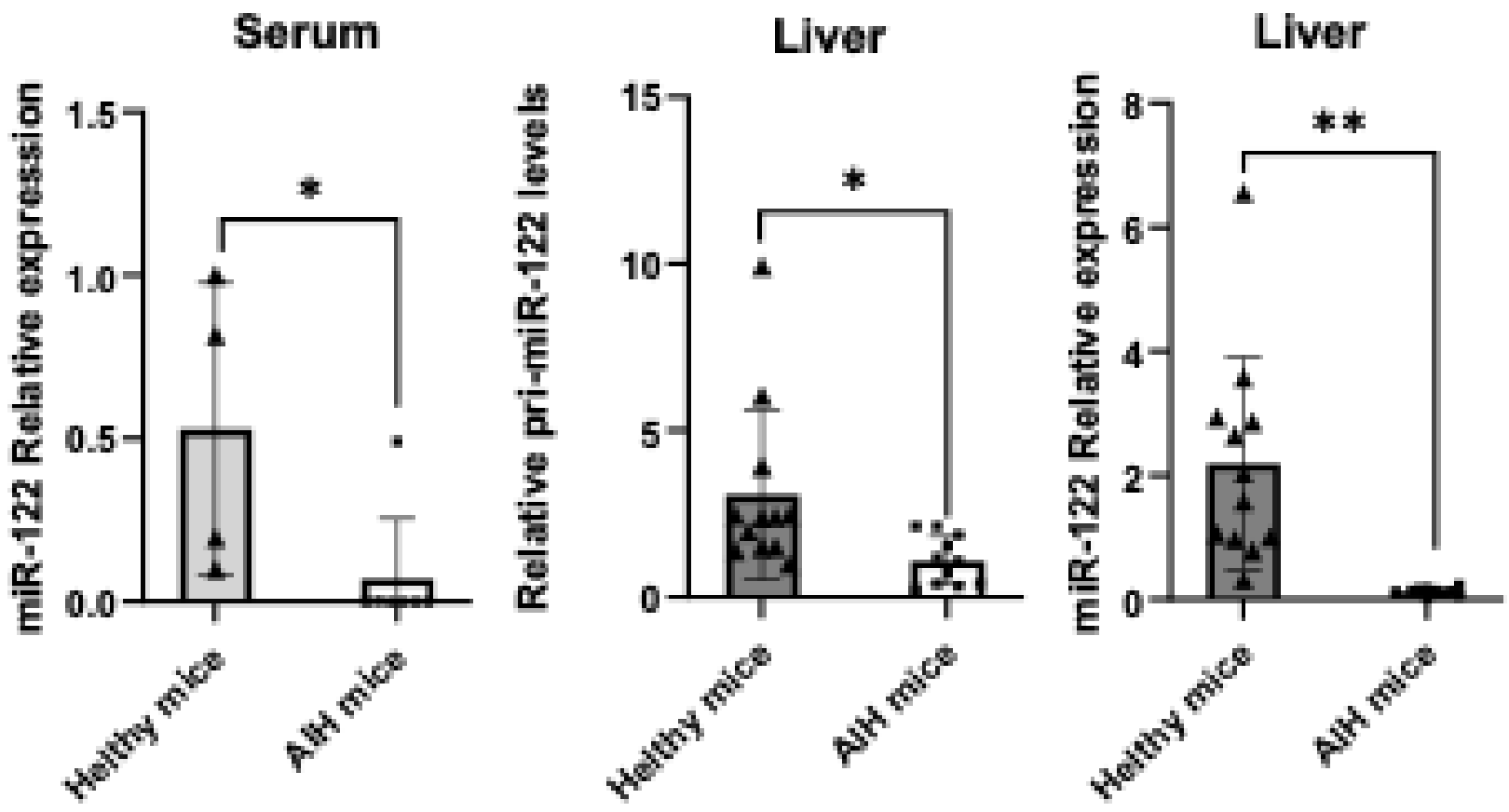

Supplementary Figure 3. miR-122 regulates TLR3 via IRF2

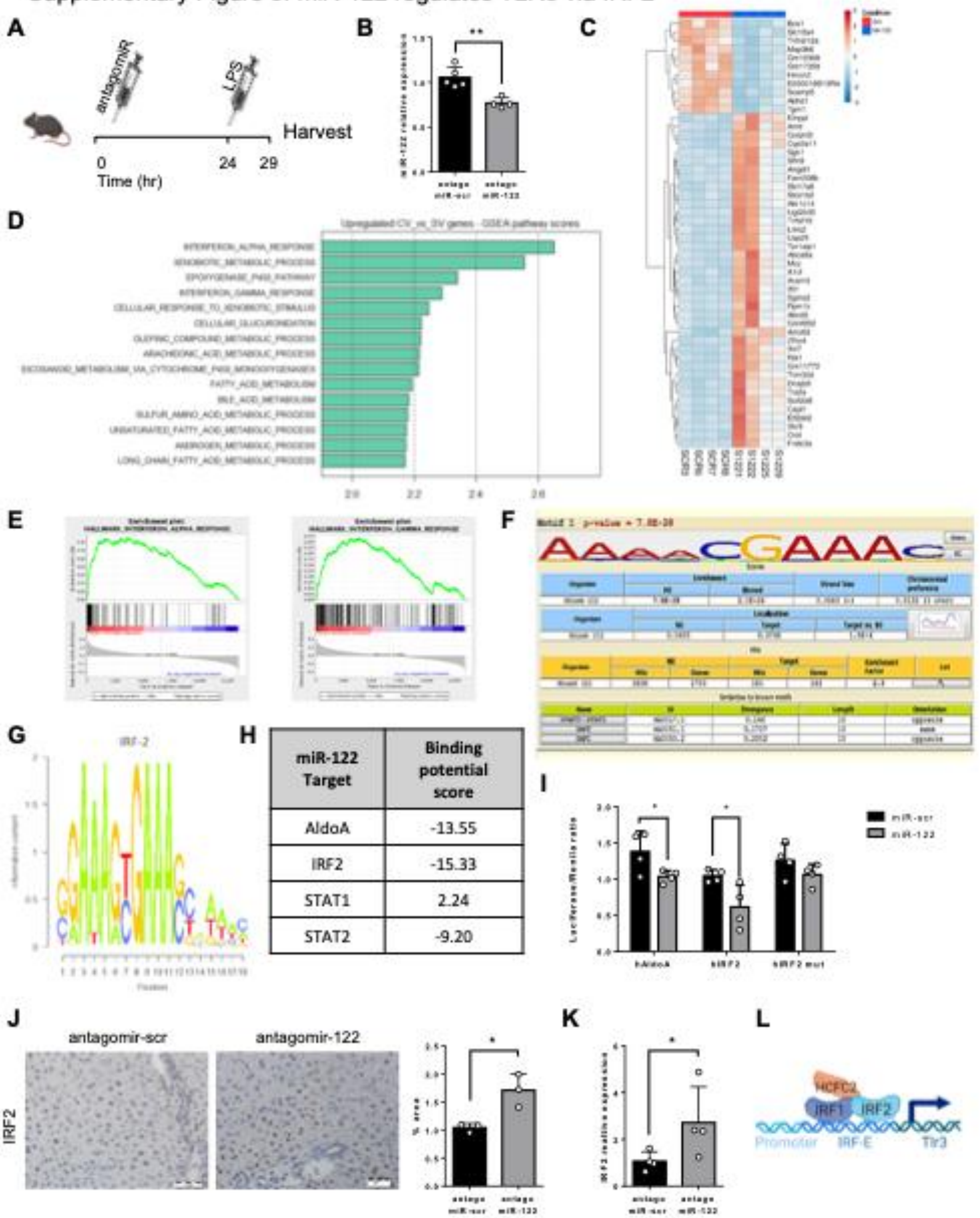

miR-122 Target	Binding potential score
AldoA	-13.55
IRF2	-15.33
STAT1	2.24
STAT2	-9.20

Supplementary Figure 4. Generation of miR-122 KO mouse and phenotype follow-up

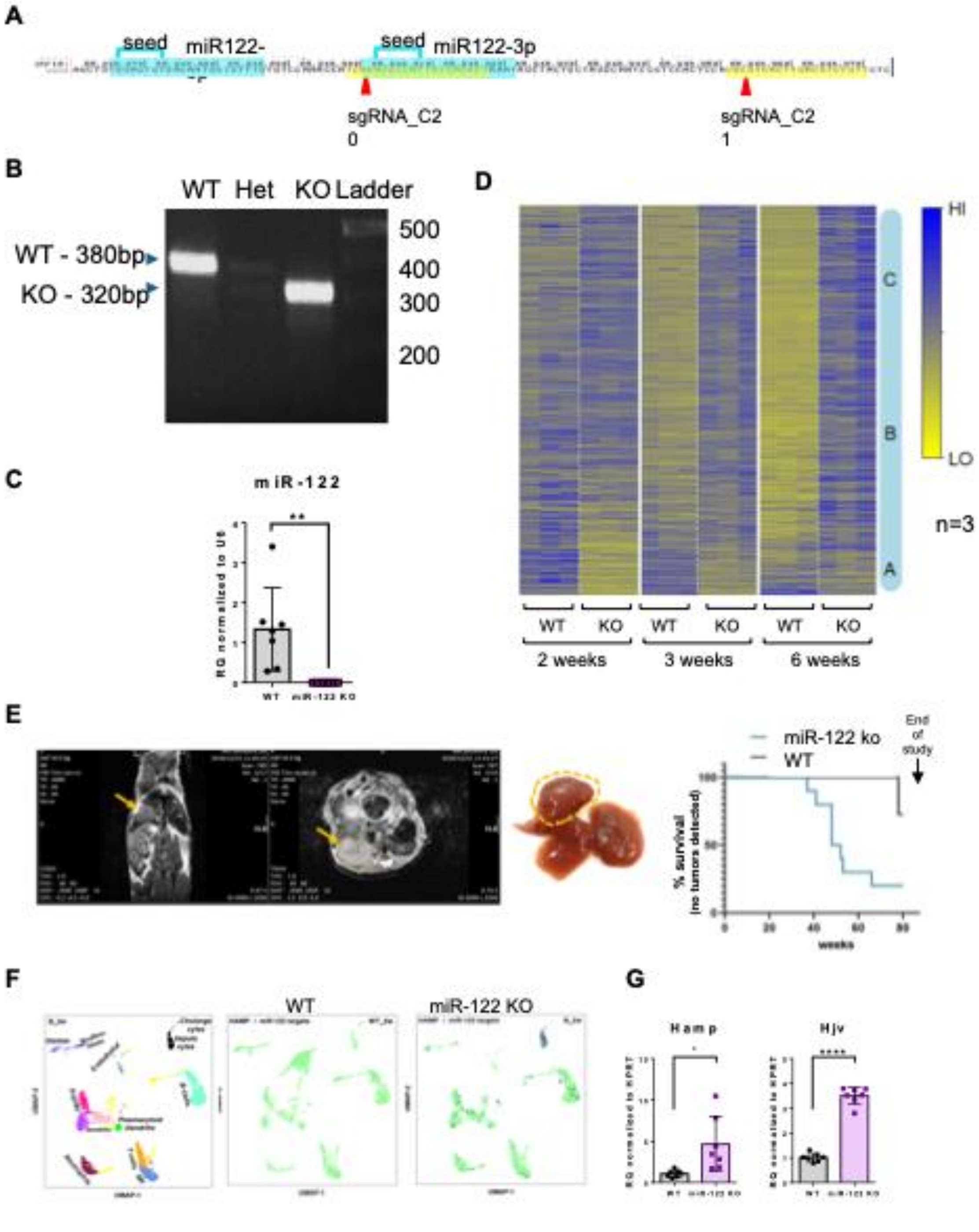

Supplementary Figure 5. IL-12 expression is upregulated in effector CD8 T cells of miR-122 KO mice

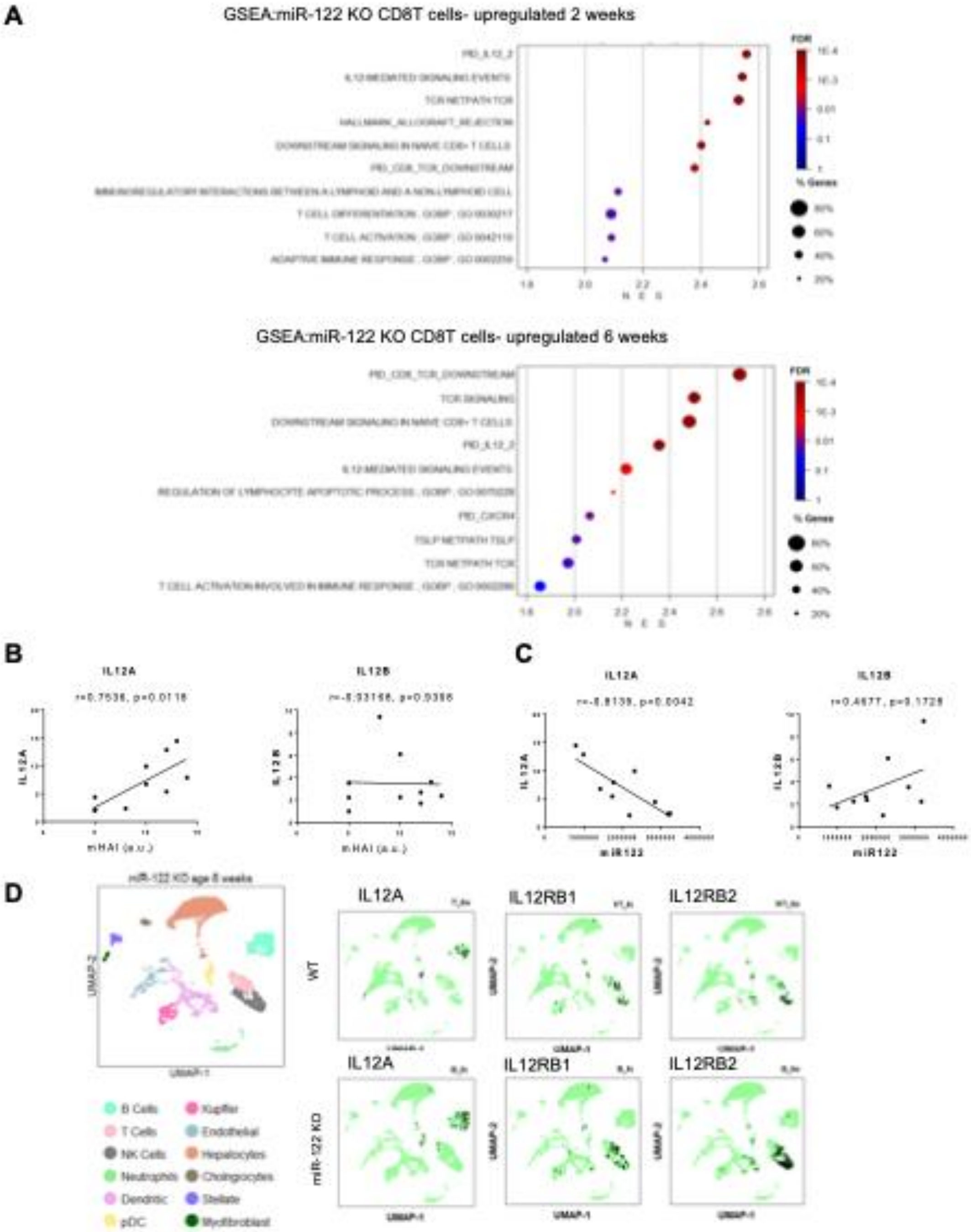

Supplementary Figure 6. T cell depletion in miR-122 KO mice

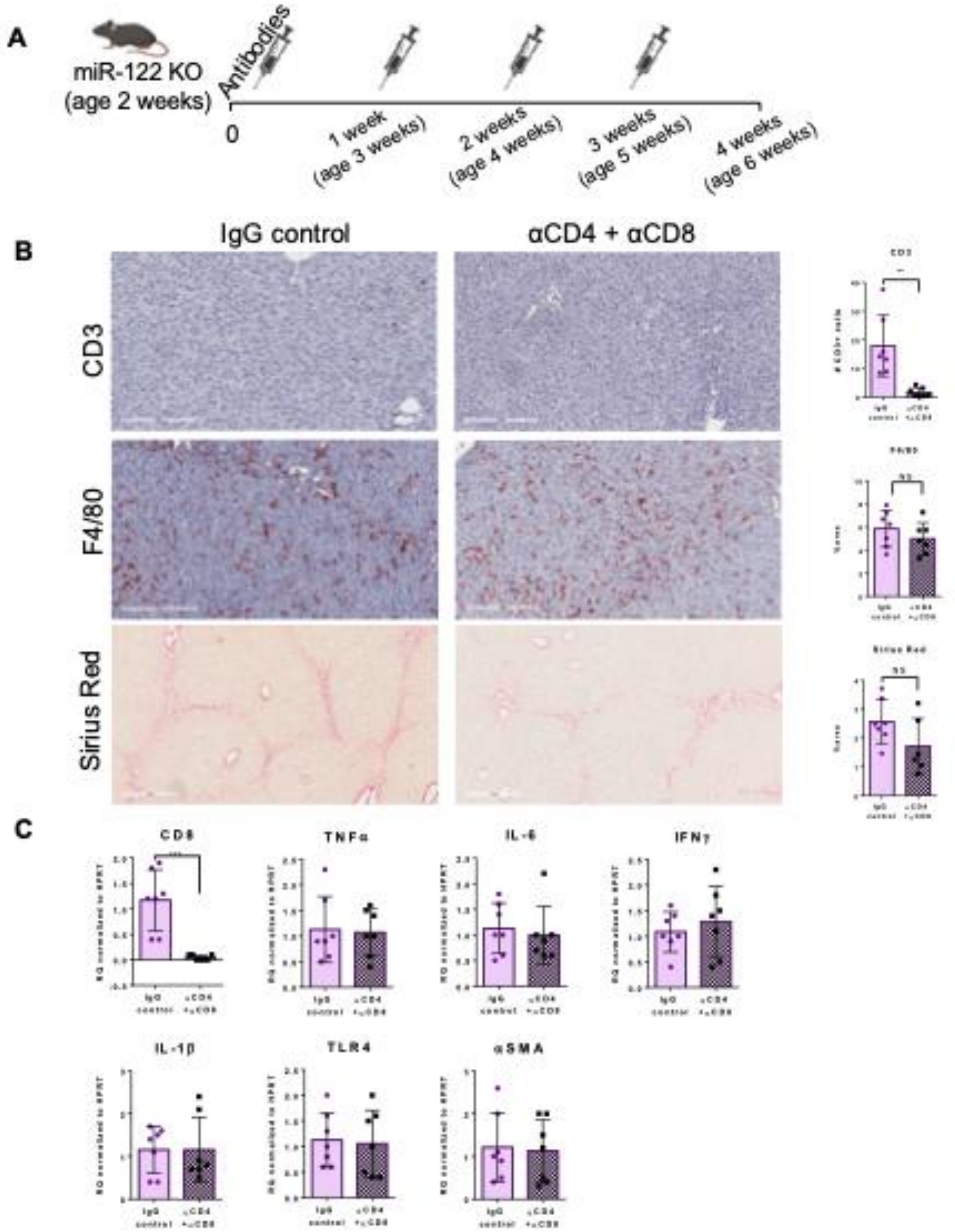

Supplementary Figure 7. Microbiome depletion alleviates the inflammatory phenotype in miR-122 KO mice

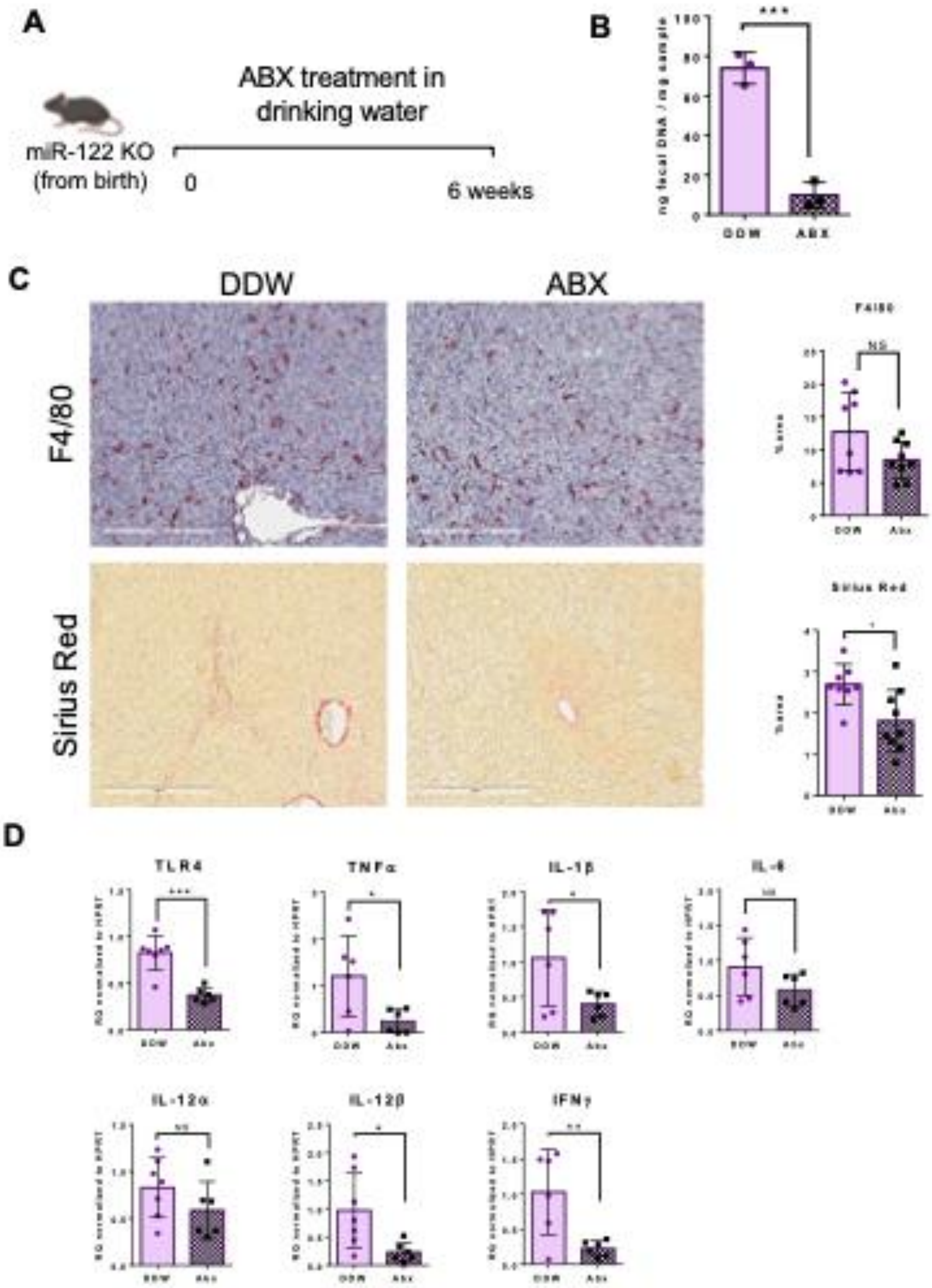

Supplementary Figure 8. SAA significantly increases in miR-122 KO mice

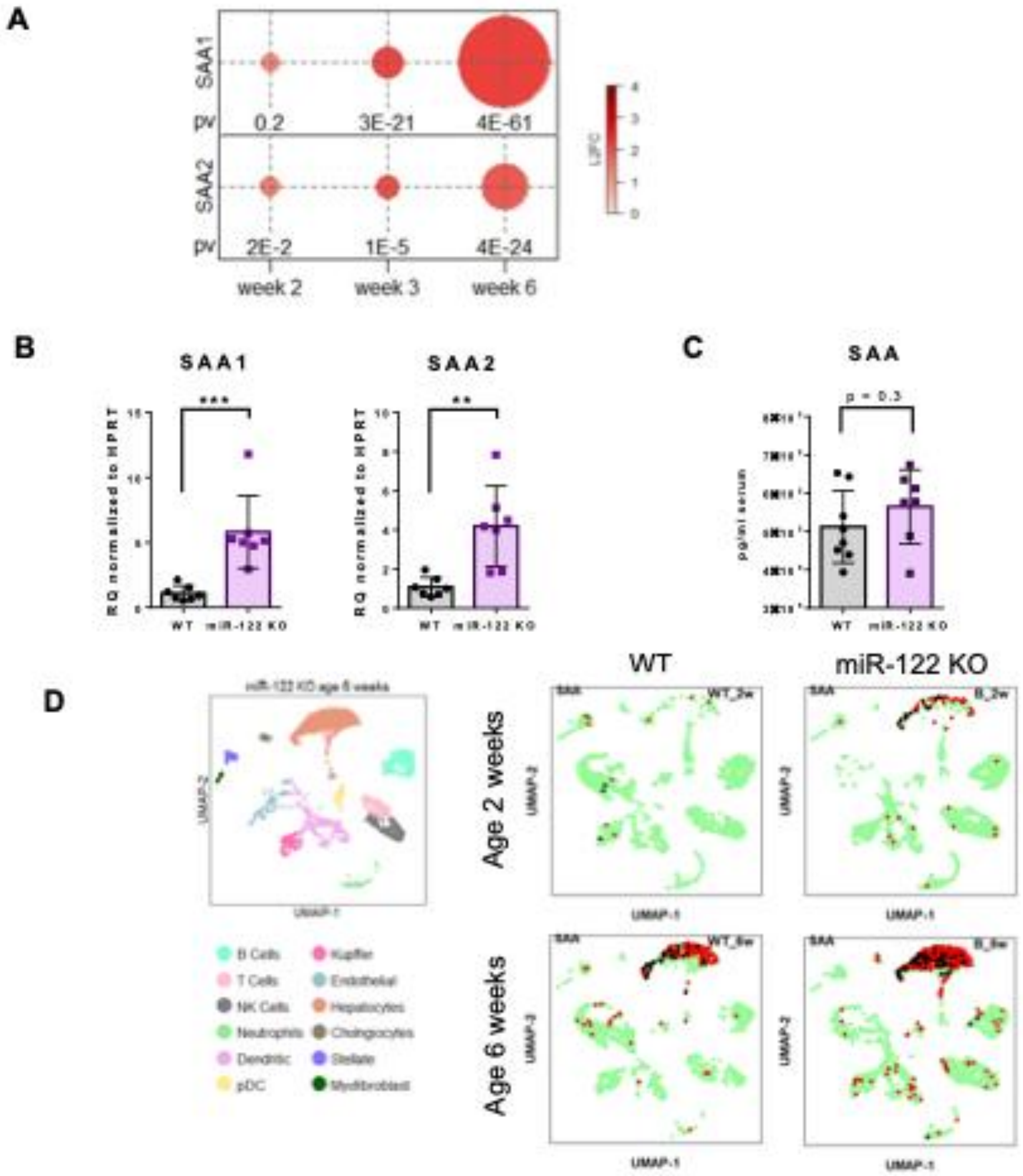

Supplementary Figure 9. Spp1 significantly increases in miR-122 KO mice

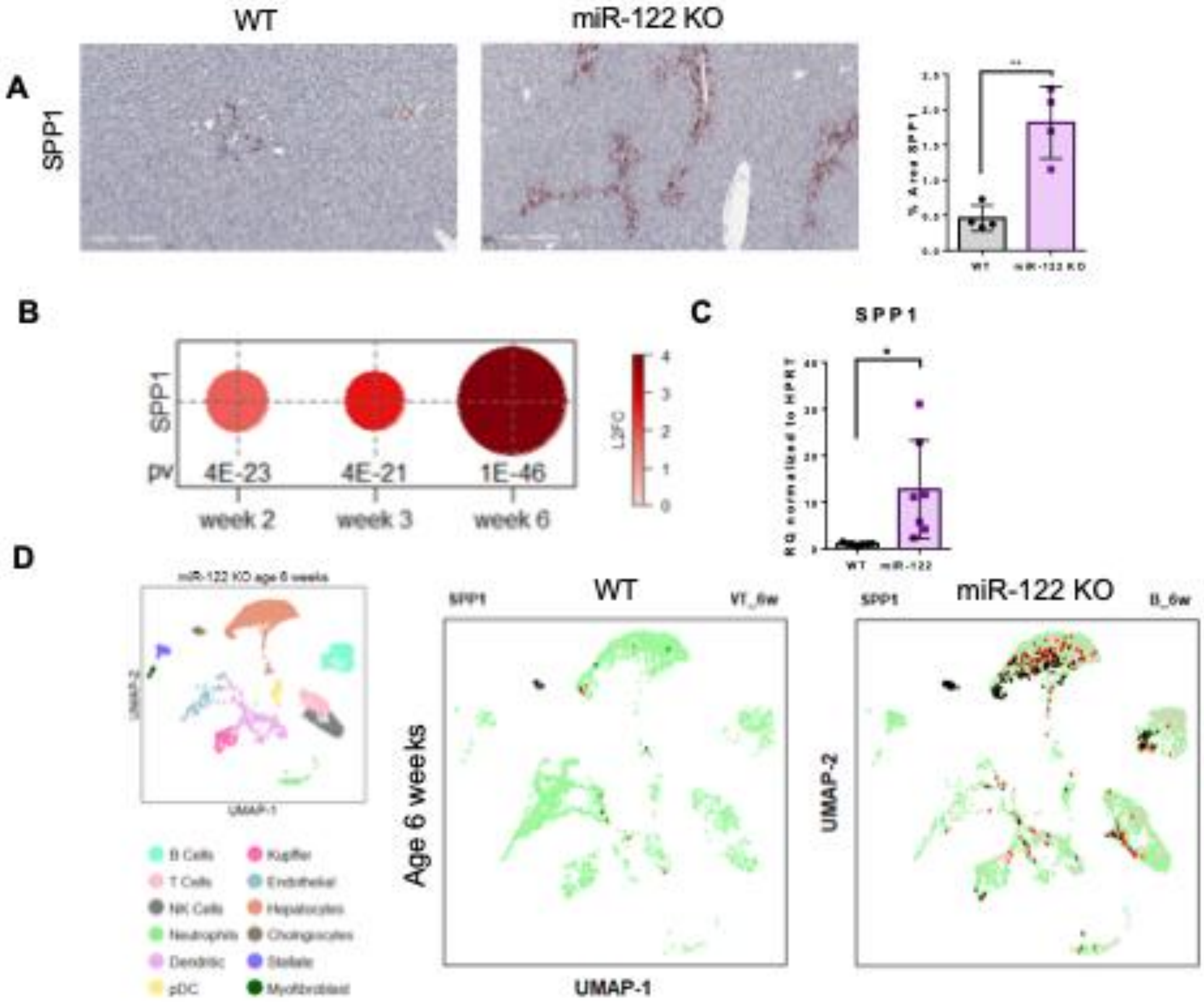

Supplementary Figure 10. Clinical data of miR-122 KO mice bacterial infection compared to WT control

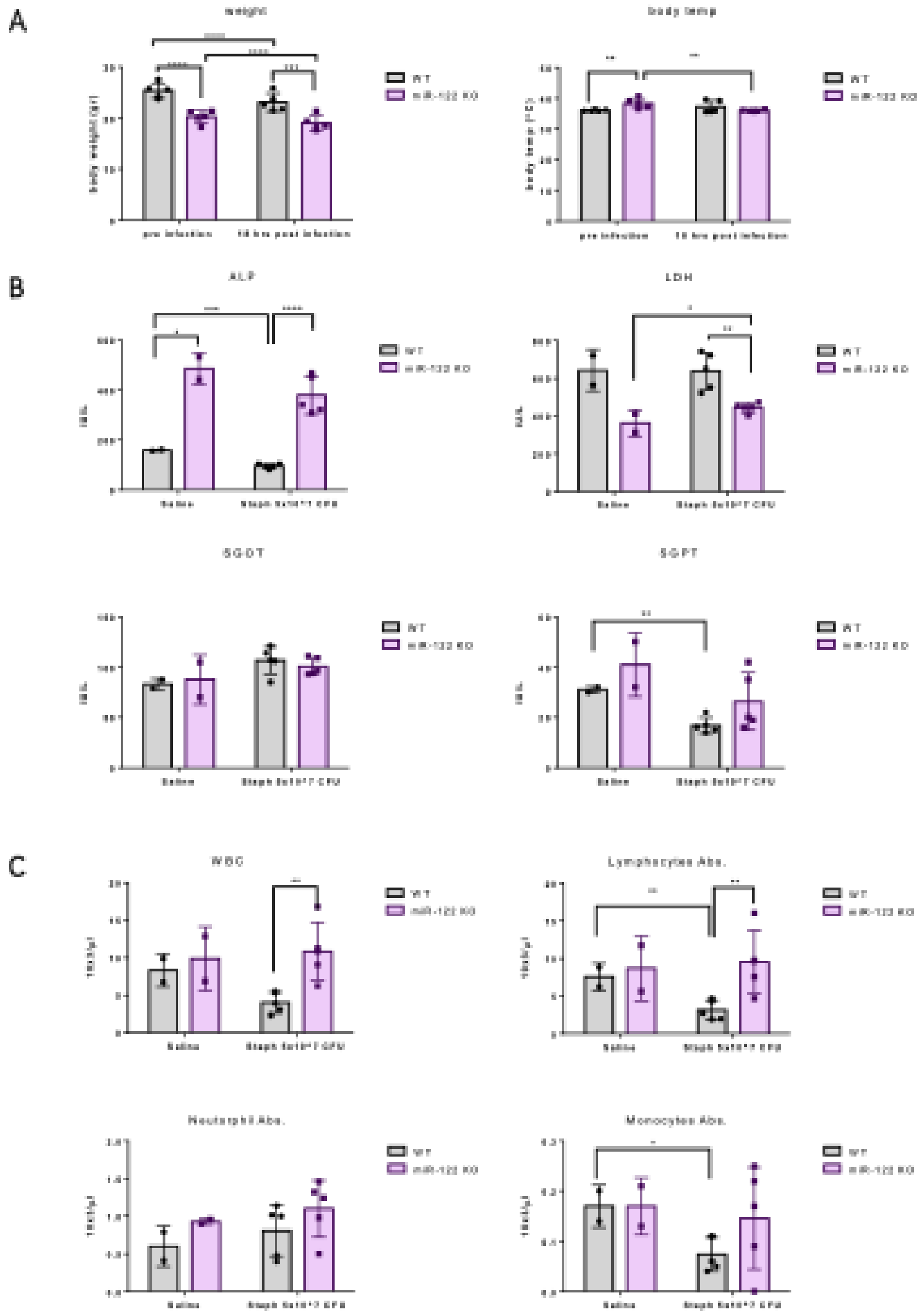

Supplement: Supplementary file 1 [file mmc1.pdf]
