# Supplementary material for: MiR-122 regulates liver tolerance
Source: JHEP Rep. 2026 Aug 29;8(12):102020. doi: 10.1016/j.jhepr.2026.102020 (PMC13770681; doi:10.1016/j.jhepr.2026.102020)

Research article

JHEP|Reports

MiR-122 regulates liver tolerance

Authors

Maytal Gefen, Shanny Layani, Emma Klahr, …, Mathias Heikenwälder, Hilla Giladi, Eithan Galun

Correspondence

eithan.galun@gmail.com, eithang@hadassah.org.il (E. Galun), m.heikenwaelder@dkfz-heidelberg.de (M. Heikenwälder).

Graphical abstract

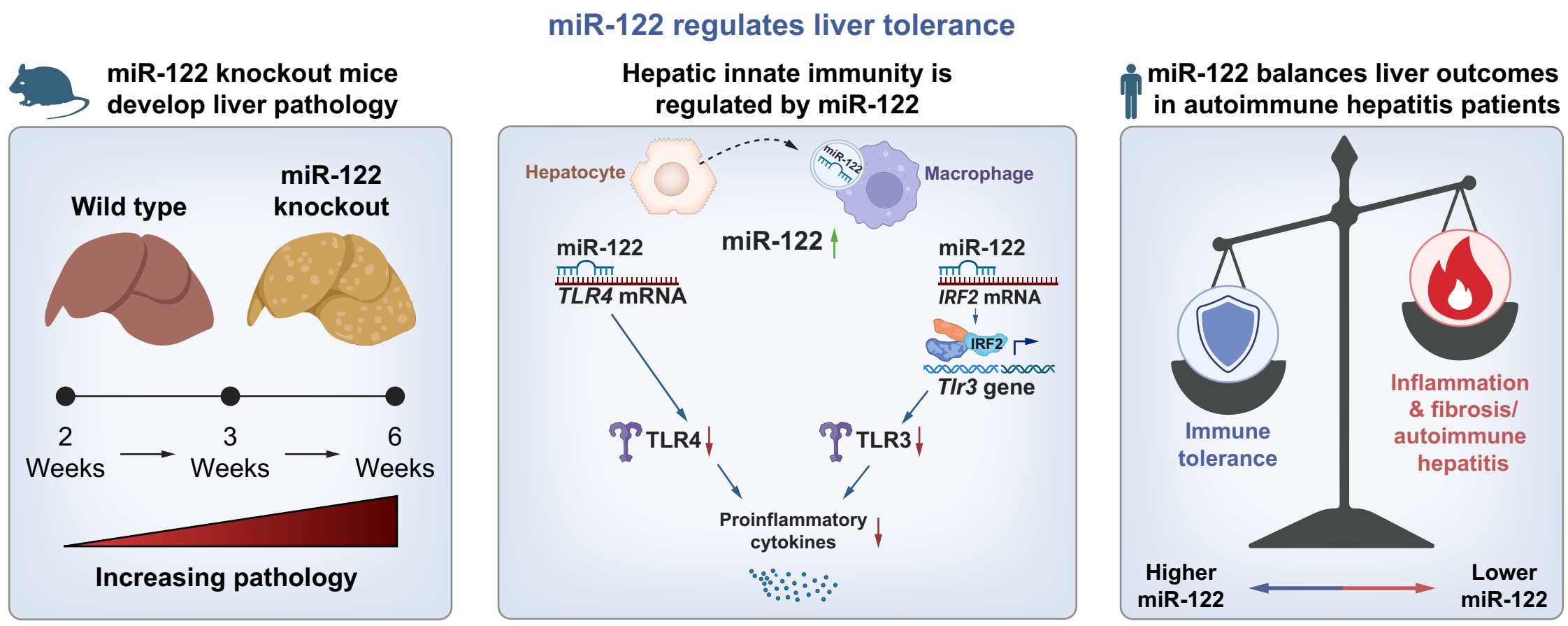

Highlights:

- MiR-122 regulates liver immune privilege.
- MiR-122 influences liver inflammation.
- MiR-122 deficiency causes fibrosis.

Impact and implications:

The liver is an immune-privileged organ, an adaptation likely evolved to prevent robust immune responses against material absorbed from the gastrointestinal tract. This tolerance creates a sanctuary for pathogens—including bacteria, hepatitis viruses, and parasites such as *Plasmodium*—within the liver parenchyma and similarly permits the development of primary liver malignancy and metastatic seeding. We found that miR-122 regulates hepatic immune tolerance, positioning miR-122 modulation as a potential therapeutic strategy for infectious, autoimmune, malignant, and metastatic liver diseases.

https://doi.org/10.1016/j.jhepr.2026.102020

© 2026 The Authors. Published by Elsevier B.V. on behalf of European Association for the Study of the Liver (EASL). This is an open access article under the CC BY license (http://creativecommons.org/licenses/by/4.0/). JHEP Reports, 2026, 8, 1–13

MiR-122 regulates liver tolerance

Maytal Gefen[1,†], **Shanny Layani**[1,†], Emma Klahr[1], Zohar Shemuelian[1], Johannes Brandi[2], Thomas Jacobs[2], Christiane Steeg[2], Lorenz Adlung[3], Christoph Kilian[3], Merav Ordan[1], Oren Gordon[1], Nathalie Abudi[1,4], Rinat Abramovitch[1,4], Yuval Nevo[5], Sharona Elgavish[5], Hadar Benyamini[5], Jonathan Monin[5], Elina Zorde Khvalevsky[1], Nofar Rosenberg[1], Aurelia Markezana[1], Dayana Yaish[1], Adi S. Yehezkel[1], Alina Simerzin[1], Mila Rivkin[1], Osher Amran[1], Daniel S. Goldenberg[1], Amnon Peled[1], Jonathan Axelrod[1], Jacob Rachmilewitz[1], Samuel Huber[3], Nicola Gagliani[3,6], Christoph Schramm[3], Sören Weidemann[3], Johannes Herkel[3], Antonella Carambia[3], Rifaat Safadi[7], Abed Khalaileh[8], Ashraf Imam[8], Daniela Heide[9], Jenny Hetzer[10], Stefan Rose-John[11], Mathias Heikenwälder[9,10,*], Hilla Giladi[1], Eithan Galun[1,*]

JHEP Reports **2026**. vol. 8 | 1–13

Check for updates

Background & Aims: The liver is an immune-privileged organ, yet it needs to overcome infections. We aimed to explore the role of the liver-specific microRNA miR-122 in liver immune tolerance.

Methods: We developed a miR-122 knockout (KO) mouse model and assessed immune, inflammatory, and fibrogenic responses from early postnatal stages. Innate and adaptive immune alterations were studied in miR-122 KO mice. The transcriptomic profiles of patients with autoimmune hepatitis (AIH) were compared with those of these KO mice.

Results: MiR-122 KO mice developed liver inflammation and fibrosis as early as 2 weeks of age. An inverse correlation between miR-122 expression and inflammation/fibrosis was observed in both KO mice (p <0.01in both at Weeks 2 [n = 6] and 3 [n = 6] of KO mice age) and human AIH samples (p <0.01, n = 9). KO mice shared transcriptomic and phenotypic features with human AIH. RNA sequencing identified more than 5,000 differentially expressed genes between wild-type and KO mice, including upregulated chemokines, immune checkpoints, and pro-fibrotic markers. IRF2, a TLR3 transcription factor, was identified as a novel direct target of miR-122.

Conclusions: MiR-122 is a key regulator of liver immune tolerance, modulating inflammation and fibrosis via distinct pathways. Its absence enhances immune activation and accelerates fibrosis. These findings identify miR-122 as a promising therapeutic target for autoimmune liver diseases.

© 2026 The Authors. Published by Elsevier B.V. on behalf of European Association for the Study of the Liver (EASL). This is an open access article under the CC BY license (http://creativecommons.org/licenses/by/4.0/).

Introduction

The liver microenvironment must be balanced and steadily equilibrated between immune responses and immune tolerance.[1] On the one hand, the liver must maintain immune tolerance toward gut-derived material absorbed through the gastrointestinal tract. On the other hand, it must react against invading pathogens to protect its tissue integrity and function. The unique tolerogenic liver immune microenvironment enables liver transplantation without human leukocyte antigen (HLA) matching.[2] However, it also makes the liver a preferred site for metastases.[2,3] The mechanisms enabling this immune tolerance are only partially understood.[4,5] We hypothesized that an efficient and comprehensive regulatory mechanism should involve concurrent regulation of different arms of the immune system and that, accordingly, a microRNA that is expressed at high levels in the liver and is capable of simultaneously suppressing genes and pathways may play a central role in the immune tolerance process.[6] One potential microRNA that could fulfill this assignment is the liver-specific miR-122, which is expressed at over 100,000 copies per hepatocyte and constitutes 70% of all microRNAs contained in the liver.[7] MiR-122, produced by hepatocytes, is secreted[8] and can bind messages of target genes in adjacent and remote cells reached via the circulation. We were interested in understanding the mechanism underlying the liver microenvironment's balance between the liver's mission of combating infection and tolerating microbiome flushed from the gastrointestinal tract. Inflammatory mediators are central to anti-infection modalities. One example of a central mediator of inflammation and immunity in the liver is TNFα.[9] We have previously reported that TNFα is central to liver immunity and inflammation[10,11] and, interestingly, at the same time, TNFα mediates miR-122 expression.[8] Previous studies[12,13] have shown, and we confirm in this study, that hepatocellular carcinoma (HCC) develops in the miR-122 knockout (KO) mouse model. Knowing that HCC develops on the background of chronic inflammation, we wondered when all these changes develop and how they could contribute to HCC development. This circuitry led us to investigate postnatal (weeks 2–6 of mouse life) events in the early liver microenvironment that could also affect the development of anemia[14] or HCC

* Corresponding authors. Addresses: Goldyne Savad Institute of Gene Therapy, Hadassah Hebrew University Hospital, Jerusalem, Israel. Tel.: +972 05 7874395 (E. Galun); The M3 Research Center, Medical Faculty, University Clinic Tübingen (UKT), Tübingen, Germany. Tel.: +49 172 1866957 (M. Heikenwälder).
E-mail addresses: eithan.galun@gmail.com, eithang@hadassah.org.il (E. Galun), m.heikenwaelder@dkfz-heidelberg.de (M. Heikenwälder).
† These authors had an equal contribution to this study.
https://doi.org/10.1016/j.jhepr.2026.102020

in miR-122 KO mice, as previously reported.[15] To understand this immune/inflammatory hepatic microenvironment, we generated a new constitutive miR-122 KO mouse using a CRISPR/Cas9 system, omitting a very short genomic region of 59 nt, as opposed to previous studies in which a significantly larger genomic region was excised, which could cause non-relevant off-target effects. We investigated alterations in innate and adaptive immune cells in the liver during early postnatal life and monitored fibrosis development from 2 weeks of age. Herein, we report on mechanisms encompassing the role of miR-122 in regulating liver immune tolerance. Interestingly, in miR-122 KO mice, fibrosis and inflammation are not sequential: inflammation initiates first, followed by fibrosis.[16] This regulation involves the direct and indirect effects of miR-122 on known liver and systemic immune regulators and inflammatory pathways, many of which were reported in specific cases as miR-122 direct and indirect targets. So far, a longitudinal assessment of the whole organ from early life onwards has not been conducted. MiR-122 was shown to execute tumor suppressive effects,[17,18] and we confirm these findings in our data. Furthermore, to the list of immune genes directly targeted by miR-122, we add IRF2, a positive regulator of Toll-like receptor 3 (TLR3), thereby supporting our overall hypothesis that miR-122 is an immune attenuator. The significance of our findings is underscored by the possibility that manipulating miR-122 levels could have clinical significance. It would be beneficial to upregulate miR-122 to suppress tumors in liver metastasis or liver autoimmune diseases.

Materials and methods

Mice and ethical approvals

MiR-122 KO mice (C57BL/6miR-122em2Hhtg) were generated via CRISPR/Cas9-mediated deletion of the miR-122 genomic region (chr18:65,381,932-65,381,997). Full genotyping and colony maintenance protocols are available in the Supplementary Materials and Methods and in Table S1.

In vivo experimental procedures

For MRI monitoring, mice were anesthetized and scanned using a 7T cryogen-free MR scanner (MR Solutions, Guildford, UK) to assess liver pathology. MiR-122 inhibition was achieved via hydrodynamic tail vein injection of antagomiR-122 (7 μg/mouse), followed by lipopolysaccharide (LPS) administration (1 μg/kg, Sigma-Aldrich, Rehovot, Israel). Liver and blood samples were harvested after treatment for analysis. Macrophage depletion and T-cell depletion protocols used clodronate liposomes and anti-CD4/CD8 antibodies, respectively, as detailed in the Supplementary Materials and Methods.

Microbiome depletion

Breeding pairs were treated with an antibiotic cocktail (vancomycin, imipenem, and neomycin) from gestation through weaning. Fecal microbiome quantification was performed using the ZymoBIOMICS DNA Miniprep Kit (Irvine, California, USA).

Molecular and cellular analyses

RNA extraction, quantitative PCR, RNA sequencing (RNAseq), and flow cytometry were performed using established protocols (Supplementary Materials and Methods and Table S1–S3). RNA integrity was verified (RIN ≥9). For histopathology, liver sections were processed for H&E, Sirius Red staining, or TUNEL assay.

Statistics

Data were analyzed using GraphPad Prism (v6.02) with Student's *t* tests or Mann–Whitney tests, as appropriate. A $p \leq 0.05$ was considered statistically significant (San Diego, California, USA).

See the Supplementary Materials and Methods for further details.

Results

MiR-122 in human and mouse chronic AIH

Chronic autoimmune hepatitis (AIH) develops when the liver's tolerance is breached.[19] We first analyzed the correlation between liver inflammation and fibrosis in patients with AIH (Fig. S1A and B), as well as miR-122 expression levels in the liver and found significant negative correlations with both.

Moreover, in a mouse model of AIH,[20] we found a significant decrease in mature miR-122 levels in serum and liver, as well as reduced levels of miR-122 precursor and pri-miR-122 in the inflamed liver tissue (Fig. S2). To further investigate the effect of miR-122 on the immune system, we injected mice with antagomir-122, which represses miR-122 activity, followed by LPS injection to induce an acute immune response (Fig. S3A and B). RNAseq was performed on the livers of these mice, revealing many differentially expressed genes (Fig. S3C). Using KEGG and INGENUITY discovery tools, we found a significantly increased effect on the innate immune system, as indicated by the gene set enrichment analysis (GSEA) score (Fig. S3D). A significant increase in TLR3 expression ($\log_2$ fold change 1.144, $p < 0.0006$) was found. Because the liver is a known sanctuary for viral hepatitis, including chronic HCV infection, the possibility that miR-122 regulates TLR3, a sensor of double-stranded RNA, is important for understanding its role in immune regulation.[21] Moreover, an enrichment of genes associated with interferon-α and interferon-γ (IFNγ) responses was found, supporting the idea of innate immune activation in the absence of miR-122 (Fig. S3E). We further proved that IRF2 is a regulator of TLR3 (Fig. S3F–H) and a direct target of miR-122 (Fig. S3I–K). A schematic illustration of IRF2 at the genomic site of TLR3 is shown in Fig. S3L.

Liver microenvironment of miR-122 KO mice

To further investigate the role of miR-122 in liver tolerance, we generated miR-122 KO mice by deleting a 59-nt genomic region coding for miR-122 (Fig. S4A and B), resulting in a complete shutdown of miR-122 expression (Fig. S4C). In wild-type (WT) mice, the gut microbiome develops already at a very early stage of life.[22] This led us to expect changes in the liver microenvironment of miR-122 KO mice already early in life as

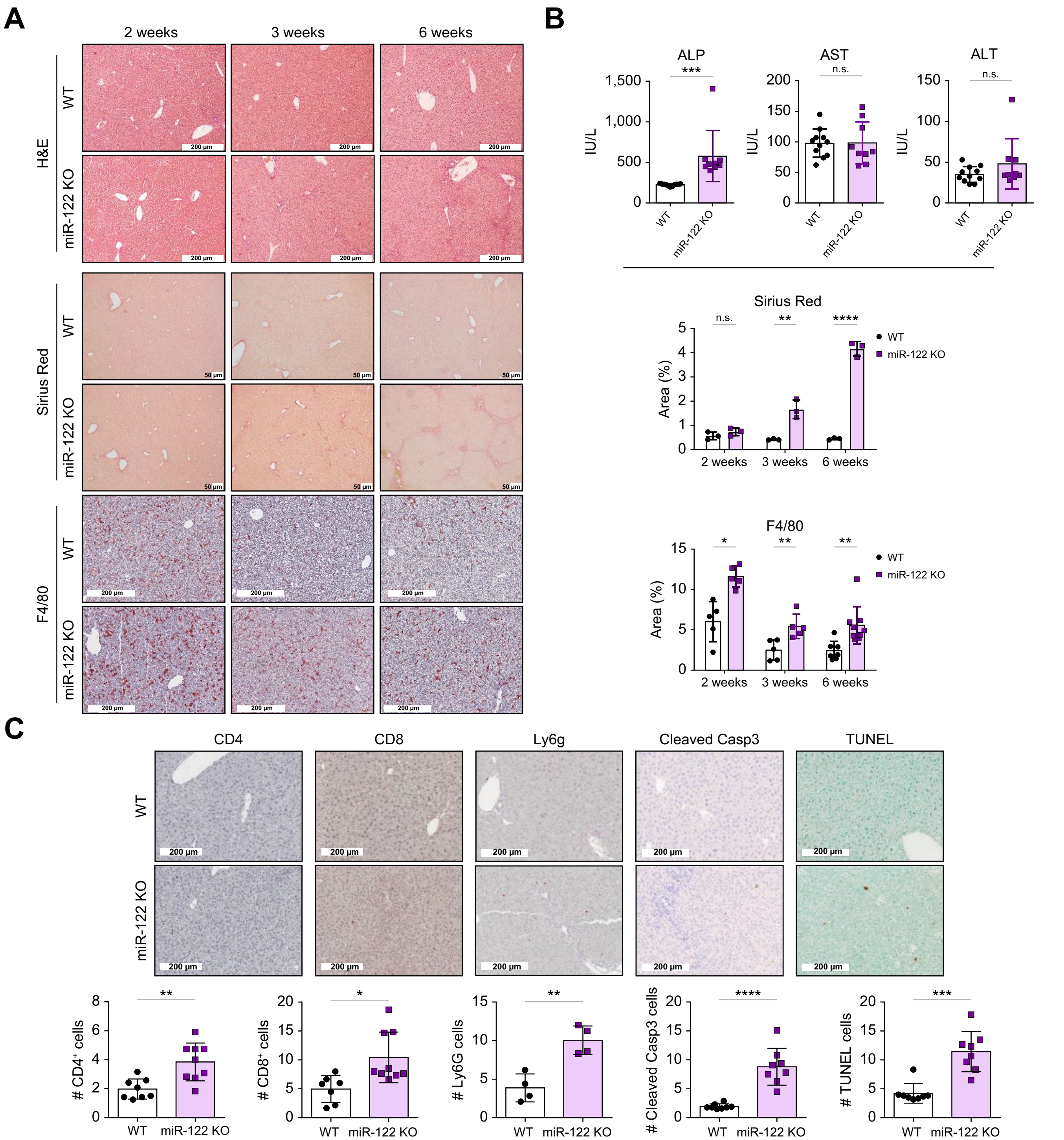

Fig. 1. miR-122 KO mice exhibit enhanced inflammation and fibrosis from early age. (A) Liver histology of miR-122 KO mice and WT controls. H&E stain, fibrosis (Sirius Red), and macrophages (F4/80) at 2, 3, and 6 weeks of age. (B) Liver damage markers (ALP, SGOT (AST), and SGPT (ALT)) measured in the serum of miR-122 KO mice. (C) Liver histology at 6 weeks of age, with additional markers for inflammation and immune response, including CD4 and CD8, as well as neutrophils (Ly6g) and apoptosis (TUNEL and cleaved caspase 3). Quantification was performed as number of positive cells per field. *p <0.05, **p <0.01, ***p <0.001, ****p <0.0001. ALP, alkaline phosphatase; KO, knockout; SGOT(AST), serum glutamic-oxaloacetic transaminase; SGPT(ALT), serum glutamic pyruvic transaminase; WT, wild type.

well. We investigated the phenotype of these mice from 2 weeks postnatally onward (Fig. 1). As shown in Fig. 1A, liver inflammation and fibrosis begin at 2 weeks and persist through Week 3. By 6 weeks of age, significant liver inflammation and fibrosis can be found in miR-122 KO mice. At 6 weeks of age, both CD4 and CD8 cells increased in the livers of miR-122 KO mice. The inflammatory process was associated with enhanced macrophage/monocyte liver infiltration and

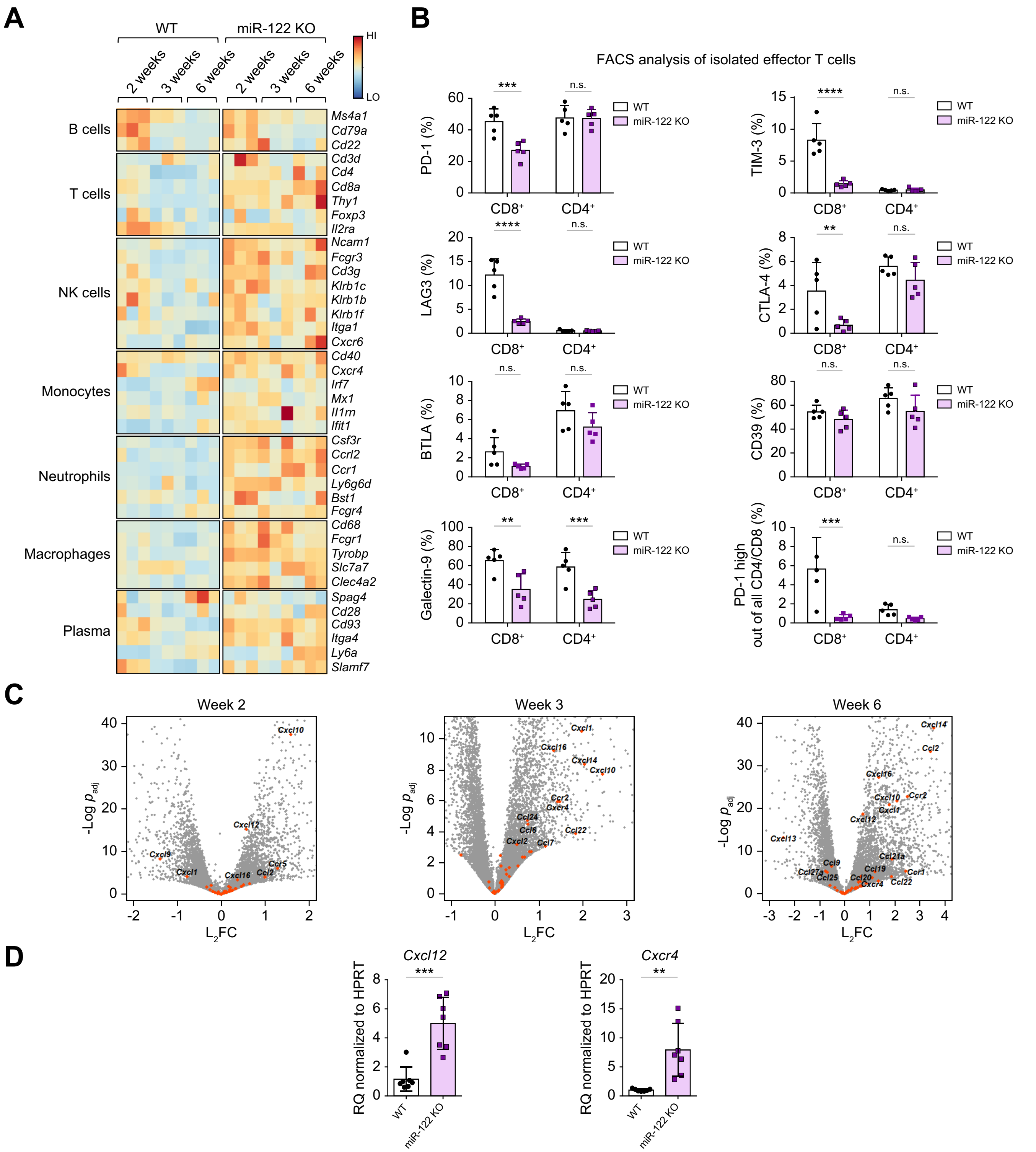

Fig. 2. Enhanced adaptive immune response in miR-122 KO mice. (A) Heatmap of unsupervised genes associated with immune cells in the livers of miR-122 KO mice compared with WT controls at 2, 3, and 6 weeks of age. (B) FACS immunophenotyping analysis of $CD25^{+}$ $CD62L^{-}$ effector T-cell population in immune cells isolated from the livers of miR-122 KO mice compared with WT controls at 6 weeks of age. (C) Volcano plot of differentially expressed genes in miR-122 KO mice compared with WT controls, at 2, 3, and 6 weeks of age. Chemokine-associated genes are indicated in orange. (D) Real-time PCR confirmation of differentially expressed chemokine-associated genes in miR-122 KO mice compared with WT controls, at 6 weeks of age. $^{**}p < 0.01$, $^{***}p < 0.001$, $^{****}p < 0.0001$. KO, knockout; WT, wild type.

increased hepatic neutrophil and lymphocyte count (Fig. 1C), which was also associated with impaired liver function, indicating parenchymal injury (Fig. 1B). This pro-inflammatory, pro-fibrotic effect in the livers of miR-122 KO mice was associated with elevated hepatocyte apoptosis (Fig. 1C).

To further investigate the nature of these pro-inflammatory, pro-fibrotic processes, we performed liver RNAseq over time, which revealed significant overall changes in miR-122 KO livers (Fig. S4D). More than 4,000 genes were significantly differentially expressed (adjusted p <0.05) at the early age of 2

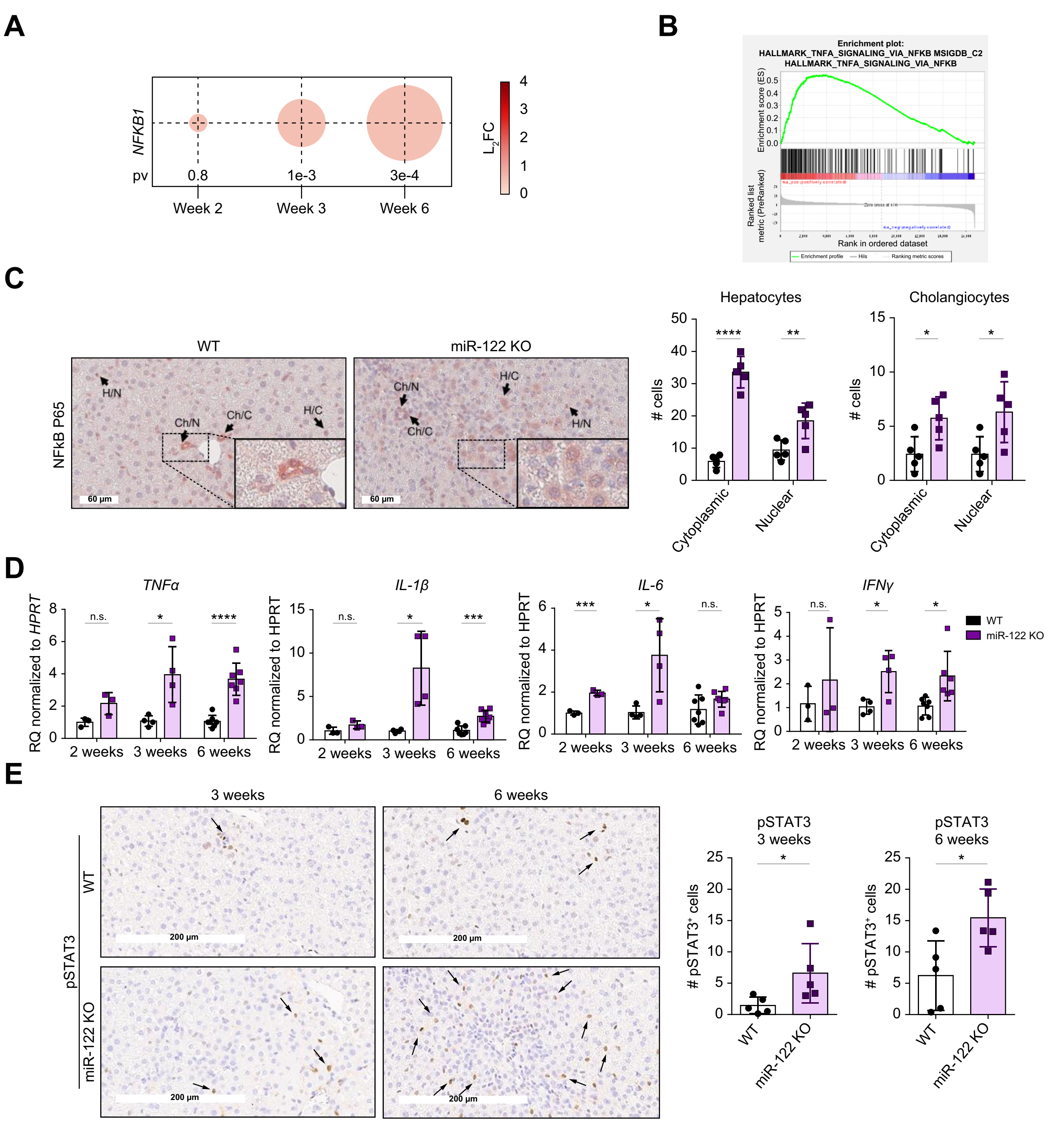

Fig. 3. miR-122 KO mice encounter enhanced immune phenotype. (A) RNAseq gene expression analysis of the livers of miR-122 KO mice compared with WT controls at 2, 3, and 6 weeks of age, showing the significance (pv) and fold change (L2FC) of the NF-κB1 gene encoding the NF-κB protein. (B) GSEA of the TNFα signaling pathway via NF-κB in miR-122 KO mice. (C) NF-κB P65 immunohistochemical staining in miR-122 KO mice and WT controls at 6 weeks of age. Quantification of stained hepatocytes (H) and cholangiocytes (Ch), with nuclear (N) and cytoplasmatic (C) localization. (D) Relative gene expression measured by real-time PCR of downstream mediators of inflammation TNFα, IL-1β, IL-6 and IFNγ, measured in the livers of miR-122 KO mice and WT controls at 2, 3, and 6 weeks of age. (E) pSTAT3 immunohistochemical staining in miR-122 KO mice and WT controls at 3 and 6 weeks of age. *p <0.05, **p <0.01, ***p <0.001, ****p <0.0001. GSEA, gene set enrichment analysis; KO, knockout; RNAseq, RNA sequencing; WT, wild type.

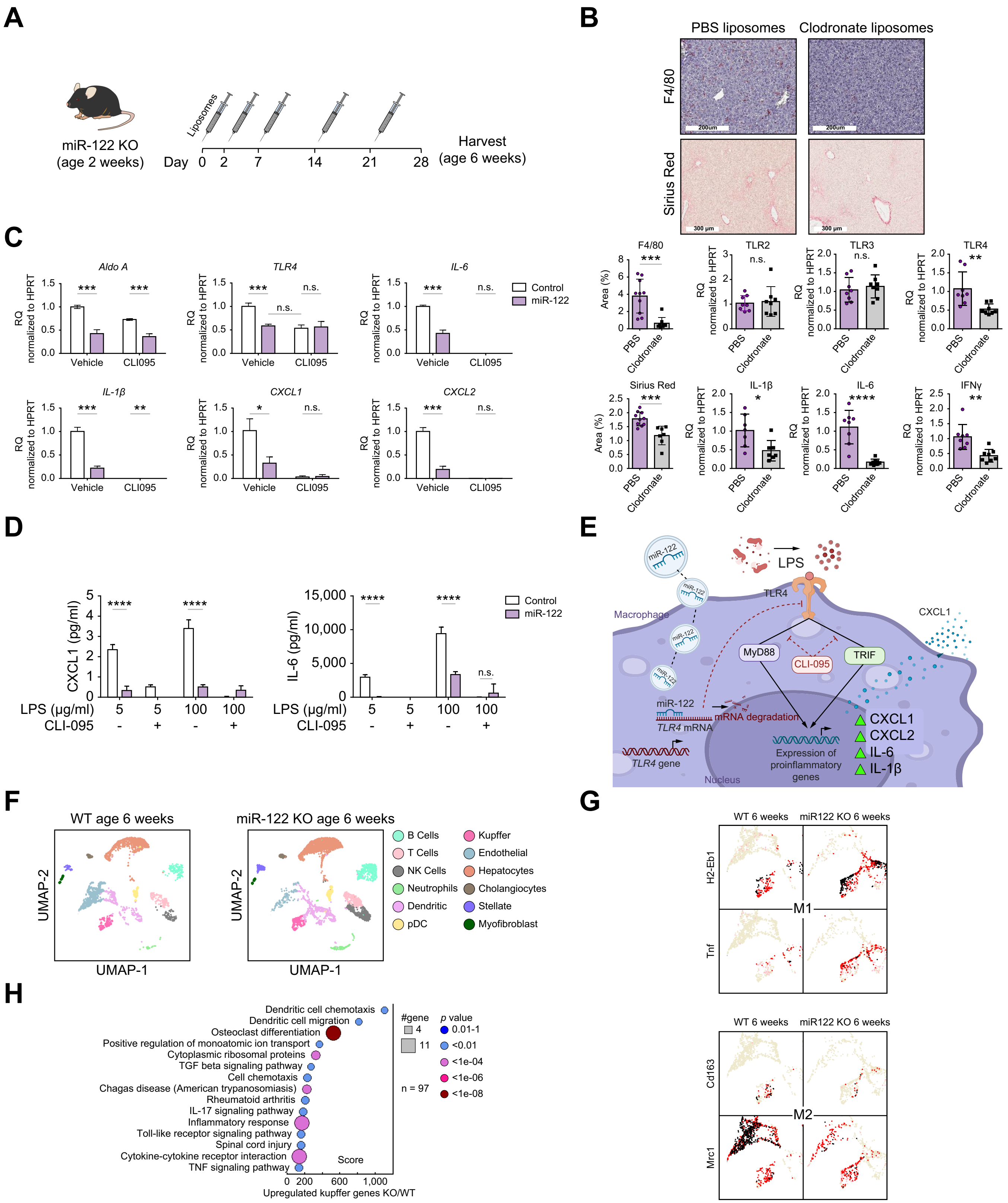

Fig. 4. Immune phenotype in miR-122 KO mice is mediated by macrophages via TLR4. (A) Experimental design: miR-122 KO mice were injected i.p. with liposomal clodronate or liposomal PBS as a control beginning at 2 weeks of age on Days 0, 2, 7, 14, and 21 and harvested on Day 28 at 6 weeks of age. (B) Liver histology of miR-122 KO mice injected with liposomal clodronate or PBS control. Macrophages (F4/80) and fibrosis (Sirius Red). (C) Real-time PCR relative gene expression measured in the livers of miR-122 KO mice injected with liposomal clodronate or PBS control of downstream mediators of inflammation IL-1β, IL-6, and IFNγ, as well as TLR4, TLR2, and TLR3. Experimental design: RAW264.7 murine macrophage cells were transfected with the miR-122 mimic or the scr control. Forty-eight hours after transfection, cells were pretreated with the TLR4 inhibitor CLI-095 as a positive control or with a vehicle (10% DMSO). Cells were activated with LPS

weeks, and approximately 5,000 genes at the 3- and 6-week time points, emphasizing the age-dependent dynamics of the posttranscriptional regulation of genes by miR-122. Of these genes, a significant number were found to regulate fibrosis and the immune response and to be potential direct targets of miR-122 (Table S4). At 35 weeks of age, HCC began to develop in miR-122 KO mice, and by 78 weeks, most mice developed sizable HCC (29/42, size >2mm), consistent with previous studies[15,23] (Fig. S4E). To confirm the efficacy of miR-122 knockout in mice, we assessed the expression of the known miR-122 target gene hemojuvelin (*Hjv*)[14] and its indirect effect on hepcidin (*Hamp*). Both *Hjv* and *Hamp* levels were increased, as determined by reverse-transcription PCR (RT-PCR) and single-cell RNA sequencing (scRNAseq) (Fig. S4F and G), confirming the miR-122 KO functionality.

Role of miR-122 in liver cellular and innate immune responses

In the livers of miR-122 KO mice, we observed significant changes in gene expression (Fig. S4D). Analyzing these changes directed our attention to changes in inflammation/immune cells, both histologically (Fig. 1B,C) and in their gene expression (Fig. 2A). Some of the changes were associated with genes known to be direct targets of miR-122. Changes were apparent in T cells, macrophages/monocytes, B cells, and neutrophils (Fig. 2A). In B cells, an increase in Blnk and CD320 was observed, as indicated by RNAseq; the latter is a known miR-122 target in humans and mice.[24] To further investigate T-cell-related changes, we performed a multiparameter FACS analysis. By assessing immune checkpoint molecules on T cells, we observed a slight reduction in CD4 (Fig. 2B) and a pronounced and significant reduction on CD8 cells, including PD-1, CTLA-4, Galectin-9, TIM-3, and LAG-3 (Fig. 2B). These changes are associated with an enhanced immune activation response of CD8 cells in the liver,[25] as shown in Figs. 1 and 2. In addition to the apparent changes in the cellular immune microenvironment, we observed an overall upregulation of chemokines and chemokine receptors, as examined by RNAseq, which may also contribute to the increase in immune and inflammatory cell migration into the liver[26] and to liver fibrosis, including CXCL12 and CXCR4[27] (Fig. 2C,D). To further understand the changes in the functionality of CD8 cells, we performed scRNAseq of CD8 cells from 2- and 6-week-old miR-122 KO and WT mice. We identified increased CD8 T-cell receptor (TCR) signaling and IL-12 signaling (Fig. S5A). This observation recapitulates the association between miR-122 and IL-12a (Fig. S5B and C) in human livers, indicating immune activation of the liver in miR-122 KO mice. Moreover, increased expression of IL-12A ($\log_2$ fold change 3.041, p = 6.6E-20) and its receptor IL-12RB1 was found in B cells, and the expression of IL-12RB2 was found in T, NK, and NK-T cells, as shown in scRNAseq clustering (Fig. S5D), underlying the communication between these immune cells. MiR-122 KO mice show an increase in ALP without an increase in hepatocellular enzymes, in line with a strong upregulation of the Alpl transcript encoding the ALP enzyme, and with the known fact that Alpl is a direct miR-122 target (in both mice and humans). In addition, miR-122 KO mice already exhibit a ductular reaction at an early age, with enhanced CK19 expression (data not shown). ALP is associated with the development of ductular reaction in both mice and humans.[28]

In addition to immune/inflammatory cellular activation, it is important to determine whether the innate immune system participates in the immune activation of the livers of miR-122 KO mice. A gene enrichment analysis (Fig. 3A,B) showed increased TNFα signaling, including enhanced NF-κB transcript levels. Cellular and nuclear expression of p65 (transducing NF-κB signaling) is increased in hepatocytes and cholangiocytes of miR-122 KO mice (Fig. 3C). The enhanced nuclear RelA signal is associated with an overall increase in TNFα, IL-1β, and IL-6 expression (Fig. 3D), paralleling the expression of IFNγ (Fig. 3D). TNFα and IL-1β activate NF-κB signaling, and downstream of IL-6, pSTAT3 activation can be observed, both enhanced in liver parenchymal and non-parenchymal cells (Fig. 3C,E). pSTAT3 levels are increased at both the 3- and 6-week time points, supporting our hypothesis that the lack of miR-122 leads to increased IL-6 and its activated pathways, as well as the enhancement of other potential inflammatory signals. Furthermore, IL6 shows an increase at 2 and 3 weeks in the liver of miR-122 KO mice (Fig. 3D). IL-6 plays a central role in liver physiology, particularly in both regeneration and immune regulation.[29] MiR-122, produced by hepatocytes, is secreted into the liver microenvironment,[8] targeting numerous immune mediators on adjacent macrophages, including TLR2 and TLR4.[30] In addition, as shown in this study, it also indirectly targets TLR3. The signaling downstream of these TLRs is attenuated in miR-122-proficient mice, and upon miR-122 elimination, this arm of the innate immune response is enhanced. This observation also explains the enhanced inflammation and fibrosis phenotype we observed in Fig. 1A,C. In addition, IFNγ enhancement is associated with liver inflammation in immune-mediated liver disease, such as primary sclerosing cholangitis.[31] This potentially suggests that upon elimination of miR-122, a significant immune imbalance develops in the liver.

Regulation of TLR3 by miR-122

TLRs are important immune sensors of invading pathogens, and TNFα plays an important role in one of their effector pathways.[32] The microbiota is an important constituent player in the gut–liver axis,[33] and a non-functional gastrointestinal

at two doses (5 or 100 ng/ml). Twenty-four hours after activation, medium and cells were collected for analysis. Real-time PCR relative gene expression measured in cells transfected with the miR-122 mimic compared with the scr control, treated with LPS (100 ng/ml). AldoA (positive control and a known target of miR-122), as well as TLR4 and the downstream mediators of inflammation IL-6, IL-1β, CXCL1, and CXCL2. (D) Secretion of the proinflammatory markers CXCL1 and IL-6 into the medium was measured by ELISA. (E) Scheme of the proposed function of miR-122 on TLR4 signaling in macrophages. (F) ScRNAseq data generated from cells isolated from the livers of miR-122 KO mice and WT controls at 6 weeks of age, sorted into clusters. (G) ScRNAseq analysis of the livers of miR-122 KO mice and WT controls at 6 weeks of age, indicating the clusters of cells expressing M1 and M2 markers. (H) Pathway analysis for KO Kupffer *vs*. WT Kupffer from scRNAseq samples. DEGs are selected with adjusted p value <0.02 and applied to over-representation analysis using the following libraries: WikiPathways_2024_Mouse, KEGG_2019_Mouse, GO_Biological_Process_2025. *p <0.05, **p <0.01, ***p <0.001, ****p <0.0001. DEG, differentially expressed gene; KO, knockout; LPS, lipopolysaccharide; RNAseq, RNA sequencing; scRNAseq, single-cell RNA sequencing; TLR, Toll-like receptor; WT, wild type.

barrier leads to an elevated transmigration of bacteria into the circulation. Interestingly, it was recently reported that miR-122 directly regulates TLR4,[34] the immune sensor for bacteria. Upon investigating a potential factor to increase TLR3, we focused on IRF2 (Fig. S3F–H). IRF2 is a known TLR3 transcriptional factor,[35] with (i) a high binding score to the TLR3 promoter, (ii) a high Amadeus *p* value score of 7.8E-28 (Fig. S3F), and (iii) a high miR target binding score to IRF2 (by genie.weizmann.ac.il -15.33). To prove the direct effect of miR-122 on IRF2, we constructed a cellular luciferase assay system, in which, upon miR-122 transfection, human IRF2 was significantly reduced. Mutating the miR-122 seed sequence present in the 3′UTR of IRF2 abolished this miR-122 effect (Fig. S3I). Furthermore, injection of antagomir-122 into mice led to increased IRF2 expression (Fig. S3J and K). Thus, immune sensors for both viral and bacterial infections are regulated by miR-122, as shown for TLR2 and TLR4 (in other studies) and for TLR3 (in this study) (see the schematic description of IRF2 and TLR3 expression in Fig. S3L). In the transcriptomic investigation, TLR3 expression was significantly upregulated, with a 2.19-fold change (adjusted $p = 0.013$). IRF2 expression was also significantly upregulated, with a 1.81-fold change (adjusted $p = 0.009$).

Macrophage activation in miR-122 KO mice

The observation of an enhanced macrophage liver population associated with enhanced fibrosis (Fig. 1A) in miR-122 KO mice, together with innate immune activation and TLR expression, led us to hypothesize that the liver macrophage population in miR-122 KO mice could be a cellular culprit for many of the observed phenotypes and changes in immune signaling. To determine the role of macrophages in miR-122 KO mice, we performed an *in vivo* macrophage depletion experiment (Fig. 4A). We repeatedly administered liposomal clodronate to young mice, which efficiently eliminated macrophages as well as significantly reduced liver fibrosis (Fig. 4B), indicating the importance of macrophages in fibrosis development. However, as shown in Fig. 4B, eliminating macrophages from 2-week-old mice and analyzing them at 6 weeks revealed that fibrosis remains very apparent, suggesting independent pathways linking fibrosis and macrophage activation in the liver. This was associated with a significant reduction in TLR4, as well as in IL-1β, IL-6, and IFNγ, but not in TLR2 and TLR3 (Fig. 4B). To determine the specificity of the effects of miR-122 on macrophages, we performed an *in vitro* macrophage activation experiment. A macrophage cell line was transfected with the miR-122 mimic or a negative control, in the presence or absence of the TLR4 inhibitor CLI-095, and then treated with low-dose LPS (5 ng/ml) and high-dose LPS (100 ng/ml), thereby mimicking TLR4 activation by bacterial infection. As shown in Fig. 4C, miR-122 specifically suppressed TLR4 as well as IL-6, IL-1β, CXCL1, and CXCL2, all of which are known to be involved in liver fibrosis (the suppression of AldoA indicates miR-122 activity, as Aldo A is a known target of miR-122). Furthermore, miR-122 suppressed the secretion of CXCL1 and IL-6 (Fig. 4D). The model we propose for this experiment is shown in Fig. 4E. To determine whether the liver macrophage/immune change in phenotype is associated with M1 or M2, we analyzed the scRNAseq data. Our findings reveal an increase in the M1 phenotype in the livers of miR-122 KO mice and a decrease in the M2 phenotype, as shown in Fig. 4F–H. These changes also include endothelial cells in the same direction.

Role of CD4 and CD8 cells in the miR-122 KO mice

To determine whether the inflammation and fibrosis observed in the miR-122 KO mice depend on CD4 and CD8 cells, we treated the mice with a combination of anti-CD4 and anti-CD8 antibodies to deplete both cell types (Fig. S6A). This treatment significantly reduced both T-cell subsets, as shown by CD3 staining (Fig. S6B). As shown in Fig. S6C, CD8 cells were significantly depleted, as confirmed by PCR analysis, corroborating efficient treatment. The depletion of both CD4 and CD8 cells did not affect fibrosis or inflammatory signaling pathways in miR-122 KO mice. The results from Sirius Red staining indicate no significant difference after depleting both CD4 and CD8 cells. In addition, the combination treatment showed no effect (Fig. S6B). The analysis of αSMA message further confirms that depleting CD4 and CD8 cells had no effect on fibrosis (Fig. S6C).

Liver autoimmunity

The hepatic infiltration of immune cells (Figs. 1–4) and enhanced TLR expression led us to speculate that chronic autoimmune hepatitis could develop in these miR-122 KO mice. Furthermore, miR-122 KO mice exhibit ductular reaction.[36] To identify the pivotal pathways relevant to the immune privilege of the liver microenvironment, we compared the RNAseq data of patients with AIH with those of miR-122 KO mice, owing to the similarities we observed (Fig. 5A). The RNAseq data of 13 patients with AIH were scored using the modified Hepatitis Activity Index (mHAI) system,[37] which enabled us to categorize the patients into two groups: the low-mHAI (<6) and high-mHAI (>9) score patients (Fig. 5B,C). The high-mHAI patients had an enhanced NF-κB signaling pathway as well as an enhanced TLR pathway (Fig. 5D). Liver miR-122 levels negatively correlated with the mHAI score (Fig. 5E). Following this human and mouse analysis, we learned that TLR signaling is predominant in human AIH (Fig. 5D). In the RNA expression data of the 2-, 3-, and 6-week-old miR-122 KO mice, we observed, at most time points, a significant increase in TLR2, TLR3, and TLR4 (Fig. 5F). MiR-122, although expressed in hepatocytes, is secreted and also affects other cell types, such as Kupffer cells and macrophages, possibly explaining the overall increase in the expression of these three TLRs in miR-122 KO mice. TLR4 is also associated with liver fibrosis[38] and is a target of miR-122.[34] To determine the functionality of enhanced TLR4 expression, we treated the miR-122 KO mice with a mixture of oral antibiotics, assuming that gut microbiome signaling occurs through hepatic TLR4, as previously reported[39] (Fig. S7A and B). This antibiotic treatment reduced liver macrophages (although not significantly), liver fibrosis (Fig. S7C), and hepatic expression of TNFα, IL-1β, IFNγ, IL-12b (IL-12a and IL-6 were reduced, but not significantly), and TLR4 (Fig. S7D). This suggests that a mechanism of enhanced liver fibrosis is associated with the gut microbiome–TLR4 liver axis. The inflammatory state of these mice was also evidenced by increased serum amyloid A1 (SAA1) and SAA2 RNA levels at 2, 3, and 6 weeks (Fig. S8A and B). At the protein level, the SAA measured in the serum of miR-122 KO mice did not reach statistical difference (Fig. S8C), but the SAA in the blood of miR-

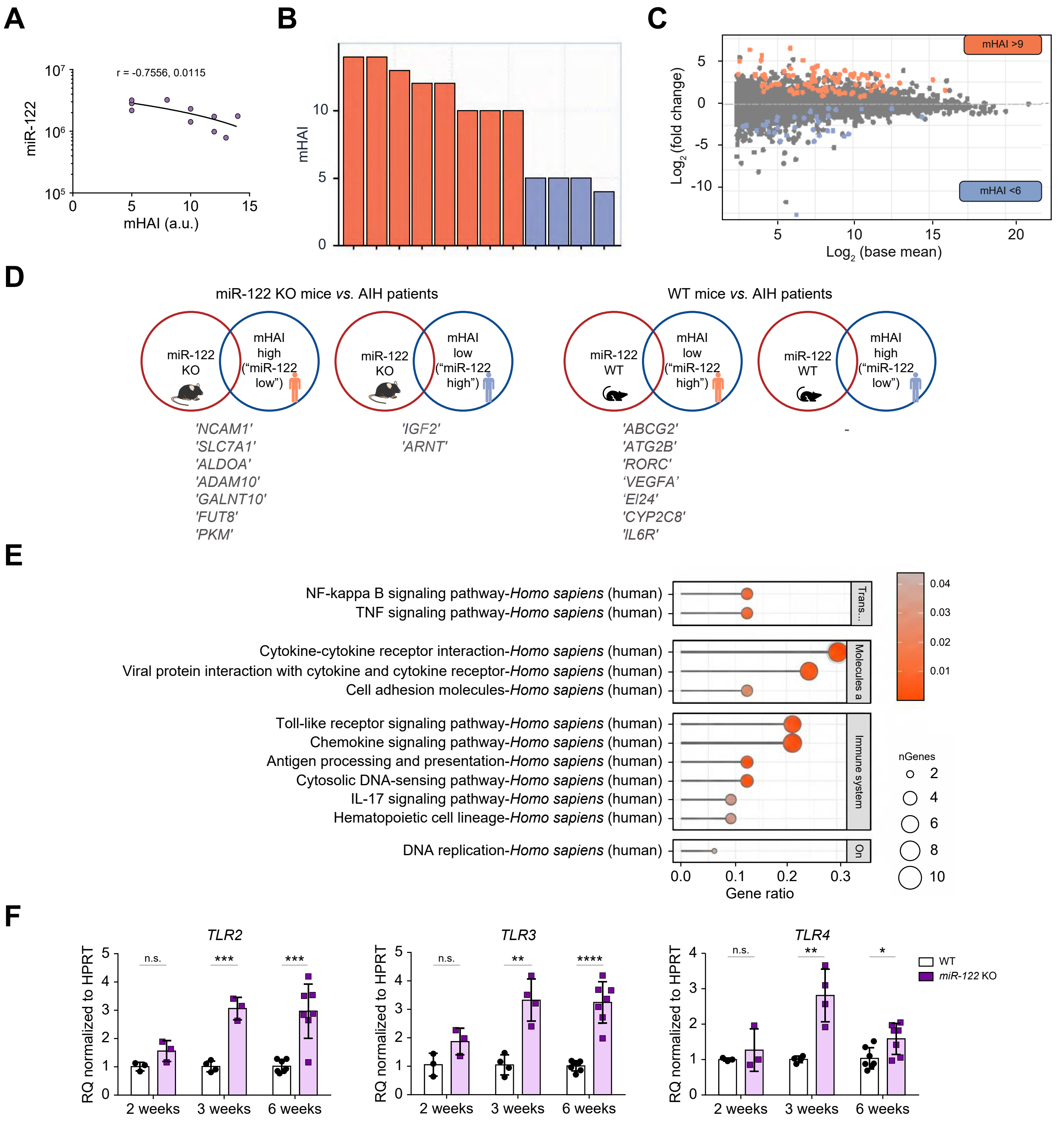

Fig. 5. Low expression of miR-122 in patients with AIH induces TLR signaling upregulation. (A) Spearman correlation between miR-122 expression in liver samples and mHAI score in patients with AIH. (B) Patients with AIH grouped into high-mHAI (orange) and low-mHAI (blue) individuals. (C) RNAseq gene expression analysis of liver samples from patients with AIH: MA plot of differential gene expression between high- and low-mHAI patients with AIH. (D) Gene set enrichment analysis indicating pathways upregulated in the livers of high-mHAI patients with AIH. (E) Overlap between overexpressed genes in bulk mRNA-sequencing data from liver samples of high- and low-mHAI patient groups and bulk mRNA-sequencing data from the livers of miR-122 KO mice and WT controls at 6 weeks of age. (F) Real-time PCR confirmation of expression of TLR2, TLR3, and TLR4, measured in the livers of miR-122 KO mice and WT controls at 2, 3, and 6 weeks of age. *p <0.05, **p <0.01, ***p <0.001, ****p <0.0001. AIH, autoimmune hepatitis; KO, knockout; mHAI, modified Hepatitis Activity Index; RNAseq, RNA sequencing; TLR, Toll-like receptor; WT, wild type.

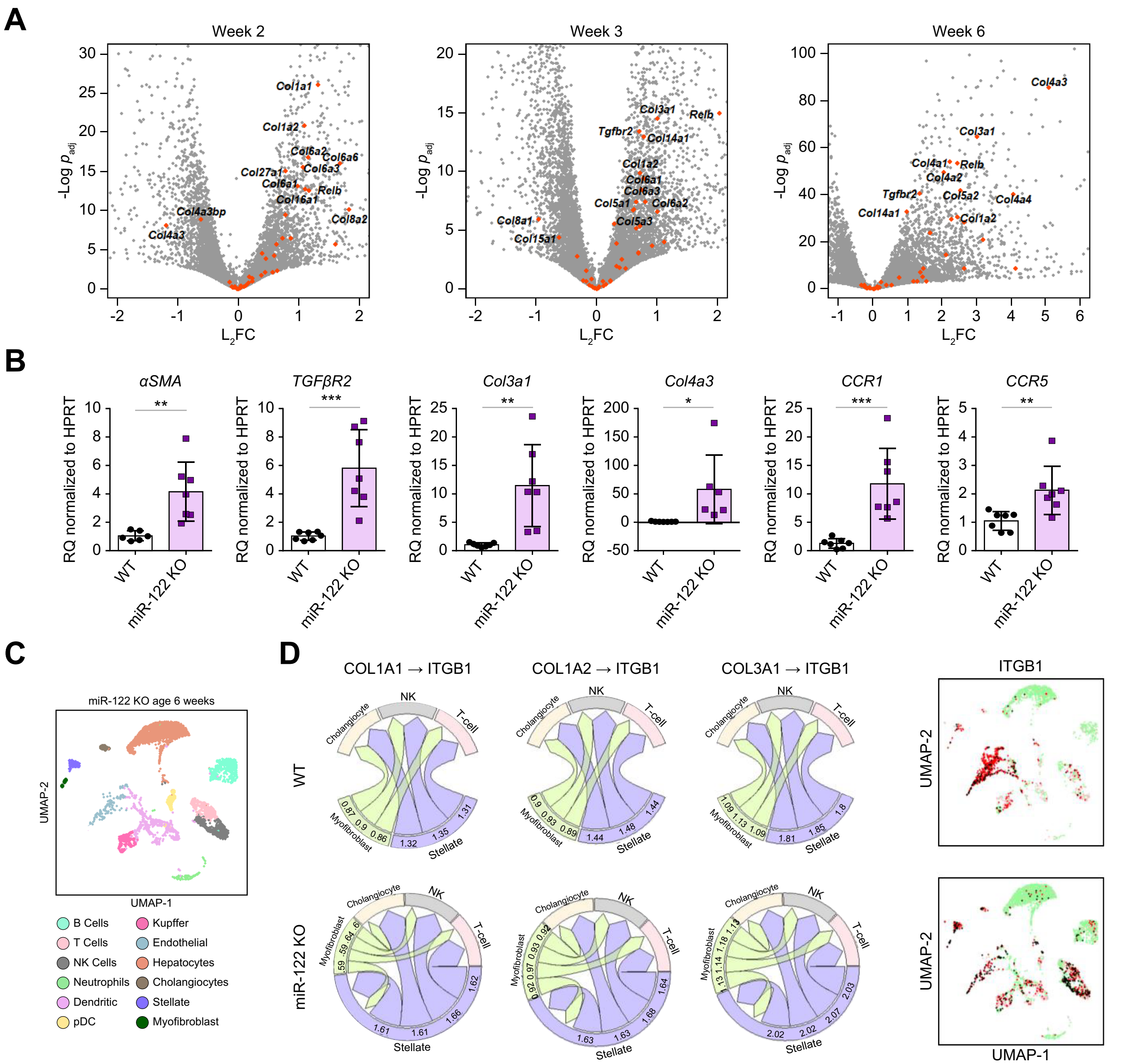

Fig. 6. Increased fibrosis in miR-122 KO mice. (A) Volcano plot of differentially expressed genes in miR-122 KO mice compared with WT controls, at 2, 3, and 6 weeks of age. Genes associated with fibrosis are indicated in orange. (B) Real-time PCR confirmation of differentially expressed fibrosis-related genes at 6 weeks of age. (C) ScRNAseq data sorted into clusters with legend. (D) CellPhoneDB collagen (COL1A1, COL1A2, and COL3A1) and integrin α2β1 (ITGB1) interactions associated with fibrosis from scRNAseq data: interaction analysis of myofibroblasts (green) and stellate cells (purple) with cholangyocytes (yellow), NK cells (gray), and T cells (soft pink). Interaction expression scores are indicated on the plot and represented by the width of the ribbon. *p <0.05, **p <0.01, ***p <0.001. KO, knockout; scRNAseq, single-cell RNA sequencing; WT, wild type.

122 KO mice was increased. This increase was mainly observed in hepatocytes, as indicated by scRNAseq data (Fig. S8D).

Liver fibrosis is miR-122 dependent

Liver fibrosis is associated with chronic liver diseases; however, both are mechanistically not fully deciphered and have no effective therapeutic treatment.[40] We investigated the effect of miR-122 on liver fibrosis because of the observation that in the miR-122 KO model, fibrosis co-occurs with inflammation rather than occurring in a secondary manner. The phenotypic change of enhanced liver fibrosis in these miR-122 KO mice was apparent shortly after birth (Fig. 1A), once the gut microbiome was established. This led us to further investigate the role of miR-122 in liver fibrosis using RNAseq and real-time PCR analyses (Fig. 6A,B). We observed increased αSMA expression in the livers of these miR-122 KO mice already at an early age (Fig. 6B). In addition, upon examining the RNAseq

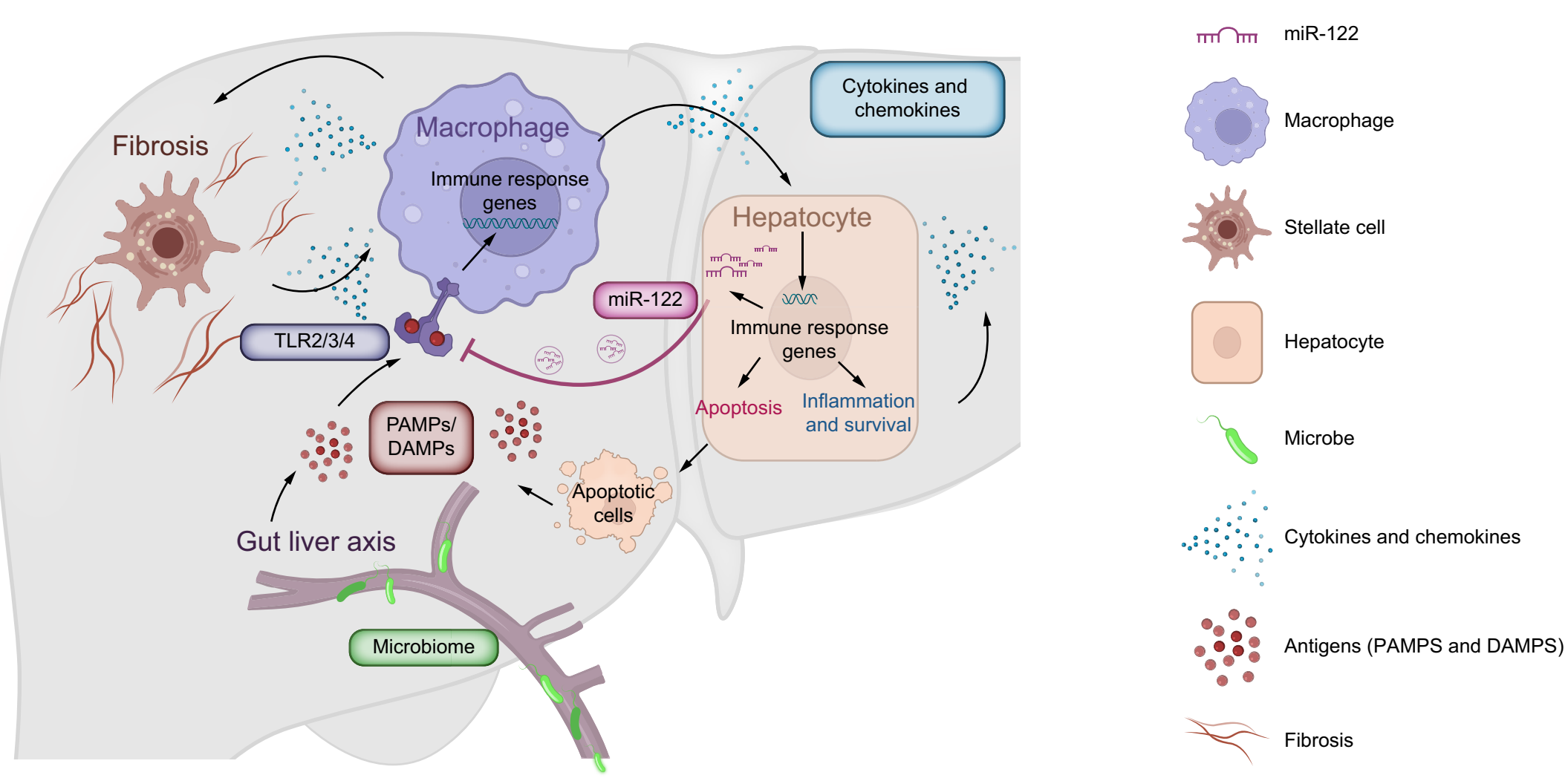

Fig. 7. Graphical representation of the role of miR-122 in the cellular crosstalk of liver immune regulation.

data of the miR-122 KO mice, we observed an increase in the expression of collagen genes, which is most apparent at Week 6 of age (Fig. 6A). TGFβR2, the dominant TGFβR, is a target of miR-122[41] and is also increased, as depicted in a volcano plot and confirmed by RT-PCR (Fig. 6A,B). This increase in collagen genes is further supported by an analysis of ligand–receptor interactions derived from scRNAseq data[42] (Fig. 6C,D). The robustness of the increase in collagen expression in interaction with the receptor is most apparent in these miR-122 KO mice. To further assess the presence of pro-fibrotic effectors, we investigated two chemokine receptors, CCR1 and CCR5,[43,44] which are associated with liver fibrosis and are both increased in miR-122 KO mice (Fig. 6B). In these KO mice, the target of these collagens, integrin α2β1, is enhanced (Fig. 6D). These observations fit with the overall effect of miR-122 in suppressing liver fibrosis, which probably occurs through a direct effect on several pro-fibrotic mediators, in which miR-122 suppresses their expression and thus modifies the inflammatory pro-fibrotic stimuli.

Osteopontin (SPP1) is increased in many chronic liver diseases[45] and is associated with liver fibrosis. Furthermore, SPP1 is associated with clinical conditions identified in a mouse model of inflammation and fibrosis. SPP1, associated with ductular reaction and fibrosis, was significantly increased in the miR-122 KO mice (Fig. S9A). This increase was apparent in 2-, 3-, and 6-week-old miR-122 KO mice, as shown by RNAseq (Fig. S9B) and confirmed by PCR in the 6-week-old mice (Fig. S9C). This increase in SPP1 transcripts levels is primarily observed in the hepatocyte population (Fig. S9D).

Discussion

The regulation of liver tolerance, inflammation, and fibrosis is central to many important clinical pathologies, both hepatic and systemic. These include liver cancer, metastasis, and infections caused by viruses, bacteria, and parasitic pathogens with hepatotropism, such as hepatitis viruses, which thrive in the liver. Autoimmune liver diseases and liver transplantation are managed with immune suppression drugs, underscoring the pivotal role of liver immune regulation. However, our understanding of the regulation of the liver immune microenvironment is limited. This gap hampers the development of therapeutics for various diseases. Previous studies and our preliminary clinical observations indicate that autoimmune liver diseases are associated with low liver miR-122 levels. This led us to propose that low miR-122 levels in the liver result in a breach in liver immune tolerance. MiR-122 is a liver-specific microRNA secreted by hepatocytes in exosomes, enabling its delivery to other liver cell types, including macrophages, endothelial cells, stellate cells, and immune cells, as well as to other organs in the body that are distant from the liver, through the circulation, as we reported for the kidney, adipose tissue, and muscle.[8] This microRNA is unique because it is produced in very high levels in each hepatocyte (>100,000 copies/hepatocyte). To understand the role of miR-122, we used several techniques, including administration of miR-122 mimics and antagomir-122, and the generation of miR-122 KO mice. In our study, the excised genomic region was small (59 nt), unlike in previous miR-122 KO mice, which had a significantly larger genomic region deleted, possibly causing off-target effects. Moreover, unlike earlier clinical observations of miR-122 KO mice,[15,23] which assessed the development of liver inflammation, steatosis, and HCC,[12,13] we were interested in determining the alterations occurring in the liver at an early age, as we assumed that changes in this early phase would have major significance. To recapitulate the previous findings reported in miR-122 KO mice, our observations align with these reports. We confirmed that our miR-122 KO mice, which suffer from fibrosis and inflammation at an early age, almost all develop primary liver cancers at a later stage. We believe that this phenomenon is significant, potentially demonstrating that reduced miR-122 levels in human liver inflammatory states contribute to early liver fibrosis and inflammation, which culminate after several years in primary liver cancer. The reported observations that miR-122 is a direct tumor suppressor, increases p53, and is a negative regulator of fibrosis and

inflammation could position miR-122 at the forefront of future therapeutic targets for chronic inflammatory liver diseases and liver cancers.

The liver is continually exposed to the gut microbiome, which includes bacteria, fungi, and viruses. Our research shows that reducing the gut bacterial load in miR-122 KO mice suppresses liver inflammation and attenuates macrophage activation (Fig. S7). This suggests that miR-122 regulates the pro-inflammatory part of the gut–liver axis. This regulation by miR-122 of the pro-inflammatory axis of the gut–liver effect may occur through negative control of TLR2, TLR3, and TLR4. This is potentially significant in attenuating the activation of liver inflammation by the gut microbiome, which flushes through the portal vein into the liver.

By using early-age mice and multiple approaches, we identified the multilayered immune effects of miR-122. The reduction of this microRNA enhances innate immune responses in the liver. The miR-122 mimic reversed the immune phenotype, thereby enabling tolerance. MiR-122 KO mice exhibited an increased innate immune response in the liver. Eradication of miR-122 increased the homing effects of chemokines and chemokine receptors on immune cells to migrate to the liver. These observations were associated with the simultaneous initiation of several pro-fibrotic pathways, leading to very early fibrosis in the miR-122 KO mice, beginning at weeks 2–3 of life. Liver macrophages are central to these inflammatory phenotypes, as demonstrated by our *in vitro* and *in vivo* studies. Recent reports have highlighted some interesting potential mediators of liver fibrosis in miR-122 KO mice: Bmp8 and Ihh (Indian hedgehog). They are both significantly upregulated in the miR-122 KO liver and have been bioinformatically defined as direct targets of miR-122 (Table S4). Bmp8 has been shown to promote a pro-inflammatory phenotype in hepatic stellate cells by signaling through both the SMAD2/3 and SMAD1/5/9 branches of the TGFβ/BMP pathway and may also contribute to the fibrotic phenotype of the miR-122 KO liver. Ihh encodes a ligand of the Hedgehog signaling, whose activation in cholangiocytes and liver stromal cells induces inflammatory and fibrogenic wound-healing responses. Recently, it was shown that macrophage β–catenin–Ihh axis induces stellate cell activation and fibrosis. Periostin promotes Ihh expression, which, in turn, promotes hepatic stellate cell activation, also contributing to the fibrotic phenotype of miR-122 KO mice. Altogether, it seems that miR-122 is a central regulator of liver fibrosis. Currently, with no approved drug to prevent or reduce liver fibrosis, miR-122 regulation could be proposed as a target to modulate liver fibrosis (Fig. 7).

Our current understanding, based on these results, is that reduced levels of miR-122 in the liver during chronic liver inflammatory states, as many previous studies have shown, lead to liver inflammation and fibrosis through independent pathways. This could explain the co-occurrence of both pathologies in many chronic liver clinical conditions.

Affiliations

[1]*Goldyne Savad Institute of Gene Therapy, Hadassah Hebrew University Hospital, Jerusalem, Israel;* [2]*Protozoa Immunology, Bernhard Nocht Institute for Tropical Medicine, Hamburg, Germany;* [3]*University Medical Centre Hamburg-Eppendorf, Hamburg, Germany;* [4]*The Wohl Institute for Translational Medicine, Human Biology Research Center, Hadassah University Medical Center, Jerusalem, Israel;* [5]*Computation Center, The Hebrew University of Jerusalem, Jerusalem, Israel;* [6]*Institute for Inflammation and Carcinogenesis, Hamburg Center for Translational Immunology, University Medical Center Hamburg-Eppendorf, Hamburg, Germany;* [7]*Liver institute Hadassah Hebrew University Hospital, Jerusalem, Israel;* [8]*Department of Surgery Hadassah Hebrew University Hospital, Jerusalem, Israel;* [9]*Division of Chronic Inflammation and Cancer, German Cancer Research Centre Heidelberg (DKFZ), Heidelberg, Germany;* [10]*The M3 Research Center, Medical Faculty, University Clinic Tübingen (UKT), Tübingen, Germany;* [11]*Department of Biochemistry, University of Kiel, Kiel, Germany*

Abbreviations

αSMA, alpha-smooth muscle actin; AIH, autoimmune hepatitis; CCR1, C–C chemokine receptor type 1; CCR5, C–C chemokine receptor type 5; CD4, cluster of differentiation 4; CD8, cluster of differentiation 8; CTLA-4, cytotoxic T-lymphocyte-associated protein 4; CXCL1, C-X-C motif chemokine ligand 1; CXCL12, C-X-C motif chemokine ligand 12; CXCL2: C-X-C motif chemokine ligand 2; CXCR4: C-X-C motif chemokine receptor 4; GSEA, gene set enrichment analysis; HCC, hepatocellular carcinoma; HLA, human leukocyte antigen; IFNγ, interferon gamma; IRF2, interferon regulatory factor 2; KEGG, Kyoto Encyclopedia of Genes and Genomes; KO knockout; LAG-3, lymphocyte activation gene-3; LPS, lipopolysaccharide; mHAI, modified Hepatitis Activity Index; miR-122, microRNA-122898/21; PD-1, programmed death-1; pSTAT3, phosphorylated signal transducer and activator of transcription 3; RNAseq, RNA sequencing; RT-PCR, reverse transcription PCR; SAA, serum amyloid A; scRNAseq, single-cell RNA sequencing; SPP1, secreted phosphoprotein 1 (also known as osteopontin); TCR, T-cell receptor; TGFR, transforming growth factor receptor; TGFβR2, transforming growth factor-beta receptor 2; TIM-3, T-cell immunoglobulin and mucin domain-containing protein 3; TLR, Toll-like receptor; TNFα, tumor necrosis factor-alpha; WT, wild type.

Financial support

This work was supported by the National Institutes of Health grant CA197081-02 (to EG); MOST–DKFZ grant 4037 (to EG); the ISF collaboration with Canada grant (2473/2017) (to EG); the personal ISF grant (486/2017) (to EG); the ISF IPMP grant (3751/21) (to EG); the Deutsche Forschungsgemeinschaft, Bonn, Germany, grant (SFB841) (to EG); the ERC Advanced Grant No. 786575—RxmiRcanceR, including the PanRORa POC 101188783 (to EG); the HFSP RGP0011/2022 grant (to EG); the Kron, Raskin, and Einstein grant (A-2021-646) (to EG); the Robert Benson Foundations grant (to EG); and the Kamea Scientific Foundation of the Israeli Government (to DSG). JH and EG were also supported by the DFG grant 544225277

Conflicts of interest

The authors declare that they have no competing interests.

Please refer to the accompanying ICMJE disclosure forms for further details.

Authors' contributions

Conceptualization: MG, ZS, SRJ, MH, HG, EG. Methodology: MG, SL, EK, ZS, JB, TJ, LA, CK, MO, OG, NA, RA, YN, SE, HB, JM, EZK, NR, AM, DY, ASY, AS, MR, OA, DSG, AP, JA, JR, SH, NG, CSc, JHer, AC,RS, AH, AI, DH, JHet, SRJ, MH, HG, EG. Investigation: all authors. Visualization: MG, SL, EK, ZS, NA, RA. Funding acquisition: MH, EG. Project administration: None. Supervision: MH, HG, EG. Writing—original draft: MG, EG. Writing—review & editing: MH, DSG, HG, EG.

Data availability

All data relating to the paper are deposited in the GEO public database (accession number GSE278824).

Acknowledgements

We thank Ms Meital Guy Cohan, MSc, for the help with the administrative work. We thank the animal farm technicians for their valuable help and the animal farm ethics committees in both Israel and Germany for reviewing our numerous requests. We thank all the patients who gave their informed consent, data, and samples for this investigation. We also thank the IRBs in both Israel and Germany for reviewing our requests.

Supplementary data

Supplementary data to this article can be found online at https://doi.org/10.1016/j.jhepr.2026.102020.

References

Author names in bold designate shared co-first authorship.

[1] Davidson A, Diamond B. Autoimmune diseases. N Engl J Med 2001;345:340–350.
[2] Trivedi PJ, Hirschfield GM. Recent advances in clinical practice: epidemiology of autoimmune liver diseases. Gut 2021;70:1989–2003.
[3] Theofilopoulos AN, Kono DH, Baccala R. The multiple pathways to autoimmunity. Nat Immunol 2017;18:716–724.
[4] Herkel J, Carambia A, Lohse AW. Autoimmune hepatitis: possible triggers, potential treatments. J Hepatol 2020;73:446–448.
[5] Kubes P, Jenne C. Immune responses in the liver. Annu Rev Immunol 2018;36:247–277.
[6] Bartel DP. Metazoan microRNAs. Cell 2018;173:20–51.
[7] Bandiera S, Pfeffer S, Baumert TF, et al. miR-122—a key factor and therapeutic target in liver disease. J Hepatol 2015;62:448–457.
[8] Rivkin M, Simerzin A, Zorde-Khvalevsky E, et al. Inflammation-induced expression and secretion of microRNA 122 leads to reduced blood levels of kidney-derived erythropoietin and anemia. Gastroenterology 2016;151:999–1010.e3.
[9] Tiegs G, Horst AK. TNF in the liver: targeting a central player in inflammation. Semin Immunopathol 2022;44:445–459.
[10] Lavon I, Goldberg I, Amit S, et al. High susceptibility to bacterial infection, but no liver dysfunction, in mice compromised for hepatocyte NF-κB activation. Nat Med 2000;6:573–577.
[11] Pikarsky E, Porat RM, Stein I, et al. NF-κB functions as a tumour promoter in inflammation-associated cancer. Nature 2004;431:461–466.
[12] He B, He Y, Shi W, et al. Bioinformatics analysis of gene expression alterations in microRNA-122 knockout mice with hepatocellular carcinoma. Mol Med Rep 2017;15:3681–3689.
[13] Nakao K, Miyaaki H, Ichikawa T. Antitumor function of microRNA-122 against hepatocellular carcinoma. J Gastroenterol 2014;49:589–593.
[14] Castoldi M, Vujic Spasic M, Altamura S, et al. The liver-specific microRNA miR-122 controls systemic iron homeostasis in mice. J Clin Invest 2011;121:1386–1396.
[15] Tsai W-C, Hsu S-D, Hsu C-S, et al. MicroRNA-122 plays a critical role in liver homeostasis and hepatocarcinogenesis. J Clin Invest 2012;122:2884–2897.
[16] Koyama Y, Brenner DA. Liver inflammation and fibrosis. J Clin Invest 2017;127:55–64.
[17] Wen J, Friedman JR. miR-122 regulates hepatic lipid metabolism and tumor suppression. J Clin Invest 2012;122:2773–2776.
[18] Simerzin A, Zorde-Khvalevsky E, Rivkin M, et al. The liver-specific microRNA-122*, the complementary strand of microRNA-122, acts as a tumor suppressor by modulating the p53/mouse double minute 2 homolog circuitry. Hepatology 2016;64:1623–1636.
[19] Mieli-Vergani G, Vergani D, Czaja AJ, et al. Autoimmune hepatitis. Nat Rev Dis Prim 2018;4:18017.
[20] Müller A-L, Casar C, Preti M, et al. Inflammatory type 2 conventional dendritic cells contribute to murine and human cholangitis. J Hepatol 2022;77:1532–1544.
[21] Colasanti O, Burm R, Huang H-E, et al. Comparison of HAV and HCV infections in vivo and in vitro reveals distinct patterns of innate immune evasion and activation. J Hepatol 2023;79:645–656.
[22] Ahearn-Ford S, Berrington JE, Stewart CJ. Development of the gut microbiome in early life. Exp Physiol 2022;107:415–421.
[23] Hsu S-H, Wang B, Kota J, et al. Essential metabolic, anti-inflammatory, and anti-tumorigenic functions of miR-122 in liver. J Clin Invest 2012;122:2871–2883.
[24] Elmén J, Lindow M, Silahtaroglu A, et al. Antagonism of microRNA-122 in mice by systemically administered LNA-antimiR leads to up-regulation of a large set of predicted target mRNAs in the liver. Nucleic Acids Res 2008;36:1153–1162.
[25] Ruf B, Greten TF, Korangy F. Innate lymphoid cells and innate-like T cells in cancer—at the crossroads of innate and adaptive immunity. Nat Rev Cancer 2023;23:351–371.
[26] Hammerich L, Tacke F. Hepatic inflammatory responses in liver fibrosis. Nat Rev Gastroenterol Hepatol 2023;20:633–646.
[27] Liepelt A, Tacke F. Stromal cell-derived factor-1 (SDF-1) as a target in liver diseases. Am J Physiol Gastrointest Liver Physiol 2016;311:G203–G209.
[28] Krones E, Erwa W, Trauner M, et al. Serum alkaline phosphatase levels accurately reflect cholestasis in mice. Hepatology 2015;62:981–983.
[29] Schmidt-Arras D, Rose-John S. IL-6 pathway in the liver: from physiopathology to therapy. J Hepatol 2016;64:1403–1415.
[30] Wei X, Liu H, Li X, et al. Over-expression of MiR-122 promotes apoptosis of hepatocellular carcinoma via targeting TLR4. Ann Hepatol 2019;18:869–878.
[31] Ravichandran G, Neumann K, Berkhout LK, et al. Interferon-γ-dependent immune responses contribute to the pathogenesis of sclerosing cholangitis in mice. J Hepatol 2019;71:773–782.
[32] Yamamoto M, Sato S, Hemmi H, et al. Essential role for TIRAP in activation of the signalling cascade shared by TLR2 and TLR4. Nature 2002;420:324–329.
[33] Schneider KM, Kummen M, Trivedi PJ, et al. Role of microbiome in autoimmune liver diseases. Hepatology 2024;80:965–987.
[34] Shi L, Zheng X, Fan Y, et al. The contribution of miR-122 to the innate immunity by regulating toll-like receptor 4 in hepatoma cells. BMC Gastroenterol 2019;19:130.
[35] Sun L, Jiang Z, Acosta-Rodriguez VA, et al. HCFC2 is needed for IRF1- and IRF2-dependent Tlr3 transcription and for survival during viral infections. J Exp Med 2017;214:3263–3277.
[36] Covelli C, Sacchi D, Sarcognato S, et al. Pathology of autoimmune hepatitis. Pathologica 2021;113:185–193.
[37] Parveen S, Tiwari A, Ashraf A. Histology activity index: a benchmark for decision making. Hepatol Int 2022;16:479.
[38] Seki E, De Minicis S, Osterreicher CH, et al. TLR4 enhances TGF-beta signaling and hepatic fibrosis. Nat Med 2007;13:1324–1332.
[39] Dapito DH, Mencin A, Gwak G-Y, et al. Promotion of hepatocellular carcinoma by the intestinal microbiota and TLR4. Cancer Cell 2012;21:504–516.
[40] Friedman SL, Pinzani M. Hepatic fibrosis 2022: unmet needs and a blueprint for the future. Hepatology 2022;75:473–488.
[41] Sun Y, Wang H, Li Y, et al. miR-24 and miR-122 negatively regulate the transforming growth factor-β/Smad signaling pathway in skeletal muscle dibrosis. Mol Ther Nucleic Acids 2018;11:528–537.
[42] Efremova M, Vento-Tormo M, Teichmann SA, et al. CellPhoneDB: inferring cell–cell communication from combined expression of multi-subunit ligand–receptor complexes. Nat Protoc 2020;15:1484–1506.
[43] Seki E, De Minicis S, Gwak G-Y, et al. CCR1 and CCR5 promote hepatic fibrosis in mice. J Clin Invest 2009;119:1858–1870.
[44] Barashi N, Weiss ID, Wald O, et al. Inflammation-induced hepatocellular carcinoma is dependent on CCR5 in mice. Hepatology 2013;58:1021–1030.
[45] Song Z, Chen W, Athavale D, et al. Osteopontin takes center stage in chronic liver disease. Hepatology 2021;73:1594–1608.

Keywords: Immune homeostasis; Autoimmune liver disease; Innate immunity; Chronic liver diseases; Fibrotic pathways; Pro-inflammatory cytokines; Immune checkpoints; Macrophage activation; Toll-like receptors (TLRs); NF-κB signaling; Immune activation; Gut–liver axis; Microbiome.
Received 18 March 2026; received in revised form 21 August 2026; accepted 26 August 2026; Available online 29 August 2026

Journal of Hepatology, Volume *8*

Supplemental information

MiR-122 regulates liver tolerance

Maytal Gefen, Shanny Layani, Emma Klahr, Zohar Shemuelian, Johannes Brandi, Thomas Jacobs, Christiane Steeg, Lorenz Adlung, Christoph Kilian, Merav Ordan, Oren Gordon, Nathalie Abudi, Rinat Abramovitch, Yuval Nevo, Sharona Elgavish, Hadar Benyamini, Jonathan Monin, Elina Zorde Khvalevsky, Nofar Rosenberg, Aurelia Markezana, Dayana Yaish, Adi S. Yehezkel, Alina Simerzin, Mila Rivkin, Osher Amran, Daniel S. Goldenberg, Amnon Peled, Jonathan Axelrod, Jacob Rachmilewitz, Samuel Huber, Nicola Gagliani, Christoph Schramm, Sören Weidemann, Johannes Herkel, Antonella Carambia, Rifaat Safadi, Abed Khalaileh, Ashraf Imam, Daniela Heide, Jenny Hetzer, Stefan Rose-John, Mathias Heikenwälder, Hilla Giladi, and Eithan Galun

MiR-122 regulates liver tolerance

Maytal Gefen, Shanny Layani, Emma Klahr, Zohar Shemuelian, Johannes Brandi, Thomas Jacobs, Christiane Steeg, Lorenz Adlung, Christoph Kilian, Merav Ordan, Oren Gordon, Nathalie Abudi, Rinat Abramovitch, Yuval Nevo, Sharona Elgavish, Hadar Benyamini, Jonathan Monin, Elina Zorde Khvalevsky, Nofar Rosenberg, Aurelia Markezana, Dayana Yaish, Adi S. Yehezkel, Alina Simerzin, Mila Rivkin, Osher Amran, Daniel S. Goldenberg, Amnon Peled, Jonathan Axelrod, Jacob Rachmilewitz, Samuel Huber, Nicola Gagliani, Christoph Schramm, Sören Weidemann, Johannes Herkel, Antonella Carambia, Rifaat Safadi, Abed Halila, Asraf Imam, Daniela Heide, Jenny Hetzer, Stefan Rose-John, Mathias Heikenwälder, Hilla Giladi, Eithan Galun

Table of contents

Supplementary materials and methods…………………………………………….....…..2

Supplementary tables……………………………………………………………....…..17

Supplementary figure legends…………………………………………..………....22

Supplementary figures…………………………………………………………………….27

Supplementary materials and methods

Mice

miR-122 knockout (KO) mice (C57BL/6miR-122em2Hhtg) were generated in collaboration with Prof. Dr. Nicola Gagliani and Prof. Dr. Samuel Huber at the University Medical Center Hamburg-Eppendorf, Hamburg, Germany. The genomic region of miR-122 (chr18:65,381,932-65,381,997) was targeted with 2 sgRNA guide sequences (sgRNA1:AACAGACGTCAAGTCACGCGTGG, sgRNA2:AGTGTGATAATGGCGTTTGATGG) generating a 60bp deletion. All mice were screened by genotyping the miR-122 genomic region by PCR analysis (for primer sequences, see Table S1). Two separate colonies generated by separate founders were maintained for experimentation. Male mice were used in all the experiments. Mice were maintained under specific pathogen-free (SPF) conditions in an animal facility with a temperature of approximately 23°C in a 12-hour light-dark cycle and received sterile commercial rodent chow and water ad libitum unless otherwise indicated. Maintenance of mice and all experimental procedures were performed by the Institutional Animal Care and Use Committee approved animal treatment protocols (license number OPRR-A01– 5011). All animal experiments were performed after a stringent ethical consideration and approval by the Hebrew University Of Jerusalem, Israel. The experiments were regulated by veterinarian specialists.

Alb-iGP_Smarta mice[51] were bred and maintained under specific pathogen-free conditions at the animal facility of the University Medical Centre Hamburg-Eppendorf (Hamburg, Germany). All animals received humane care, and clinical and behavioral humane endpoints were applied to minimize any harm. All animal experiments were conducted in accordance with the Basel Declaration, the European Directive 2010/63/EU, and German federal law.

Experiments were approved by the relevant review board and authorities of the State of Hamburg, Germany.

In Vivo experiments (procedures in live mice)

Magnetic Resonance Imaging (MRI) monitoring

MRI experiments were performed at the Wohl Institute for Translational Medicine at Hadassah Hebrew University Medical Center. MRI images were acquired on a 7T 24 cm bore, cryogen-free MR scanner based on the proprietary dry magnet technology (MR Solutions, Guildford, UK) using 38-mm inner diameter, 70 mm length mouse whole body volume coil. For MRI acquisition, mice were anesthetized with isoflurane vaporized with O_2. Isoflurane is used at 3.0% for induction and at 1.0 - 2.0% for maintenance. The mice were positioned on a heated bed, which allowed for continuous anesthesia and breathing rate monitoring throughout the entire scan period. Coronal T1-weighted (T1W) spin-echo images were acquired for anatomical segmentation purposes (repetition time = 1100 milliseconds, echo time = 11 milliseconds, FOV= 60mm, in-plane resolution = 230 μm, slice thickness = 1 mm). Coronal and axial T2-weighted (T2W) spin-echo images were also acquired for liver pathologies assessment (repetition time = 2500 ms, echo time = 45 ms, in-plane resolution = 230 μm, slice thickness = 1 mm).

Inhibition of miR-122 in a model of acute inflammation

For inhibition of miR-122, 7 to 8 weeks old, male, C57BL/6 mice, purchased from Harlan Laboratories (Jerusalem, Israel), were hydrodynamic tail vain injected (HTVI) with antagomiR-122 or antagomiR-control (7 μg/mouse in 1.5 mL saline, Sigma-Aldrich, St Louis, MO).

antagomiR-122	5'-mAsmCsmAmAmAmCmAmCmCmAmUmUmGmUmCmAmCmAmCmUsmCsmCsmAs-Chol-3'

antagomiR-control	5'-mCsmAsmCmCmAmCmAmUmAmCmCmGmCmAsmCs mGsmGs-Chol-3'

Twenty-four hours post HTVI, the mice were injected subcutaneously (SC) with LPS (1 µg/kg, L2630, Sigma-Aldrich St Louis, MO) and 5 hours later, the mice were sacrificed and their livers, were flash frozen in liquid nitrogen or in optimum cutting temperature (OCT) embedded frozen blocks, for further RNA and histologic analysis.

Macrophage depletion

For the depletion of macrophages, mice were injected with 200µl Clodronate Liposomes (Liposoma, The Netherlands), or control liposomes (PBS) as control (Liposoma, The Netherlands), by intraperitoneal (IP) injection. Starting at the age of 2 weeks, mice were injected at days 0, 2, 7, 14, 21 and then livers were harvested on day 28 of the experiment, at the age of 6 weeks.

Bacteremia model

Methicillin-resistant Staphylococcus aureus (MRSA) bacteremia model: The USA300 S. aureus strain SAP231 (derived from the community-acquired MRSA strain NRS384, which was isolated from an outbreak in the Mississippi prison system) was used and was generated as described previously[52]. SAP231 was streaked on tryptic soy agar (TSA) plates (tryptic soy broth [TSB] plus 1.5% Bacto agar [BD Biosciences]) and grown overnight at 37°C in a bacterial incubator. Two or three colonies were picked and cultured in TSB at 37°C in a shaking incubator (LAB-LINE) at 240 rpm overnight (18 h), followed by a 1:50 subculture at 37°C for 2 h to obtain mid-logarithmic-phase bacteria. The bacteria were pelleted, washed two times, and resuspended in sterile phosphate-buffered saline (PBS) at the indicated concentrations for each model below. The absorbance at 600 nm (A600) was measured to estimate the number of CFU, which was verified after overnight culture on TSA plates. All animal experiments were approved by the Hebrew University of Jerusalem Animal Care and

Use Committee. The S. aureus bacteremia model was modified from a previously described protocol[53]. Briefly, WT or miR1-22 KO 6-week-old male C57BL/6 mice inoculated IP with 5 x 10^7 CFUs of SAP231 in a 100-mL volume of PBS using a 29-gauge insulin syringe. Clinical score was determined by measuring weight and body temperature. To measure bacterial burden, enumeration of CFU was performed with overnight plate cultures of homogenized tissue (TissueLyser LT; QIAGEN). Livers were harvested 18 hours following infection. The CFU were counted after overnight culture with serial dilutions on TSA plates.

Malaria infection

For mouse malaria parasitemia model, miR-122 KO mice breeding pairs were sent to The Bernhard Nocht Institute for Tropical Medicine (BNITM), Hamburg, Germany. There they were crossed and bred with C57BL/6J mice at the animal facility of the BNITM, Hamburg, Germany in individual ventilated cages (IVC) with food and water ad libidum, to establish a miR-122 KO colony. All animal experiments were performed in accordance with German law and registered with the "Behörde für Gesundheit und Verbraucherschutz (BGV)". Stabilates with erythrocytes from Plasmodium berghei C57BL/6 mice were stored in 0.9% NaCl, 4.6% sorbitol and 35% glycerol in liquid nitrogen. In order to increase viability of the parasites, they were passaged once. To this end, parasites were thawed, diluted in 0.9% NaCL and i.p. injected into C57BL/6J mice. Fresh blood of passage mice, taken 5 days post infection, was used to transfer 1x 105 infected red blood cells/200 µl PBS i.p. into mice used for experiments.

Cerebral symptoms were monitored daily and scored according to the following scheme: 0 = no symptoms; 1 = decreased activity, deceleration; 2 = ataxia; 3 = weight loss≥ 20% or convulsions, strong ataxia; 4 = coma; 5 = death. If a score of ≥3 was reached, mice were euthanized in order to avoid unnecessary suffering.

Microbiome depletion

For microbiome depletion at the Hebrew University animal farm, breeding pairs were treated with vancomycin (500 mg/L), imipenem (500 mg/L) and neomycin (1 g/L) in drinking water as antibiotic cocktail, and the antibiotics-containing water was changed twice a week. Pups, born to parents treated with antibiotics, were treated continuously, also after weaning with the same antibiotics, up to the age of 6 weeks when the mice were harvested. Microbiome bacterial load was determined by measuring fecal DNA (ZymoBiomics DNA miniprep kit, D4300 Xymo, USA)

T cell depletion

For the depletion of T cells, mice were injected with 200ug anti CD4 (clone GK1.5, cat#BE0003-1, Bioxcell) and 200μg anti CD8 (clone 2.43, cat#BE0061, Bioxcell) or 400μg IgG isotype control (clone LTF-2, cat#BE0090, Bioxcell), diluted as recommended by the manufacturer in InVivoPure pH 7.0 dilution buffer (cat#IP0070, Bioxcell) or in InVivoPure pH 6.5 dilution buffer (cat# IP0065, Bioxcell). Mice were injected IP at the age of 2 weeks. Following the first injection, mice were injected with 100μg anti CD4 and 100μg anti CD8, or 200μg IgG as control, by IP injection at days 7, 14, 21 and then livers were harvested on day 28 of the experiment, at the age of 6 weeks.

Mice tissue handling

For all *in vivo* mice experiments, livers were extracted and weighed, and liver tumors with a diameter >2 mm were counted and recorded. Liver specimens were either fixed in 4% buffered formalin or snap-frozen in liquid nitrogen for further analysis. Serum was obtained from whole blood and either analyzed immediately or stored at -80°C for further analysis.

Mice blood chemistry

Mice liver enzymes were measured by COBAS 600 using the appropriate kits for detection of ALP (Alkaline Phosphatase acc. to IFCC Gen.2, Roche), SGPT (Alanine Aminotransferase acc. to IFCC without pyridoxal phosphate activation, Roche) and SGOT (Aspartate

Aminotransferase acc. to IFCC without pyridoxal phosphate activation, Roche), LDH (Lactate Dehydrogenase optimized, Roche). Hematology was measured by XS-1000i ™ Automated Hematology Analyzer (Sysmex). Determination of different white blood cell types was performed manually by a qualified veterinarian.

Mice histopathological analysis

Liver samples were fixed in 4% paraformaldehyde and paraffin-embedded (FFPE). Briefly, tissue was cut into 5µm sections from FFPE and stained with Hematoxylin/Eosin or Sirus red (Direct Red 80, Sigma-Aldrich, Rehovot, Israel) or IHC primary antibodies (Table S2). Epitope retrieval was obtained by using citrate buffer or Pronase (1mg/ml, P8038, Sigma-Aldrich, St Louis, MO). For IHC staining, we used a conjugated horseradish peroxidase secondary Ab (anti-mouse and -rabbit [Envision; Dako, Denmark] and antirat [Histifine; Nichirei, Osaka, Japan]) for 1 hour. All slides were counterstained with Mayer's hematoxylin and mounted. Cell death/apoptosis analysis was performed by TUNEL assay according to the manufacturer's protocol (ab206386, Abcam, Cambridge, United Kingdom). Negative and positive controls were included. For quantifications of staining, slides were scanned using an SCN400 slide scanner (Leica, Germany) and analyzed using the ImageJ software. Images of stained sections were quantified as the percentage of positively stained area per high power field or as the number of positively stained cells per field in 20µm or 40µm magnification, in 8 to 20 pictures per sample, using ImageJ, RRID: SCR_003070.

Flow cytometry

Isolation of hepatic immune cells: cells were isolated from liver and spleen of WT and miR-122 KO animals 6 weeks of age. Briefly, the animals were directly perfused with PBS with 2% FCS through the vena cava. A part of the liver was removed for histology, the remaining liver was cut into small pieces and digested in Liver digest Medium (Cat# 17703034, ThermoFisher Scientific/ Gibco, Waltham, Massachusetts) for 30 minutes at 37°C. Following

this incubation step, the liver pieces were forced through a 100µm cell strainer and washed with PBS with 2% FCS. The resulting suspension was centrifuged at 40 x g, 4°C for 5 minutes and the supernatant was collected and spun again at 400 x g, 4°C, 5 min. The cell pellet was resuspended in 40% Percoll (Cytiva, Sweden) and centrifuged at 650 x g for 20 min. at room temperature. The supernatant was discarded, and the pellet washed with PBS with 2% FCS. Afterwards, the pellet was resuspended in Ammonium-Chloride-Potassium (ACK) lysis buffer and incubated for 5 minutes. Lysis was stopped by the addition of PBS with 2% FCS. Cells washed in PBS with 2% FCS and then counted.

Flow cytometry analysis: A detailed description of the staining protocol was previously reported[54]. In brief, cells were adjusted for 1.5 x 10^6 cells and incubated with Fc-block (TruStain FcX™ PLUS Block (Cat#156604, BioLegend, San Diego, California). The surface staining was performed in Brilliant stain buffer (Cat# 563794, BD Bioscience, Franklin Lakes, New Jersey) with antibodies as detailed in Table S3. For intracellular staining, cells were fixated, permeabilized and stained using the eBioscience FoxP3/ Transcription Factor Staining Buffer Set (Cat#00-5523-00, ThermoFisher). For intracellular staining, antibodies detailed in Table S3 were used. For compensation, Ultra Comp eBeads (Cat 01-2222-41, ThermoFisher), were used and stained simultaneously with their respective antibody. After staining, cells were washed with PBS with 2% FCS. Cells were then fixated with 4% PFA and washed again with PBS with 2% FCS and kept on 4°C until they were acquired on a 5L Aurora (Cytek Biosciences, Bethesda, Maryland).

Cell culture

HEK293 Human embryonic kidney cells, and RAW264.7 mouse macrophage cells, were cultured in DMEM supplemented with 10% fetal calf serum (FCS), 1% penicillin/streptomycin (Thermo Scientific, Waltham, MA, USA). All cells were cultured at 37°C in a humidified atmosphere containing 5% CO_2.

Transfection procedures

Transfection of plasmids was performed with Lipofectamine 2000 transfection reagent (Cat#11668027; Invitrogen, Carlsbad, CA, USA) according to the manufacturer's protocol and using serum-free medium (Opti-MEM; Cat#31985070; Thermo Scientific). For luciferase assays, Hek293 cells in 24 well plates were transfected (20nM). Transfection of mimic miR-122 and scrambled control was performed with HiPerFect transfection reagent (Cat # 301705, QIAGEN) according to the manufacturer's protocol. RAW264.7 cells in 24 wells plates were transfected (50nM).

Sequence mimic miR-122:

sense	/5Phos/rUrGrGrArGrUrGrUrGrArCrArArUrGrGrUrGrUmUmUrG
antisense	mAmArCmArCmCrAmUrUmGrUmCrAmCrAmCrUmCrCA

Sequence mimic-miR, random scra:

sense	/5Phos/rGrCrGrArCrUrArUrArCrGrCrGrCrArArUrArUmGmGrU
antisense	mCmArUmArUmUrGmCrGmCrGmUrAmUrAmGrUmCrGC

Construction of miR-122 reporter plasmids and Luciferase activity assay

A fragment from 3'UTR of IRF2 gene containing a putative miR-122 target, was synthesized by PCR amplification and was then cloned in the pmirGLO vector between the SacI–SalI sites. The mutated miR-122 binding site in the 3'-UTR of the human IRF2, was created using a mutated fragment by two PCR steps. For the first step, wt IRF2-3'UTR was used as template and mutated forward primer (carrying a mutated site 2 and a SphI restriction site) were used for amplification. The second PCR was performed using the isolated fragment from PCR 1 as the reverse primer. The isolated product of the second PCR was digested with SacI and SalI and ligated to the pmiRGLO vector. The positive control AldoA reporter plasmid was generated similarly. The 3'-UTR of human AldoA was PCR-amplified and was

then cloned in the pmirGLO vector between the SacI–XbaI sites (for primer sequences, see Table S1)**.**

The Following transfections (24hr or 48hr), the cells were lysed with passive lysis buffer (Cat#E1941; Promega) according to manufacturer's instructions. Firefly and Renilla luciferase activity was assessed using the Dual Luciferase Reporter Assay system (Cat#E1910; Promega) on a luminometer Mithras 2000 (Centro XZ, LB960, Berthold Technologies, Bad Wildbad, Germany). The luciferase activity was normalized to Renilla luciferase activity. Readings were taken in triplicates.

TLR4 inhibition

Inhibition of TLR4 *in vitro* in Raw264.7 cells was performed as previously reported[55,56]. CLI-095 inhibitor (0.5µg/ml 10%DMSO, InvivoGen, France) was added to the culture medium one hour prior to macrophage activation with lipopolysaccharides (LPS).

Macrophage activation with LPS

Activation of RAW264.7 murine macrophages was performed as previously reported with LPS-EB Ultrapure (100ng/ml or 5ng/ml, tlrl-3pelps, InvivoGen, France). LPS-EB Ultrapure was added to the culture medium. 24 hours post activation the medium and the cells were collected for further analysis.

RNA Extraction

Total RNA was isolated from mice liver tissues samples, or from cells grown in culture, using Trizol reagent (Life Technologies, Paisley, UK). In the first day of extraction, 1ml Trizol was added to 50 mg of murine tissue and homogenized, or added to cells grown in monolayer. The lysates were incubated for 5 min at RT. 200µl Chloroform were added to the Trizol-containing lysates, mixed by vortexing for 20sec and incubated for 3min at RT. After that, the samples were centrifuged for 15min at 14000 x g at 4°C, the upper aqueous phase was transferred into fresh tube and the RNA was precipitated in 100% Ethanol at -20°C

overnight (ON). Next day, the samples were centrifuged for 40min at 14000 x g at 4°C. The pellets were washed (X2) with 85% cold ethanol and re-dissolved in 50µl DDW-DEPC for 10 min at 65°C . RNA yield and quantity were determined using a Nanodrop spectrophotometer ND-1000 (Thermo scientific, Wilmington, DE). For RNAseq samples only, RNA was extracted using MaXtract High Density (QIAGEN, Redwood City, CA) according to the manufacturer's instructions. Integrity of the RNA was analyzed by a ND-1000 V3.7.1, according to the manufacturer's instructions. Only samples with an RNA integrity number (RIN) of 9 or greater were sequenced.

Quantitative real time RT-PCR

cDNA was synthesized from total RNA using the Quanta Biosciences qScript ™ cDNA Synthesis Kit (Cat#95047-100; Quanta Biosciences, Beverly, MA, USA) for mRNA analysis, and using the qScript™ microRNA cDNA Synthesis Kit (Cat#95107-100) for microRNAs analysis. Quantitative PCR (qPCR) of microRNAs and mRNA was performed using the SYBR Green PCR Kit (Cat#84018 and Cat#84071, Quanta Biosciences, Beverly, MA, USA). The fold expression and statistical significance were calculated using the $2^{-\Delta\Delta Ct}$ method. All experiments were performed in triplicates. Primers used for Real-Time-PCR are shown in Table S1. Target mRNAs were normalized to HPRT. Target microRNAs were normalized to U6 or miR-39.

RNA-Seq

Quality control measures were applied to raw FASTQ files, including trimming of low-quality bases and residual adapters as well as filtering out short reads. These measures were performed with Trim_Galore v0.6.1 wrapper tool https://github.com/FelixKrueger/TrimGalore. File assessment before and after quality control was conducted with FastQC v0.11.8 http://www.bioinformatics.babraham.ac.uk/projects/fastqc. Subsequently, reads were aligned

to the transcriptome GRCm38 [mm10] utilizing Salmon v1.10.0, a tool for fast and bias-aware quantification of transcript expression[57]. Differential gene expression analysis was performed with DESeq2 v1.40.2[58]. Based on differential genes lists, heatmaps and volcano diagrams are plotted with standard R functions and packages. Gene set enrichment analysis for pathways and GO terms was performed with Broad Institute GSEA utility V4.2.2: GSEA software, and Molecular Signature Database (MSigDB)[59]. For this analysis, genes were ranked by their signed t-statistics as adopted from the differential gene expression step. Gene sets include mouse GO terms, wiki-pathway and Hallmark sets.

Single Cell analysis

For isolation of single cells, livers were perfused with PBS and harvested in cold PBS. A 50mg portion was collected in disassociation buffer (0.15% Collagenase P (Roche) in HBSS) and incubated at 37˚c until complete disassociation. Cells were treated with ACK for the removal of red blood cells. Cells were washed and strained and kept in full growth medium (DMEM supplemented with 10% fetal calf serum (FCS) (Thermo Scientific, Waltham, MA, USA)). Dead cells were removed using Dead Cell Removal Kit (Miltenyi Biotec, Auburn, USA) according to the manufacturer protocol. Cells were counted manually, and dead cell percentages were calculated using trypan blue. Only samples with less than 10% dead cells were used for sequencing. The cells were subjected to the Chromium Next GEM Single Cell 3′ GEM, Library & Gel Bead Kit v3.1 (10× Genomics, CA, USA) and libraries were prepared for sequencing following manufacturer company instructions. Sequencing was done using NovaSeq 6000 system machine (Illumina) with the following sequencing conditions: 28 bp (Read1) and 90 bp (Read2) using the NovaSeq 6000 SP Reagent Kit v1.5 (100 cycles) (Illumina). Raw FASTQ files were quantified with cellranger count utility, V7.1.0, by 10x Genomics, and mapped to mm10 reference. Filtered barcode-feature matrices were next analyzed with Seurat package, V5.0.3, R package designed for QC, analysis, and exploration

of single-cell RNA-seq data[60]. Quality control measures included the exclusion of cells with an exceedingly low number of expressed genes, a high proportion of mitochondrial gene content, or extreme read counts (either too low or too high). After quality control, cell clustering was conducted using Seurat pipelines. Multiple clustering profiles were generated and evaluated. The prominent clustering scheme integrated treated and untreated samples at weeks 2 and 6 and utilizing the SCT transform method. Additionally, a separate clustering profile focused exclusively on samples from week 2. Cell types were affiliated to clusters using a set of predefined gene markers. Stain plots are based on normalized expression data and a three-color scheme which maps to low, average, and above average expressions. Differentially expressed genes between clusters or between samples or between cell subsets were resolved with Seurat FindMarkers utility. These sets of genes were then submitted for GSEA analysis as described earlier in the RNA-Seq section. Gene network interactions were explored with CellphoneDB, V4.0.0, repository of curated receptors, ligands and their interactions and a tool to interrogate single-cell transcriptomics data[61]. Tables of p-values and interaction strength were generated with CellphoneDB statistical analysis method and further analyzed with custom R scripts.

ELISA

Proteins were measured in serum or measured in the medium of treated cells by ELISA according to the manufacturer's instructions. CXCL1 (ab216951, Abcam, Cambridge, United Kingdom), IL6 (ab222503, Abcam, Cambridge, United Kingdom), EPO (442707, Biolegend, San Diego, California), SAA (DY2948-05, R&D systems Minneapolis).

Human data Patient selection and inclusion

The human autoimmune hepatitis (AIH) clinical data included in this study was approved by the local ethics committee (Approval number PV4081) and was conducted in accordance

with the Declaration of Helsinki. All patients gave their written informed consent for data sharing and tissue analysis.

Inclusion Criteria:

Initial Diagnose of AIH

Age > 18

consent

Exclusion Criteria:

treatment with Glucocorticoide or Anti-TNF-AK at the time of

sampling (Hydroxychloroquin allowed -> 1 Patient)

AIH/PBC Overlap and other causes of acute hepatitis

Normal liver tissue FFPE material was provided from material stored in pathology that was not used for diagnostic purposes and is allowed to use without consent according to the UKE, Hamburg, Germany regulations.

Clinical assessment of AIH patients

Clinical scoring of Inflammation activity and Fibroisgrade were assessed as we previously reported[62].

Gene expression analysis of AIH patient liver biopsies

Identification of differentially expressed genes from bulk mRNA-sequencing data was performed from normalized counts processed with DESeq2 package[58] by Wilcoxon rank-sum test with post-hoc correction for multiple testing. Gene set enrichments were analyzed with gprofiler2[63].

microRNA sequencing of human liver samples

RNA extracted from liver samples of AIH patients was sequenced by Qaigen according to the following discerption. Sample preparation: A DNase digestion was carried out and the RNA eluted with 14 µl RNase free water. RNA concertation and quality were measured by Qubit

RNA BR (20-1000 ng) and RNA ScreenTape (25-500 ng/µl). Library preparation and sequencing: The library preparation was done using the QIAseq miRNA Library Kit (QIAGEN). A total of 100 ng RNA was converted into miRNA NGS libraries. After adapter ligation, UMIs were introduced in the reverse transcription step. The cDNA was amplified using PCR (16 cycles) and during the PCR, indices were added. After PCR, the samples were purified. Library preparation was quality controlled using capillary electrophoresis (Tape D1000). Based on quality of the inserts and the concentration measurements, the libraries were pooled in equimolar ratios. The library pool(s) were quantified using qPCR. The library pool(s) were then sequenced on a NextSeq2000 (Illumina Inc.) sequencing instrument according to the manufacturer instructions (1x75, 1x8). Raw data was de-multiplexed and FASTQ files for each sample were generated using the bcl2fastq2 software (Illumina inc. USA). Read mapping, and quantification of gene expression: All primary analysis was carried out using CLC Genomics Server 21.0.4. The workflow "QIAseq miRNA Quantification" of CLC Genomics Server with standard parameters was used to map the reads to miRBase version 22. In short, the reads were processed by (1) trimming of the common sequence, UMI and adapters, and (2) filtering of reads with length < 15 nt or length > 55 nt. They were then deduplicated using their UMI. Reads are grouped into UMI groups as follows: (1) when they start at the same position based on the end of the read to which the UMI is ligated (i.e., Read2 for paired data), (2) when they are from the same strand, and (3) they have identical UMIs. Groups that contain only one read (singletons) are merged into non-singleton groups if the singleton's UMI can be converted to a UMI of a non-singleton group by introducing an SNP (the biggest group is chosen). All reads that don't map to miRBase, neither with perfect matches nor as isomiRs (maximum 2 mismatches and/or alternative start/end position of 2 nt), were mapped to the human genome GRCh38 with ENSEMBL GRCh38 version 97 annotation. This was carried out using the "RNA-Seq

Analysis" workflow of CLC Genomics Server with standard parameters. The 'Empirical analysis of DGE' algorithm of the CLC Genomics Workbench 21.0.4 was used for differential expression analysis with default settings. It is an implementation of the 'Exact Test' for two-group comparisons developed by Robinson and Smyth, 2008 and incorporated in the EdgeR Bioconductor package Robinson et al., 2010. For all unsupervised analysis, only miRNAs were considered with at least 10 counts summed over all samples. A variance stabilizing transformation was performed on the raw count matrix using the function vst of the R package DESeq2 version 1.28.1. 500 genes with the highest variance were used for the principal component analysis. The variance was calculated agnostically to the pre-defined groups (blind=TRUE). 35 genes with the highest variance across samples were selected for hierarchical clustering.

Statistical analysis

Data were evaluated for significance by two-tailed Student t-test or Mann–Whitney test, as indicated and unless otherwise noted. $P \leq 0.05$ was considered significant for all analyses. Calculations were performed using GraphPad Prism 6.02 software (GraphPad Software, Inc).

Supplementary tables

Table S1. Primers

gene		sequence	manufacturer
miR-122 genomic region	F	GTTTGGAACCACTGAGGAGTCTGTGAC	IDT-syntezza
	R	CCATAGGAGAGGTTGGATTTTACTGGTC	IDT-syntezza
Pri-miR-122	F	TTCGGGAACTATGTGGAGTCACTTTG	IDT-syntezza
	R	CGCAAGGCTGCCCTCAAACCCTCAG	IDT-syntezza
IRF2 3'UTR	F	CACAGAGCTCAGAACATTGATTGCTGTGTC	IDT-syntezza
	R	CACAGTCGACGGATGGGATAGGAAGAGAGG	IDT-syntezza
Mutated IRF2 3'UTR	F	ATCTTGCTCTATCTCACAGCATGCTAAGCACCCCCTTCTCTG	IDT-syntezza
AldoA 3'UTR	F	TAACCAGAGCTCCTCTTCGTCTCTAACCACG	IDT-syntezza
	R	ACCAATCTAGAGCTGTTTATTTGGCAG	IDT-syntezza
αSMA	F	GTCCCAGACATCAGGGAGTAA	IDT-syntezza
	R	TCGGATACTTCAGCGTCAGGA	IDT-syntezza
TGFβR2	F	CCGCTGCATATCGTCCTGTG	IDT-syntezza
	R	AGTGGATGGATGGTCCTATTACA	IDT-syntezza
Col3a1	F	CCTGGCTCAAATGGCTCAC	IDT-syntezza
	R	CAGGACTGCCGTTATTCCCG	IDT-syntezza
Col4a3	F	CAAAGGCATCAGGGGAATAACT	IDT-syntezza
	R	ATCCGTTGCATCCTGGTAAAC	IDT-syntezza
CCR1	F	CTCATGCAGCATAGGAGGCTT	IDT-syntezza
	R	ACATGGCATCACCAAAAATCCA	IDT-syntezza
CCR5	F	TTTTCAAGGGTCAGTTCCGAC	IDT-syntezza
	R	GGAAGACCATCATGTTACCCAC	IDT-syntezza
CXCl12	F	TGCATCAGTGACGGTAAACCA	IDT-syntezza
	R	TTCTTCAGCCGTGCAACAATC	IDT-syntezza
CXCR4	F	CCATGGAACCGATCAGTGTGA	IDT-syntezza
	R	TTGCCGACTATGCCAGTCAAG	IDT-syntezza
TNFα	F	GAAAAGCAAGCAGCCAACCA	IDT-syntezza
	R	CGGATCATGCTTTCTGTGCTC	IDT-syntezza
IL1β	F	TTCAGGCAGGCAGTATCACTC	IDT-syntezza
	R	GAAGGTCCACGGGAAAGACAC	IDT-syntezza
IL6	F	ATACCACTCCCAACAGACCTGTCT	IDT-syntezza
	R	CAGAATTGCCATTGCACAACTC	IDT-syntezza
IFNγ	F	CCACGGCACAGTCATTGAAAG	IDT-syntezza
	R	AGAGATAATCTGGCTCTGCAGG	IDT-syntezza
IL12a	F	CTGTGCCTTGGTAGCATCTATG	IDT-syntezza
	R	GCAGAGTCTCGCCATTATGATTC	IDT-syntezza
IL12b	F	TGGTTTGCCATCGTTTTGCTG	IDT-syntezza
	R	ACAGGTGAGGTTCACTGTTTCT	IDT-syntezza
TLR2	F	ACAACTTACCGAAACCTCAGAC	IDT-syntezza
	R	CACACACCCCAGAAGCATCACATG	IDT-syntezza
TLR3	F	GCGTTGCGAAGTGAAGAACTTG	IDT-syntezza
	R	GTTGTTCAAGAGGAGGGCGAAT	IDT-syntezza
TLR4	F	TTCAGAACTTCAGTGGCTGG	IDT-syntezza
	R	TGTTAGTCCAGAGAAACTTCCTG	IDT-syntezza
AldoA	F	GGGTGATCCTCTTCCACGAGA	IDT-syntezza
	R	AGGGGCACCACACCCTTATC	IDT-syntezza
CXCL1	F	CTGGGATTCACCTCAAGAACATC	IDT-syntezza
	R	CAGGGTCAAGGCAAGCCTC	IDT-syntezza
CXCL2	F	CCAACCACCAGGCTACAGG	IDT-syntezza
	R	GCGTCACACTCAAGCTCTG	IDT-syntezza
IRF2	F	AATTCCAATACGATACCAGGGCT	IDT-syntezza
	R	GAGCGGAGCATCCTTTTCCA	IDT-syntezza
Hamp	F	CCTATCTCCAGCAACAGATG	IDT-syntezza
	R	AACAGATACCACAGGAGGGT	IDT-syntezza
HJV	F	CAGCTCCCCGGTTTCGT	IDT-syntezza
	R	TGGTAGACTTTCTGGTCAATGCA	IDT-syntezza
SAA1	F	ACACCAGGATGAAGCTACTCACCA	IDT-syntezza
	R	CCCTTGGAAAGCCTCGTGAACAAA	IDT-syntezza
SAA2	F	CAGGAGACACCAGCAGGATGAAG	IDT-syntezza

	R	CCCGAGCATGGAAGTATTTGTCTC	IDT-syntezza
SPP1	F	GTGATTTGCTTTTGCCTGTTTG	IDT-syntezza
	R	GAGATTCTGCTTCTGAGATGGG	IDT-syntezza
CD8	F	GTTCAGCAAGGAAAACGAAGGC	IDT-syntezza
	R	GCACAGGTGAGGGAGTTCG	IDT-syntezza
HPRT	F	GCGATGATGAACCAGGTTATGA	IDT-syntezza
	R	ATCTCGAGCAAGTCTTTCAGTCCT	IDT-syntezza
miR-122-5p	F	HSA-MIR-122-5P PCR ASSAY 20-YP00205664	Qiagen
C.el. miR-39	F	CEL-MIR-39-3P MIRCURY PCR ASSAY 20-YP00203952	Qiagen
U6	F	U6 SNRNA (V2) MIRNA PCR ASSAY	Qiagen

Table S2. IHC primary antibodies

Antibody	Catalog	Company
F4/80	MCA497G	BioRad
CD3	MCA1477	BioRad
CD4	Ab183685	ABCAM
CD8	14-0808-82	Invitrogen
Ly6g	Ab238132	ABCAM
cleaved caspase 3	9661	Cell signaling
pSTAT3	D3A7	Cell Signaling
NFkB- P65	D14E12 xP(R)	Cell Signaling
IRF2	ab124744	ABCAM
SPP1	AKm2A1	Santa Cruz

Table S3. Antibodies for FACS analysis

Target	Clone	Fluorochrome	Vendor	Cat	RRID	Stock conc. mg/mL	Final conc. mg/ml	Dilution Factor	Staining condition
Viability	N/A	Zombie NIR	BioLegend	423106	N/A	N/A	N/A	1600	Extracellular
CD8α	53-6.7	Biotin Strep-AF350	BioLeg./ThermoFS	100703/S11249	AB_312742	0.5	0.0025	200	Extracellular
LAG-3 (CD223)	C9B7W	BUV737	BD Biosciences	741820	AB_2871155	0.2	0.008	25	Extracellular
Ly6G	1A8	PerCP	BioLegend	127653	AB_2616998	0.2	0.008	25	Extracellular
TIGIT	1G9	PE-Dazzle594	BioLegend	142110	AB_2566572	0.2	0.008	25	Extracellular
F4/80	QA17A29	PE-Fire640	BioLegend	157319	AB_2904297	0.2	0.008	25	Extracellular
CD160	7H1	PE	BioLegend	143003	AB_10960740	0.2	0.004	50	Extracellular
ICOS-L (CD275)	REA990	VioBright 515	Miltenyi	130-116-450	AB_2727547	0.15	0.003	50	Extracellular
CXCR3	CXCR-3173	BV650	BioLegend	126531	AB_2563160	0.2	0.004	50	Extracellular
XCR1	ZET	BV510	BioLegend	148218	AB_2565231	0.2	0.004	50	Extracellular
TCR gd	GL3	BUV563	BD Biosciences	748993	AB_2873390	0.2	0.002	100	Extracellular
CD127	A7R34	PE-Cy5	ThermoFisher Scientific	15-1271-83	AB_468794	0.2	0.002	100	Extracellular

CD27	LG.3A10	BUV496	BD Biosciences	741094	AB_2870693	0.2	0.002	100	Extracellular
BTLA (CD272)	6A6	PE-Fire700	BioLegend	N/A	N/A	0.18	0.0018	100	Extracellular
PD-L1 (CD274)	MIH5	PerCP-eF710	ThermoFisher Scientific	46-5982-80	AB_2573818	0.2	0.002	100	Extracellular
PD-1 (CD279)	29F.1A12	BV605	BioLegend	135220	AB_2562616	0.2	0.002	100	Extracellular
TIM-3 (CD366)	RMT3-23	BV711	BioLegend	119727	AB_2716208	0.2	0.002	100	Extracellular
CD39	Y23-1185	BUV395	BD Biosciences	567264	N/A	0.2	0.002	100	Extracellular
CD62L	MEL-14	BV570	BioLegend	104433	AB_10900262	0.025	0.00025	100	Extracellular
CD69	H1.2F3	BUV615	BD Biosciences	751593	AB_2875587	0.2	0.002	100	Extracellular
CD45	30-F11	SparkViolet538	BioLegend	103179	AB_2890675	0.5	0.005	100	Extracellular
PVR (CD155)	TX56	BUV661	BD Biosciences	749991	AB_2874213	0.2	0.002	100	Extracellular
CD11c	N418	SparkBlue550	BioLegend	117365	AB_2860633	0.5	0.0025	200	Extracellular
CD19	6D5	SparkNIR685	BioLegend	115567	AB_2819828	0.5	0.0025	200	Extracellular
CD44	IM7	AF700	BioLegend	103026	AB_493713	0.5	0.0025	200	Extracellular
Galectin-9	108A2	PE-Cy7	Biolegend	137913	AB_2750156	0.2	0.001	200	Extracellular
KLRG-1	2F1	APC-eF780	ThermoFisher Scientific	47-5893-82	AB_2573988	0.2	0.001	200	Extracellular
Ly6C	HK1.4	BV785	BioLegend	128041	AB_2565852	0.2	0.001	200	Extracellular
NK1.1	S17016D	APC/Fire810	BioLegend	156519	AB_2894654	0.2	0.001	200	Extracellular
Siglec-H	eBio440	SuperBright436	ThermoFisher Scientific	62-0333-82	AB_2784802	0.2	0.001	200	Extracellular
CD11b	M1/70	SparkYG593	BioLegend	101281	AB_2892261	0.5	0.00125	400	Extracellular
CD25	PC61	BV480	BD Biosciences	566202	AB_2739522	0.2	0.0005	400	Extracellular
ICOS (CD278)	C398.4A	BV750	BioLegend	313557	AB_2876616	0.15	0.000375	400	Extracellular
CD4	GK1.5	SparkBlue574	BioLegend	100489	AB_2904268	0.5	0.00125	400	Extracellular
MHC II	M5/114.15.2	PE-Fire810	BioLegend	107667	AB_2894690	0.2	0.0005	400	Extracellular
FoxP3	REA788	Vio R667	Miltenyi	130-111-682	AB_2651766	0.2	0.004	50	Intracellular
CD152 (CTLA-4)	UC10-4B9	BV421	BioLegend	106312	AB_2563063	0.2	0.004	50	Intracellular
Granzyme B	GB11	Pacific Blue	BioLegend	515408	AB_2562196	0.1	0.001	100	Intracellular
Perforin	S16009A	APC	BioLegend	154304	AB_2721463	0.2	0.002	100	Intracellular
Ki67	SolA15	BUV805	ThermoFisher Scientific	368-5698-82	N/A	0.2	0.002	100	Intracellular
CD3	17A2	FITC	BioLegend	100203	AB_312660	0.5	0.00125	400	Intracellular

<u>Table S4. miR-122-5p targets that control fibrosis & autoimmunity</u>

miR-122 target (m -mouse; h - human)	2 weeks padj	**2 weeks ko/wt**	3 weeks padj	**3 weeks ko/wt**	6 weeks padj	**6 weeks ko/wt**	Symbol	Fibrosis	Autoimmune	Name	References (PMID)
m	6.16E-05	**1.8**	9.13E-05	**1.7**	2.97E-35	**5.5**	Adamts 2	v		ADAM Metallo-peptidase With Thrombospondin Type 1 Motif 2	17929299
m & h	3.72E-42	**3.9**	9.55E-37	**3.5**	7.86E-26	**2.9**	Aldoa	v		Aldolase, Fructose-Bisphosphate A	38443347
m & h	3.07E-20	**4.1**	2.40E-24	**4.7**	1.10E-33	**6.3**	Alpl	v		Alkaline Phosphatase, Biomineralization Associated / Tissue Non-Specific Alkaline Phosphatase (Tnap)	31969558
m	5.06E-05	**1.5**	3.38E-10	**1.8**	6.48E-30	**2.9**	Bak1		v	BCL2 Antagonist/Killer 1	19282307
m & h	2.48E-107	**3.9**	1.98E-79	**3.3**	2.57E-63	**3.3**	Bckdk		v	Branched Chain Keto Acid Dehydrogenase Kinase	28597552
m	5.48E-10	**8.6**	6.25E-08	**5.8**	1.91E-12	**30.0**	Bmp8b	v		Bone Morpho-genetic Protein 8b	32694734
m	1.13E-07	**1.9**	2.84E-11	**2.2**	1.20E-16	**3.0**	Capn5		v	Calpain 5	37207905
m	0.242	**1.5**	9.23E-05	**3.5**	1.81E-08	**6.1**	Ccr2	v		C-C Motif Chemo-kine Receptor 2	19441102
m	0.0017	**2.1**	2.06E-06	**3.0**	1.40E-10	**4.3**	Ciita		v	Class II Major Histocompatibilit y Complex Trans-activator	15808836
m	4.50E-24	**12.1**	6.91E-22	**11.8**	3.41E-26	**19.1**	Cldn7	v		Claudin 7	24737165
m & h	4.85E-18	**2.1**	1.33E-20	**2.2**	4.26E-41	**3.3**	Clic1	v		Chloride Intra-cellular Channel 1.	37924785
m	1.65E-08	**2.9**	3.38E-17	**4.7**	7.94E-46	**14.3**	Ddr1	v		Discoidin domain receptor family, member_1	36809965
m	0.000199	**2.2**	0.000694	**2.1**	2.81E-16	**7.1**	Fbln1	v		Fibulin 1	37674431
m	8.40E-27	**2.2**	8.42E-25	**2.1**	1.91E-47	**3.2**	Fuca2		v	Alpha-L-Fucosidase 2	39681237
m & h	3.46E-22	**3.8**	8.40E-18	**3.4**	5.95E-23	**4.9**	Ihh	v	v	Indian hedgehog	37035456, 37740690
m & h	4.61E-06	**1.5**	4.72E-10	**1.8**	4.39E-27	**2.7**	Iqgap1	v		IQ Motif Containing GTPase Activating Protein 1	35036058
m & h	2.83E-06	**1.8**	1.09E-14	**2.6**	9.80E-47	**5.8**	Klf6	v		Kruppel Like Factor 6; Core	28864835

										Promoter Element Binding Protein (Copeb)	
m	1.37E-58	**13.7**	3.34E-87	**25.9**	5.53E-103	**37.6**	Lcn2	v		Lipocalin 2	32968110
m	0.000244	**2.1**	4.73E-08	**2.9**	6.72E-18	**5.0**	Neat1	v	v	Nuclear Para-speckle Assembly Transcript 1 (lncRNA)	28864835, 37716986
m	2.93E-06	**3.3**	6.84E-09	**4.4**	1.32E-11	**6.0**	Pdgfb	v		Platelet Derived Growth Factor Subunit B	16842882
m	9.19E-07	**2.1**	0.0132	**1.5**	6.02E-11	**2.9**	Thbs2	v		Thrombospondin 2	38379585
m	4.73E-06	**2.1**	2.98E-05	**2.0**	2.66E-19	**4.2**	Trim47	v		Tripartite Motif Containing 47	29291351

Supplementary figure legends

Fig. S1. Clinical data and correlations of miR-122 expression in AIH patients A-B. miR-122 levels measured by RNAseq in RNA extracted from liver samples of patients with chronic autoimmune hepatitis (AIH), correlates negatively with clinical scoring of (A) Inflammation activity and (B) Fibrosis grade[64].

Fig. S2. Expression of miR-122 and precursor expression and secretion in mouse AIH model. Pri-miR-122 and miR-122 levels measured by RT-PCR in RNA extracted from liver and serum samples of late stage AIH mice 20-28 weeks old. The Primary transcript of miR-122 (primiR-122) as well as the mature miR-122 are significantly decreased as well as the levels of secreted miR-122 measured in the serum. * $p<0.05$, ** $p<0.01$

Fig. S3. miR-122 regulates TLR3 via IRF2. A. Experimental design- mice were injected by hydrodynamic tail vein injection (HTVI) with antagomiR-122 or antago-Scr (control). 24 hours post HTVI mice were injected IP with LPS. 5 hours later livers were harvested for analysis. **B**. miR-122 levels measured by RT-PCR in RNA extracted from harvested livers. **C.** Heatmap of differentially expressed genes (DEGs) in livers of mice were miR-122 was inhibited compared to control. **D**. Gene expression analysis of RNA from livers of mice where miR-122 was inhibited by antagomir-122 and antago-Scr control: Gene set enrichment analysis (GSEA). **E**. GSEA hallmarks of IFNα and IFNγ in mice were miR-122 is inhibited compared to control. **F**. Allegro motif discovery analysis. The mouse binding site of the STAT1::STAT2, IRF2, IRF1 transcription factors (TF) is very similar to the motif shown. **G**. Segal Lab algorithm (cut off < = - 10.00) score of these TFs in human. **H**. Binding potential score of miR-122 to targets in listed genes, including the validated miR-122 target AldoA. **I.** MiR-122 reporter assay: Regulation by miR-122 compared to miR-scr as measured by the luciferase signal of a reporter plasmid bearing the 3'UTR the validated miR-122 target

AldoA, human IRF2 (hIRF2) and hIRF2 where the binding site for miR-122 is mutated (hIRF2mut). **J.** IHC staining of IRF2 in liver tissue section from mice where miR-122 was inhibited compared to control (n=3). **K.** Relative gene expression of IRF2 gene in mice where miR-122 was inhibited compared to control. **L**. Schematic depicting the regulation of IRF2 transcription factor on the expression of TLR3 gene. Host cell factor C2 (HCFC2) facilitates IRF2 association with its DNA target. * $p<0.05$

Fig. S4. Generation of miR-122 KO mouse and phenotype follow-up. A. Depiction of the genetic modification performed on miR-122 KO mice. The sequences of miR-122-5p and miR-122-3p are indicated (blue) within the genomic region of miR-122 in the mouse, and specifically the seed sequence. Two sgRNA target sites (yellow)were utilized in order to create a ~60bp deletion and the cleavage site is indicated (red arrow). **B**. Genotyping was performed on DNA extracted from tissue samples of inbred mice using primers that span the genomic region of miR-122. The PCR amplicon of WT mice was of 380bp, while the amplicon from miR-122 KO mice was 320bp. **C.** Realtime PCR relative expression of miR-122-5p in RNA extracted from livers of miR-122 KO mice and WT controls at the age of 6 weeks. **D**. Directional gene heatmap – Fold change (FC) in livers of miR-122 KO mice compared to WT control from week 2 through week 3 to week 6. Fold change (FC) of miR-122 KO/ WT increases from A->C: Negative at A, around zero at B and positive at C. **E.** Routine MRI monitoring of miR-122 KO mice revealed the development of HCC tumors as early as the age of 35 weeks (representative MRI image and image of harvested liver- tumors indicated by yellow arrows and yellow outline). Mice bearing tumors were euthanized and a survival curve shows the tumor frequency in miR-122 KO mice and WT control mice up to the age of 78 weeks. **F.** Single cell analysis of cells isolated from livers of miR-122 KO mice and WT controls and the age of 2 weeks indicating the cells expressing HAMP in the Hepatocyte associated cluster. **G.** Real time PCR relative expression of Hamp and Hjv in

RNA extracted from livers of miR-122 KO mice and WT controls at the age of 6 weeks. * $p<0.05$, ** $p<0.01$, *** $p<0.001$, **** $p<0.0001$

Fig. S5. IL-12 expression is upregulated in effector CD8 T cells of miR-122 KO mice. A. Gene set enrichment analysis (GSEA) of differentially expressed genes within the single cell RNAseq CD25^{+} CD62L^{-} effector T cell cluster of miR-122 KO mice compared to WT control at the ages of 2 weeks and 6 weeks ranked by normalized enrichment score (NES) **B**. Spearman correlation between mRNA expression of IL-12A or IL-12B in liver biopsies of AIH patients and the corresponding histological Modified Hepatitis Activity Index (mHAI) score. **C**. Spearman correlation between mRNA expression of IL-12A or IL-12B in liver biopsies of AIH patients and the respective expression levels of miR-122. **D**. Analysis of single cell RNAseq data: expression of IL12a in the B cell cluster of miR-122 KO mice compared to WT control at the age of 6 weeks. IL12 expression is found in the B cell cluster (turquoise) and IL12Rb1 and IL12Rb2 in the T cell cluster (soft pink). ** $p<0.01$, *** $p<0.001$, **** $p<0.0001$

Fig. S6. T cell depletion in miR-122 KO mice. A. Experimental design- mice were injected IP with αCD4 and αCD8 or IgG control starting from the age of 2 weeks and harvested at the age of 6 weeks. **B.** liver histology of miR-122 KO T cell depleted mice and controls, T cells (CD3), Macrophages (F4/80) and fibrosis (Sirius red). **C.** Real time PCR relative gene expression measured in livers of miR-122 KO mice where T cells were depleted and controls. ** $p<0.01$, *** $p<0.001$

Fig. S7. Microbiome depletion alleviates the inflammatory phenotype in miR-122 KO mice. A. Experimental design- mice were treated with antibiotics cocktail (ABX) or DDW as control starting from before birth and harvested at the age of 6 weeks. **B.** Confirmation of microbiome depletion by measurement of Fecal DNA in feces samples of miR-122 KO mice

treated with ABX or DDW. **C.** Liver histology of miR-122 KO treated with ABX cocktail or DDW as control. Macrophages (F4/80) and Fibrosis (Sirius red). **D.** Real time PCR relative gene expression measured in livers of miR-122 KO treated with ABX or DDW as control of TLR4 and downstream mediators of inflammation TNFα, IL-1β, IL-6, IL-12a, IL12b and IFNγ. * p<0.05, ** p<0.01, *** p<0.001

Fig. S8. SAA significantly increases in miR-122 KO mice A. Gene expression analysis of RNAseq data from livers of miR-122 KO mice compared to WT controls at the ages of 2, 3 and 6 weeks indicating the significance (pv) and fold change (L2FC) of the genes SAA1 and SAA2. **B.** Real time PCR confirmation of differentially expressed SAA genes. **C.** SAA levels measured by ELISA in serum of miR-122 KO mice and WT controls at the age of 6 weeks. **D.** Single cell RNAseq analysis of livers of miR-122 KO mice and WT controls at the ages 2 weeks and 6 weeks indicating the clusters of cells expressing SAA1 and SAA2. * p<0.05, ** p<0.01, *** p<0.001

Fig. S9. Spp1 significantly increases in miR-122 KO mice. A. Spp1 Immunohistochemical staining in miR-122 KO mice and WT control at the age of 6 weeks. **B.** Gene expression analysis of RNAseq data from livers of miR-122 KO mice compared to WT controls at the ages of 2, 3 and 6 weeks indicating the significance (pv) and fold change (L2FC) of Spp1 gene. **C.** Real time PCR confirmation of Spp1 gene. **D.** Single cell RNAseq analysis of livers of miR-122 KO mice and WT controls at the age of 6 weeks indicating the clusters of cells expressing Spp1. * p<0.05, ** p<0.01.

Fig. S10. Clinical data of miR-122 KO mice bacterial infection compared to WT control. A. Clinical scoring of miR-122 KO mice compared to WT control infected with $5x10^7$ CFU MRSA or saline as control. Weight and body temp was documented prior to harvesting. **B**. Blood chemistry- liver damage markers (alkaline phosphatase (ALP), Lactate Dehydrogenase

(LDH), serum glutamic-oxaloacetic transaminase (SGOT) and Serum glutamic pyruvic transaminase (SGPT)) measured in the serum **C.** Hematology- white blood cells (WBC), Lymphocytes, Neutrophils and Monocytes absolute number of cells (Abs.) . * $p<0.05$, ** $p<0.01$, *** $p<0.001$, **** $p<0.0001$

Supplementary Figure 1. Clinical data and correlations of miR-122 expression in AIH patients

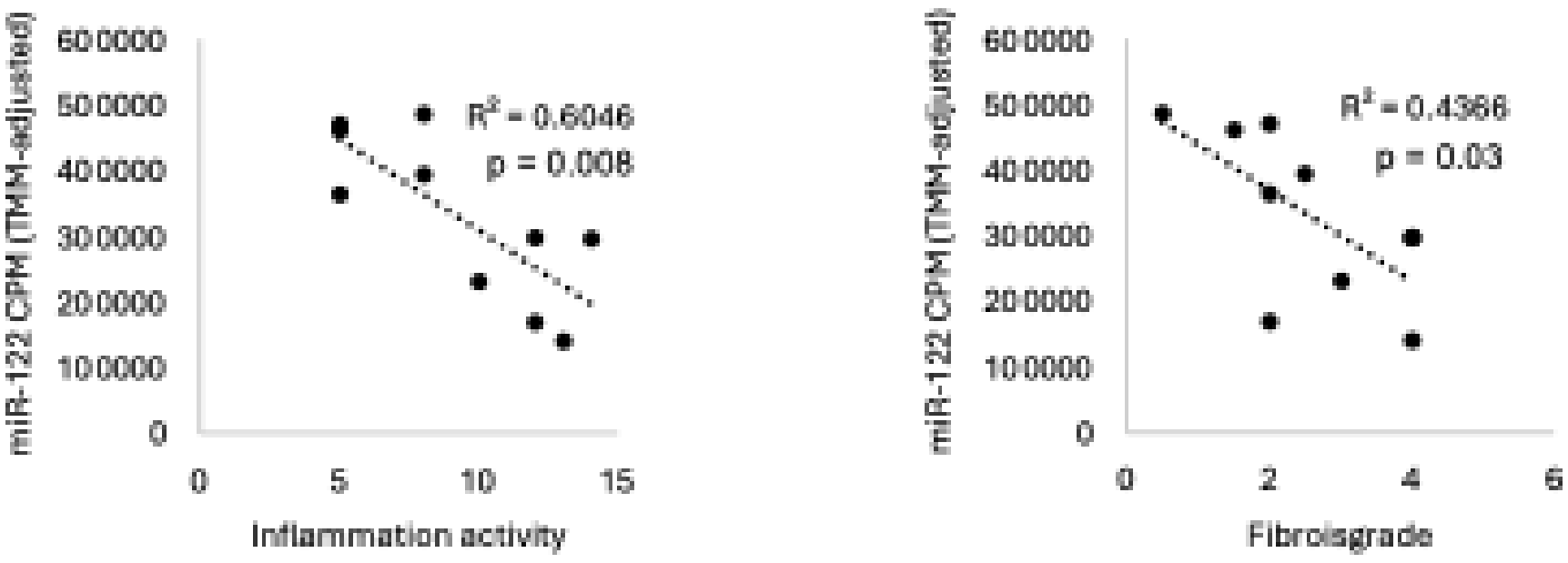

Supplementary Figure 2. Expression of miR-122 and precursor expression and secretion in mouse autoimmune liver disease model

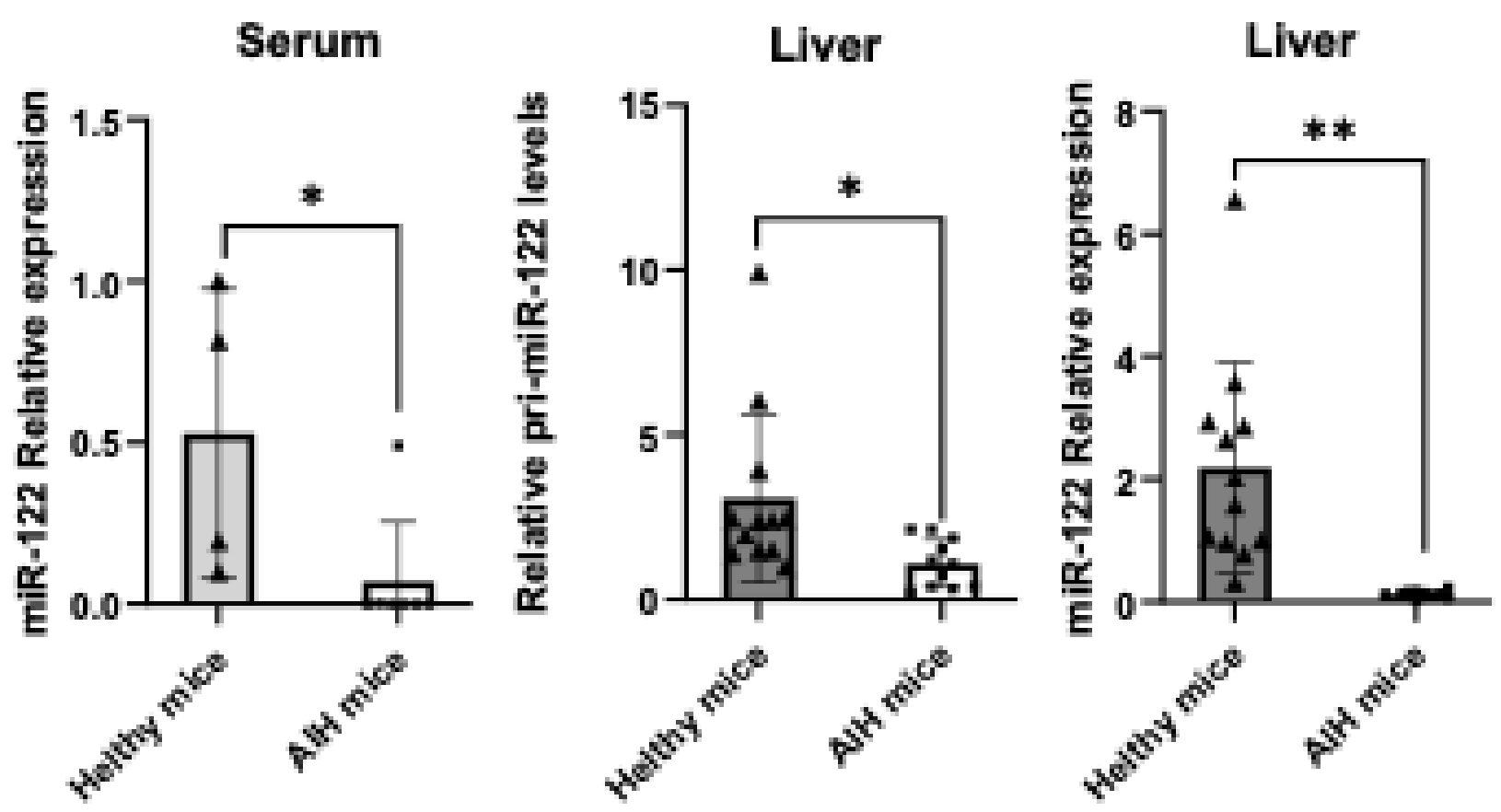

Supplementary Figure 3. miR-122 regulates TLR3 via IRF2

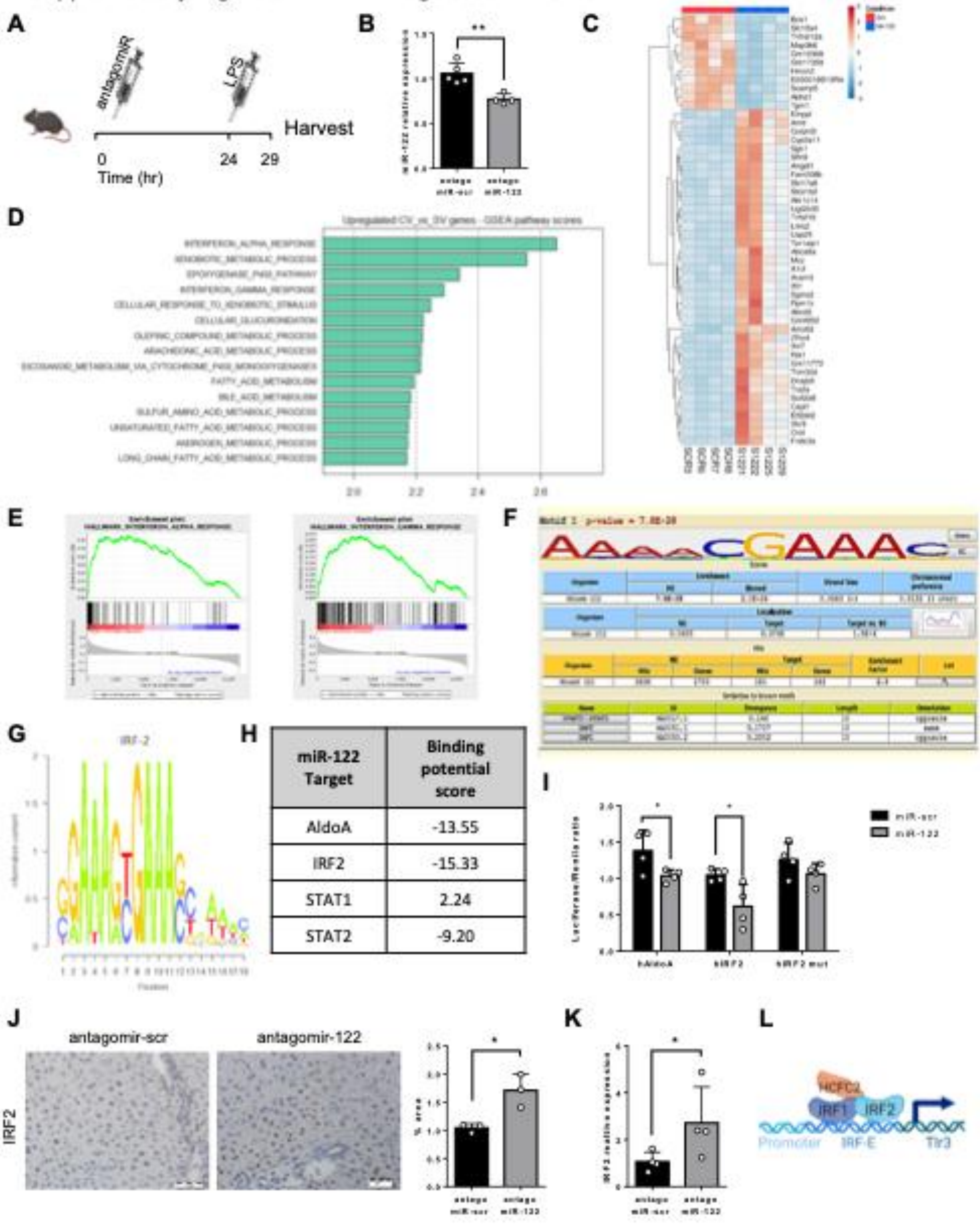

miR-122 Target	Binding potential score
AldoA	-13.55
IRF2	-15.33
STAT1	2.24
STAT2	-9.20

Supplementary Figure 4. Generation of miR-122 KO mouse and phenotype follow-up

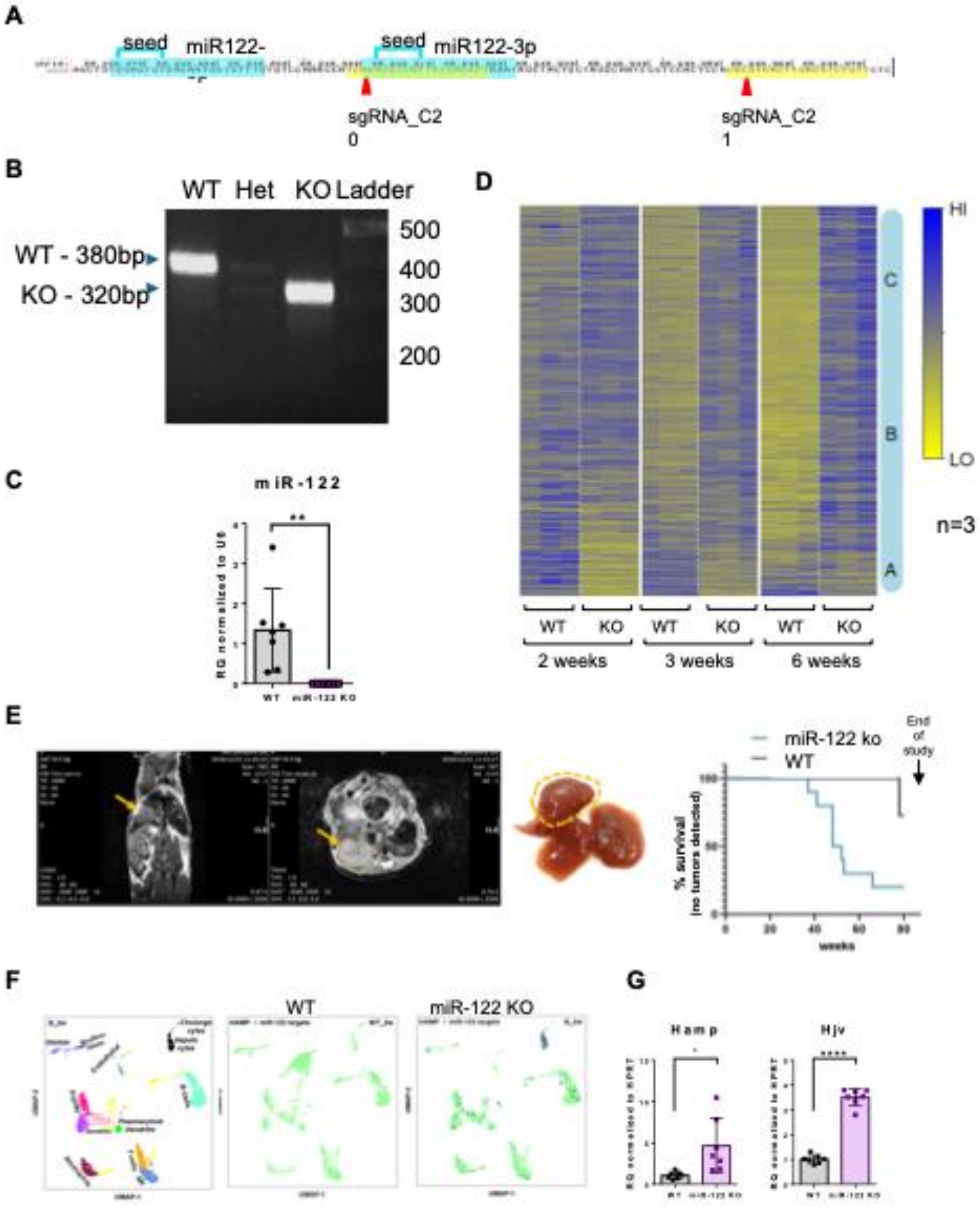

Supplementary Figure 5. IL-12 expression is upregulated in effector CD8 T cells of miR-122 KO mice

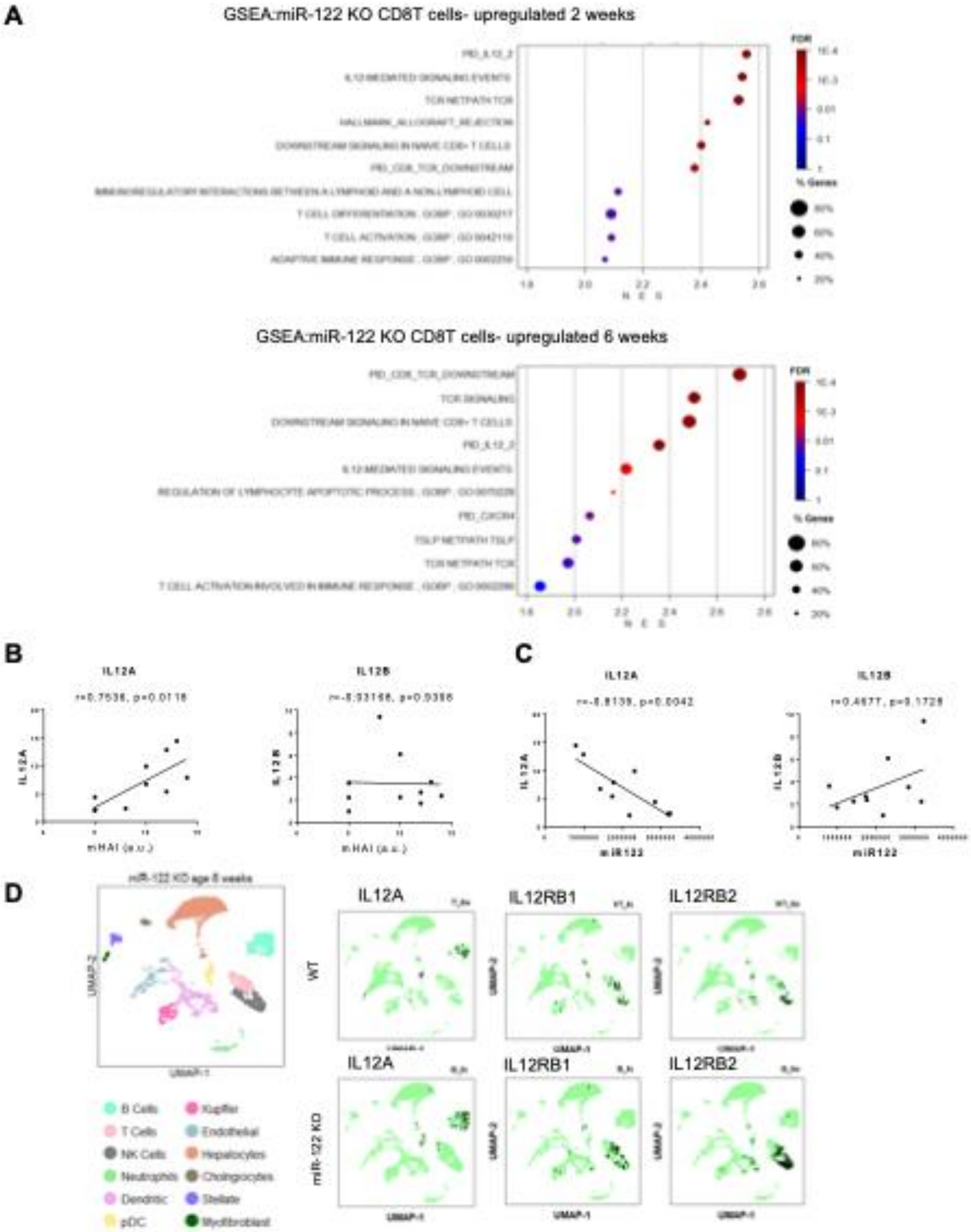

Supplementary Figure 6. T cell depletion in miR-122 KO mice

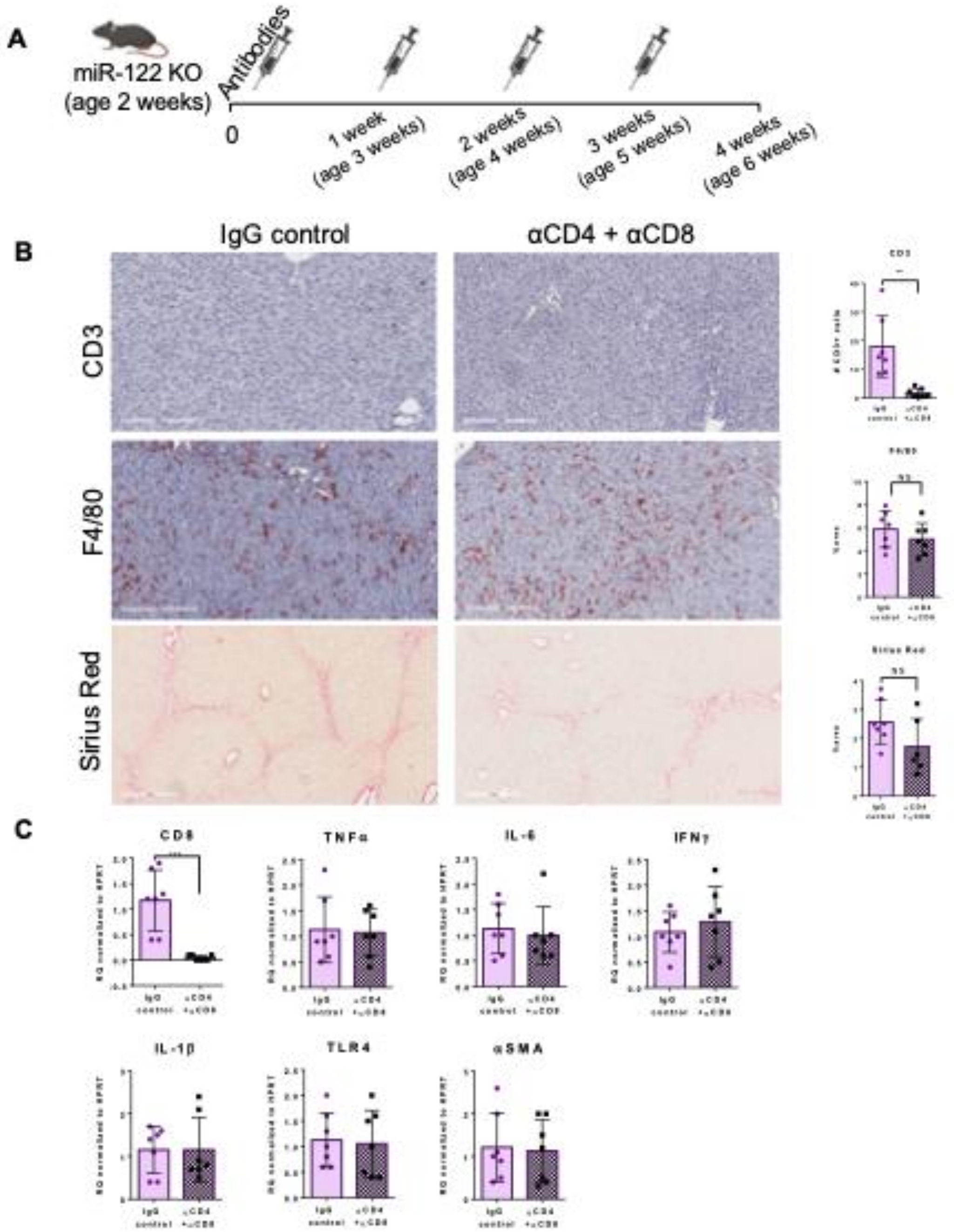

Supplementary Figure 7. Microbiome depletion alleviates the inflammatory phenotype in miR-122 KO mice

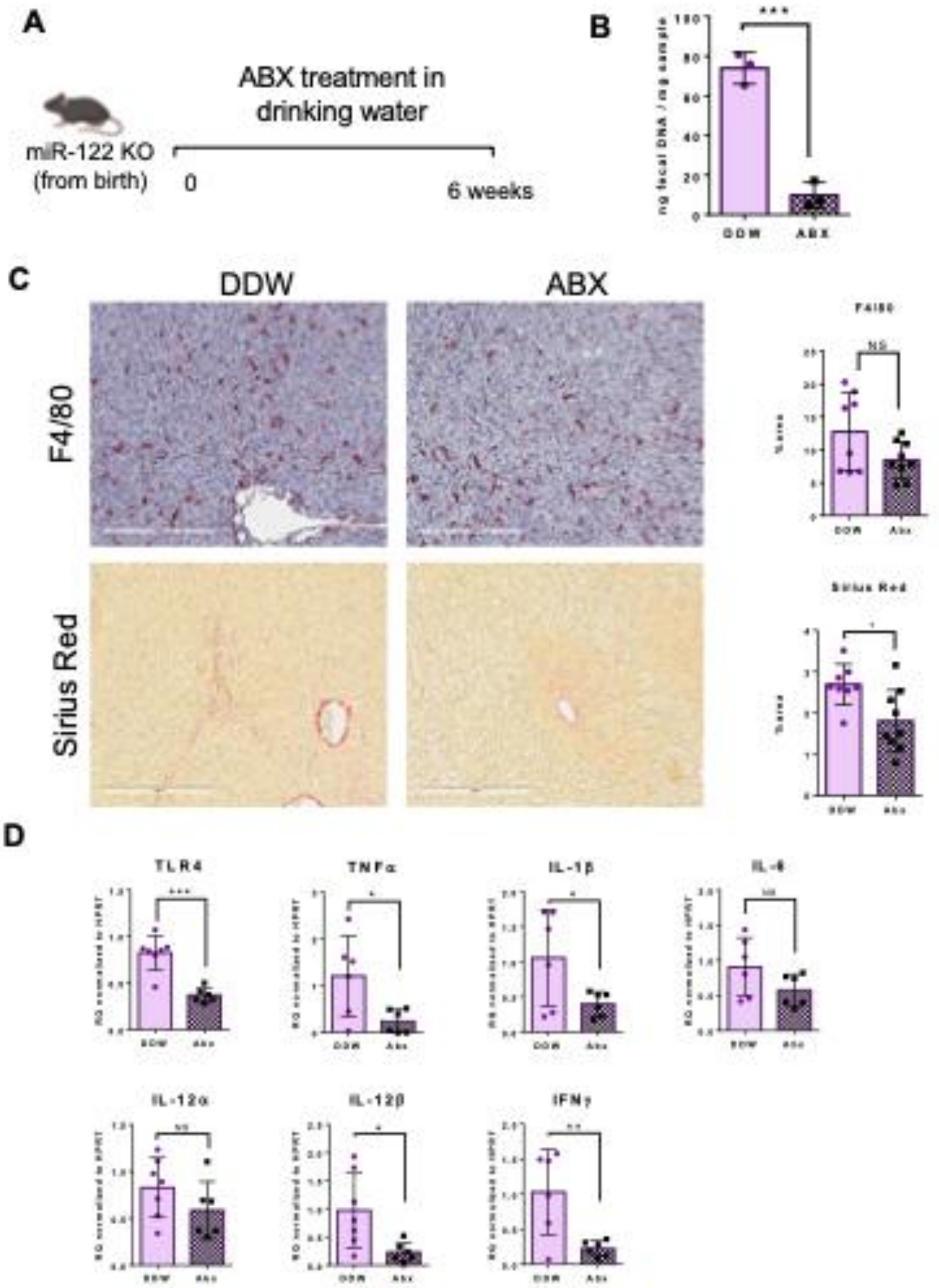

Supplementary Figure 8. SAA significantly increases in miR-122 KO mice

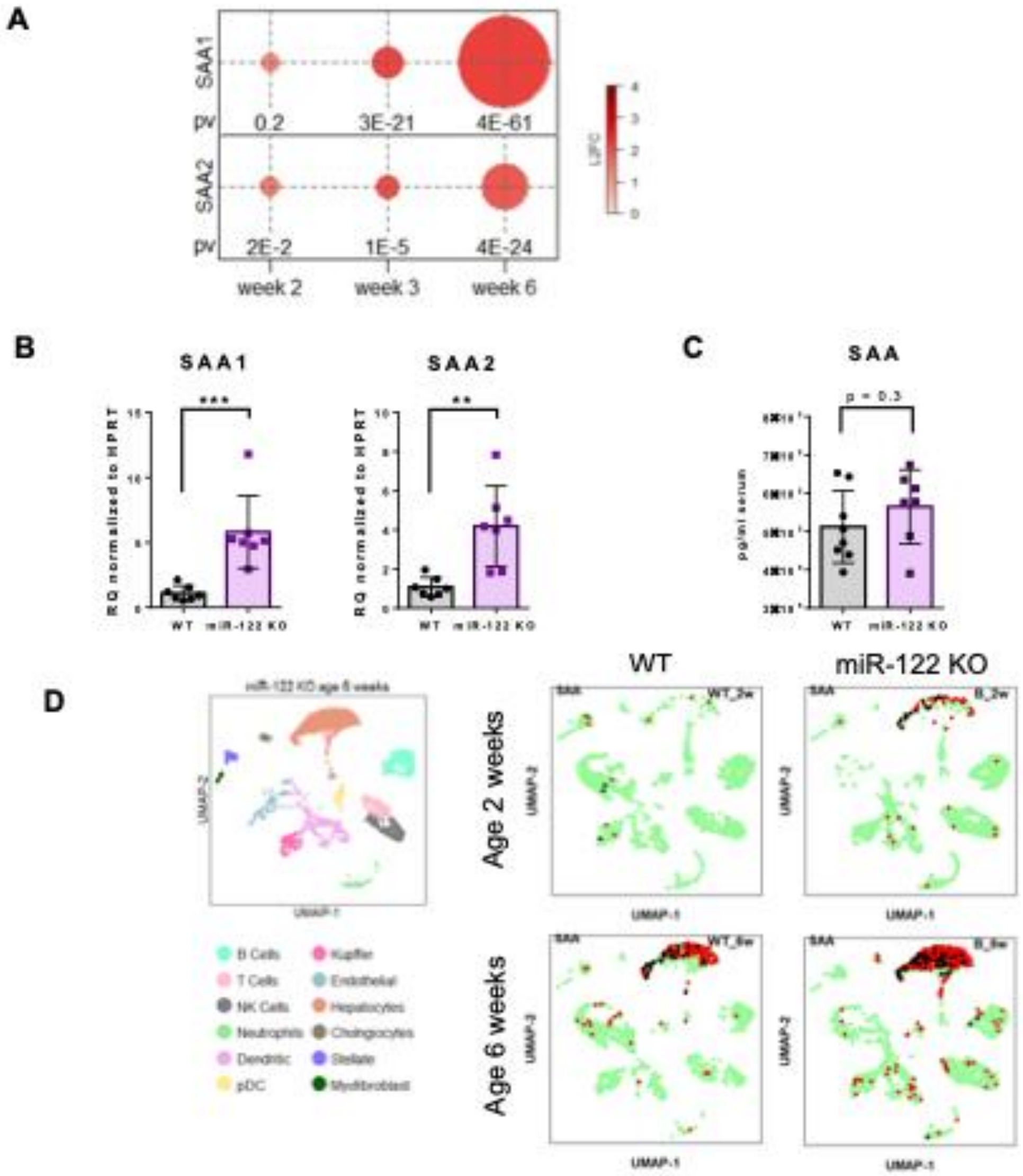

Supplementary Figure 9. Spp1 significantly increases in miR-122 KO mice

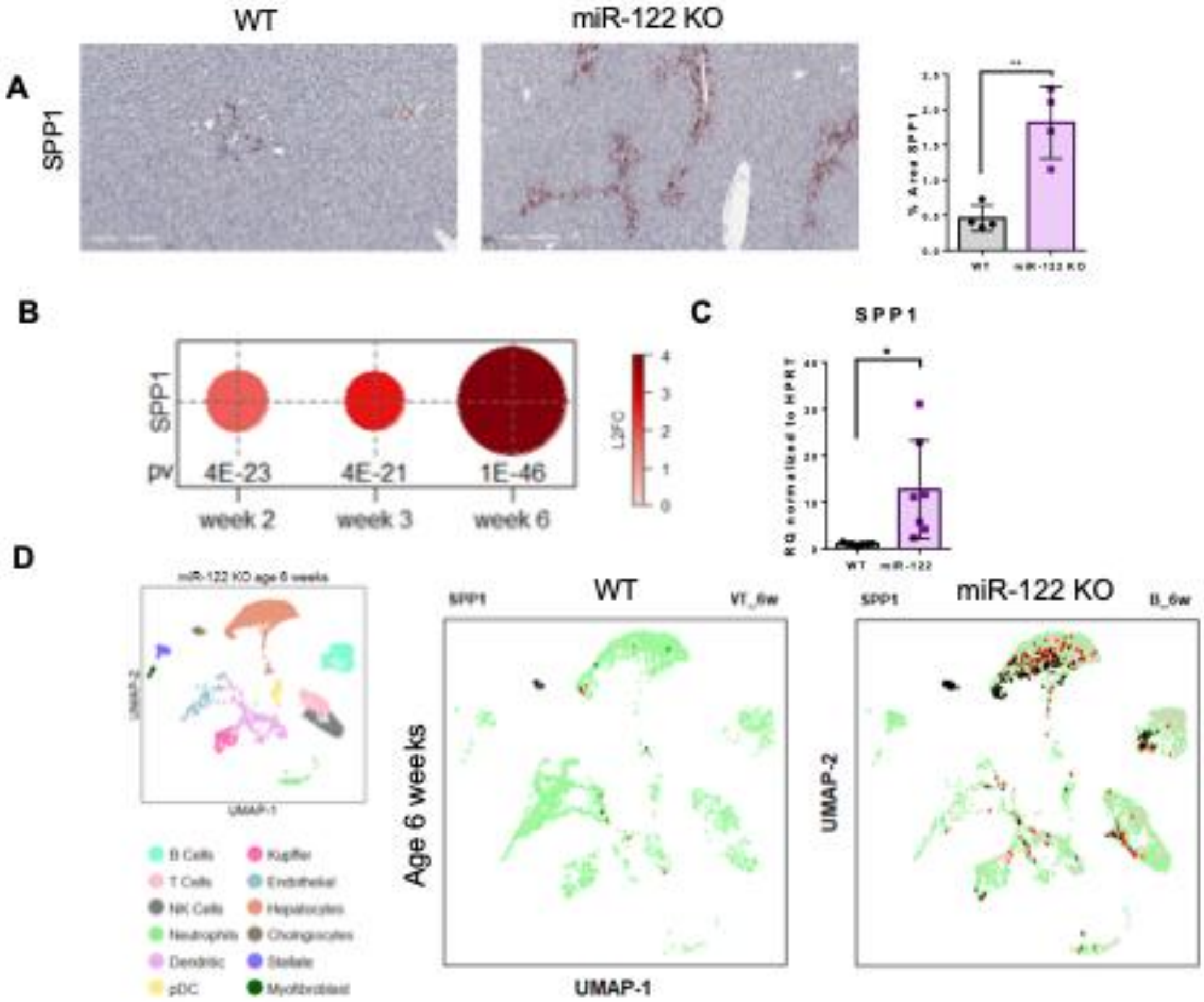

Supplementary Figure 10. Clinical data of miR-122 KO mice bacterial infection compared to WT control

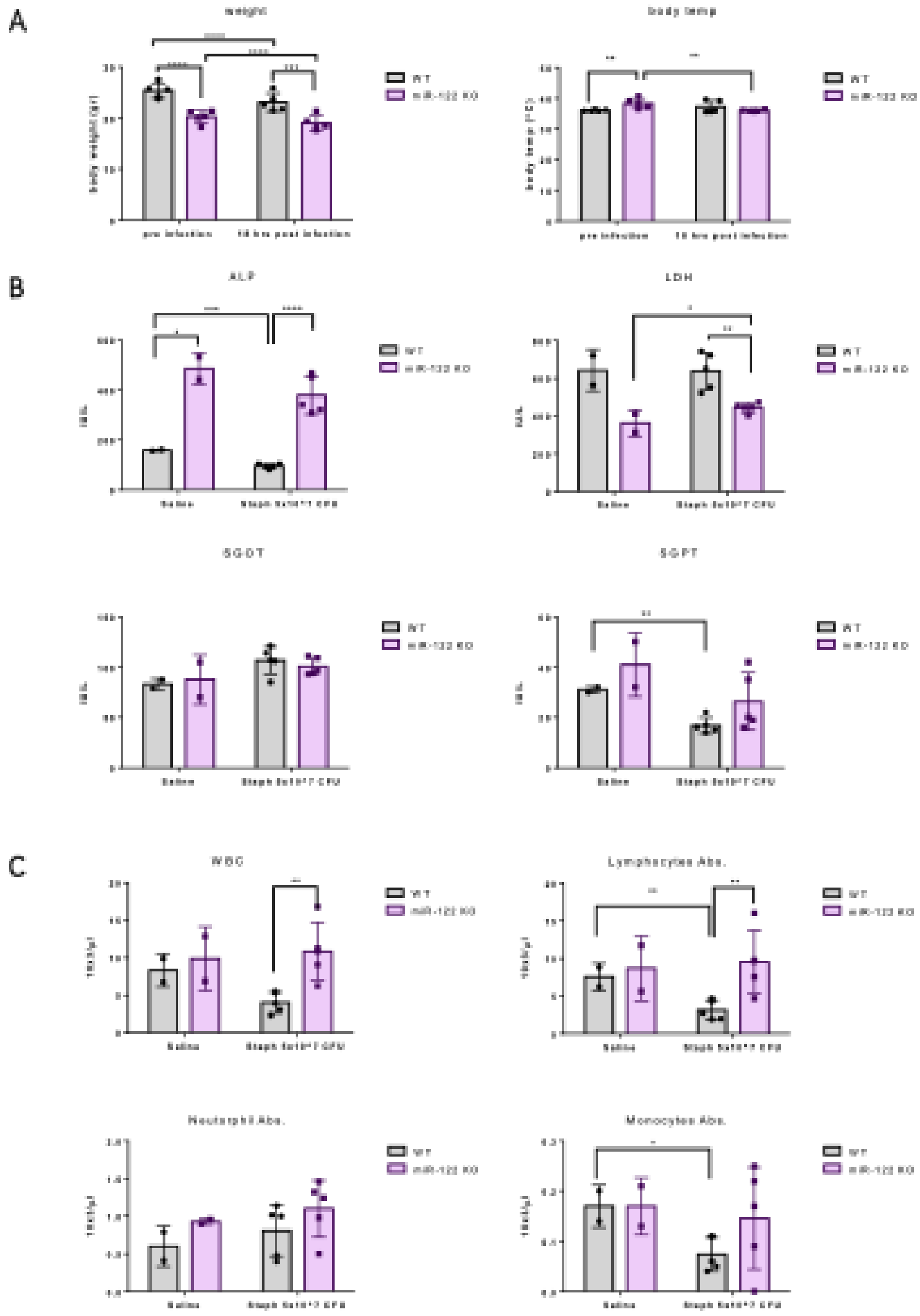

Supplement: Supplementary file 3 [file mmc4.pdf]
